# Reductive C–C Coupling from Molecular Au(I) Hydrocarbyl Complexes: A Mechanistic Study

**DOI:** 10.1021/jacs.0c11296

**Published:** 2021-02-05

**Authors:** Juan Miranda-Pizarro, Zhongwen Luo, Juan J. Moreno, Diane A. Dickie, Jesús Campos, T. Brent Gunnoe

**Affiliations:** † Instituto de Investigaciones Químicas (IIQ), Departamento de Química Inorgánica and Centro de Innovación en Química Avanzada (ORFEO−CINQA), Universidad de Sevilla and Consejo Superior de Investigaciones Científicas (CSIC), Avenida Américo Vespucio 49, 41092 Sevilla, Spain; ‡ Department of Chemistry, University of Virginia, Charlottesville, Virginia 22904, United States

## Abstract

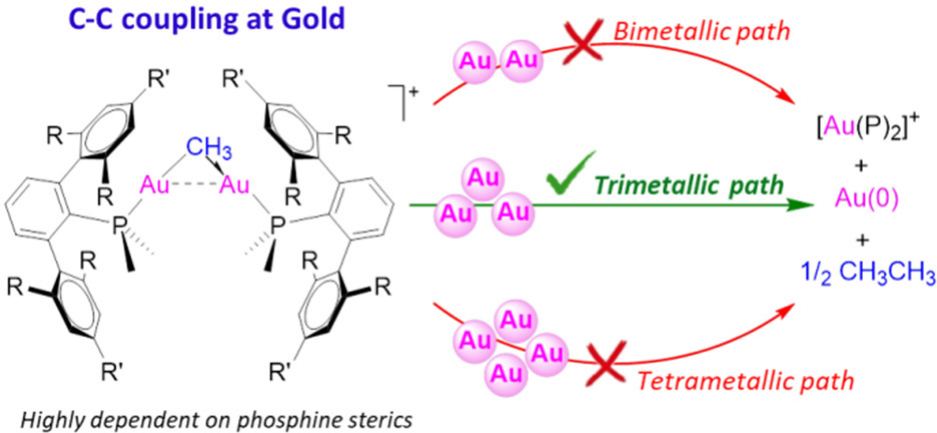

Organometallic gold complexes are used in a range of catalytic reactions, and they often serve as catalyst precursors that mediate C–C bond formation. In this study, we investigate C–C coupling to form ethane from various phosphine-ligated gem-digold(I) methyl complexes including [Au_2_(μ-CH_3_)(PMe_2_Ar′)_2_][NTf_2_], [Au_2_(μ-CH_3_)(XPhos)_2_][NTf_2_], and [Au_2_(μ-CH_3_)(^*t*^BuXPhos)_2_][NTf_2_] {Ar′ = C_6_H_3_-2,6-(C_6_H_3_-2,6-Me)_2_, C_6_H_3_-2,6-(C_6_H_2_-2,4,6-Me)_2_, C_6_H_3_-2,6-(C_6_H_3_-2,6-^*i*^Pr)_2_, or C_6_H_3_-2,6-(C_6_H_2_-2,4,6-^*i*^Pr)_2_; XPhos = 2-dicyclohexylphosphino-2′,4′,6′-triisopropylbiphenyl; ^*t*^BuXPhos = 2-di-*tert*-butylphosphino-2′,4′,6′-triisopropylbiphenyl; NTf_2_ = bis(trifluoromethyl sulfonylimide)}. The gem-digold methyl complexes are synthesized through reaction between Au(CH_3_)L and Au(L)(NTf_2_) {L = phosphines listed above}. For [Au_2_(μ-CH_3_)(XPhos)_2_][NTf_2_] and [Au_2_(μ-CH_3_)(^*t*^BuXPhos)_2_][NTf_2_], solid-state X-ray structures have been elucidated. The rate of ethane formation from [Au_2_(μ-CH_3_)(PMe_2_Ar′)_2_][NTf_2_] increases as the steric bulk of the phosphine substituent Ar′ decreases. Monitoring the rate of ethane elimination reactions by multinuclear NMR spectroscopy provides evidence for a second-order dependence on the gem-digold methyl complexes. Using experimental and computational evidence, it is proposed that the mechanism of C–C coupling likely involves (1) cleavage of [Au_2_(μ-CH_3_)(PMe_2_Ar′)_2_][NTf_2_] to form Au(PR_2_Ar′)(NTf_2_) and Au(CH_3_)(PMe_2_Ar′), (2) phosphine migration from a second equivalent of [Au_2_(μ-CH_3_)(PMe_2_Ar′)_2_][NTf_2_] aided by binding of the Lewis acidic [Au(PMe_2_Ar′)]^+^, formed in step 1, to produce [Au_2_(CH_3_)(PMe_2_Ar′)][NTf_2_] and [Au_2_(PMe_2_Ar′)]^+^, and (3) recombination of [Au_2_(CH_3_)(PMe_2_Ar′)][NTf_2_] and Au(CH_3_)(PMe_2_Ar′) to eliminate ethane.

## Introduction

Organometallic gold precatalysts have been applied to a range of catalytic organic syntheses.^1−9^ Among the Au-catalyzed processes, many involve C–C bond forming reactions as a key step. Thus, the mechanisms of Au-mediated C–C bond formation have been of substantial interest.^10−20^ Also, Au-catalyzed partial oxidation of methane in oleum to form methylbisulfate has been reported.^21,22^ Demonstration, separately, of Au-mediated methane C–H activation^23,24^ and of the ability of molecular Au complexes to mediate C–C bond forming reactions^14−22^ sparked our interest in ethane elimination since combined methane C–H activation and ethane reductive elimination provides a strategy for the oxidative conversion of methane to ethane.^25,26^ Herein, we disclose a mechanistic study of ethane elimination from phosphine-ligated gem-digold^27^ methyl complexes with the general formula [Au_2_(μ-CH_3_)(PMe_2_Ar′)_2_][NTf_2_], [Au_2_(μ-CH_3_)(XPhos)_2_][NTf_2_], and [Au_2_(μ-CH_3_)(^*t*^BuXPhos)_2_][NTf_2_] {Ar = C_6_H_3_-2,6-(C_6_H_3_-2,6-Me)_2_, C_6_H_3_-2,6-(C_6_H_3_-2,4,6-Me)_2_, C_6_H_3_-2,6-(C_6_H_3_-2,6-^*i*^Pr)_2_, or C_6_H_3_-2,6-(C_6_H_3_-2,4,6-^*i*^Pr)_2_; XPhos = 2-dicyclohexylphosphino-2′,4′,6′-triisopropylbiphenyl; ^*t*^BuXPhos = 2-di-*tert*-butylphosphino-2′,4′,6′-triisopropylbiphenyl; NTf_2_ = bis(trifluoromethyl sulfonyl)imide}.

**Scheme 1:**
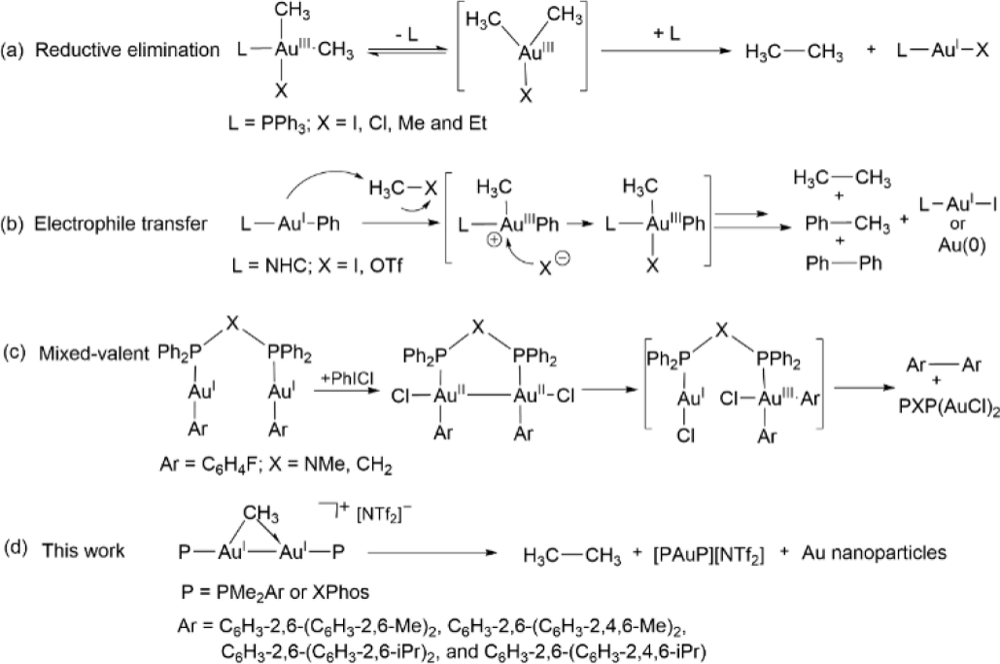
Proposed Pathways of C–C Bond Coupling Reactions Mediated by Molecular Gold Complexes^17,18,31,36^

Proposed mechanisms for Au-mediated C–C bond formation include reductive elimination from Au^III^ intermediates (Scheme 1).^28^ For example, reductive elimination from (R)_2_Au(X)(L) (L = phosphine; R = Me, Et, or ^*n*^Pr; X = anionic ligand such as Cl, OTf, NO_3_, O_2_CCF_3_, or another alkyl ligand) was investigated by Kochi and co-workers, and reductive eliminations from [(Me)_2_Au(L)_2_]^+^ complexes have been reported.^18,29,30^ The proposed mechanism involves initial phosphine dissociation followed by C–C reductive elimination from the three-coordinate R_2_Au^III^X intermediate (Scheme 1a). When R = Me, isotopic labeling studies with (Me)_2_AuX(L) and (CD_3_)_2_Au(X)(L) (L = phosphine) indicate kinetically competitive intermolecular transfer of Me between two Au centers, but these alkyl transfers appear to occur only in nonpolar solvents.^23^ Further, the putative binuclear Au intermediates responsible for alkyl transfer were not directly implicated in the C–C coupling reactions. From the starting complexes (Me)_2_Au(X)(L) (L = phosphine), it was proposed that larger phosphines facilitate ethane reductive elimination.^30^ Alternatively, Kochi has proposed that ethane formation could result from digold alkyl intermediates, but to our knowledge, such reactions were not directly observed.^31,32^ Other examples of ethane formation through bimolecular reductive elimination from M–CH_3_ species include Ni^II^,^33^ Cu^I^,^34^ and Ru^II^.^35^

The formation of C–C bonds from (NHC)Au^I^–R (NHC = *N*-heterocyclic carbene; R = Ph, Me, or *p*-tolyl) occurs upon addition of electrophiles (R′X), such as PhI, MeI, and MeOTf, to form R–R′ as well as homocoupled products R–R and R′–R′ (Scheme 1b).^36−38^ The proposed mechanism involves formal *trans* oxidative addition of the electrophile (R′X) to form an (NHC)Au^III^(R′)(X)(R) intermediate, followed by competitive (a) C–C reductive elimination to form R′–R and (b) intermolecular transfer of RX from a Au(III) intermediate to (NHC)Au^I^–R to yield (NHC)Au^III^(R)_2_(X) followed by C–C reductive elimination to give the homocoupled product R–R. Related to these processes, the addition of F^+^-donors to Au(I) hydrocarbyl compounds also promotes C–C coupling reactions.^39−42^

Mixed-valent gold hydrocarbyl complexes have also been proposed as intermediates responsible for the C—C bond formation.^17^ For example, Toste and co-workers reported a fast biaryl C–C bond reductive elimination from a mixed-valent bimetallic Au^I^/Au^III^ complex [ClAu]PNP[AuCl(C_6_H_4_-4-F)_2_] (PNP = Ph_2_P—N(CH_3_)—PPh_2_) (Scheme 1c).^17^ In this study, the Au(I) complex [Au(C_6_H_4_-4-F)]PNP[Au(C_6_H_4_-4-F)] is oxidized with PhICl_2_ to generate a symmetric bimetallic Au(II) species, [ClAu(C_6_H_4_-4-F)]PNP[Au(C_6_H_4_-4-F)Cl]. The latter isomerizes to a mixed-valent Au^I^/Au^III^ complex, [ClAu]PNP[AuCl(C_6_H_4_-4-F)_2_], which undergoes reductive elimination to form a biaryl product. Similarly, O’Hair and co-workers reported a concerted redox couple mechanism from a reaction between allylic halides (CH_2_ = CHCH_2_X, X = Cl, Br, and I) and a gem-digold(I) compound, [(dppm)_2_Au_2_Ph]^+^ (dppm = bis(diphenylphosphino)methane, (Ph_2_P)_2_CH_2_).^43^ It is hypothesized that the reductive coupling occurs from a Au^I^/Au^III^ complex, [ClAu^I^](dppm)[Au^III^(CH_2_=CHCH_2_)(Ph)].

Germane to these proposed binuclear Au precursors to C—C elimination, several gem-digold intermediates have been reported, including [Au_2_(*σ*,*π*-CH=CHC_3_H_5_)(PPh_3_)_2_][NTf_2_],^44^ [Au_2_(μ-Ph)L_2_][NTf_2_]^45^ (L = PPh_3_ or NHC), and [Au_2_(μ-R)(PMe_2_Ar^Dipp2^)_2_][NTf_2_] (R = CH_3_, CH=CH_2_, C≡CH, Ar^Dipp2^=C_6_H_3_-2,6-(C_6_H_3_-2,6-^*i*^Pr)_2_).^46^ The thermal stabilities of phosphine-ligated gem-digold hydrocarbyl complexes have been reported to depend on the steric properties of the ancillary ligands.^46^ Other related examples, including [Au_2_(μ-vinyl^cypr^)(PPh_3_)_2_][NTf_2_]^14,15,44^ and [Au_2_(μ-vinyl^cypr^)(PPh_3_)_2_][NTf_2_], readily decompose to the corresponding diene, [Au(PPh_3_)_2_][NTf_2_], and colloidal gold byproducts. Nonetheless, a mechanistic understanding of these C—C coupling processes and, in general, of C—C formation from Au^I^ complexes is lacking.

**Figure 1:**
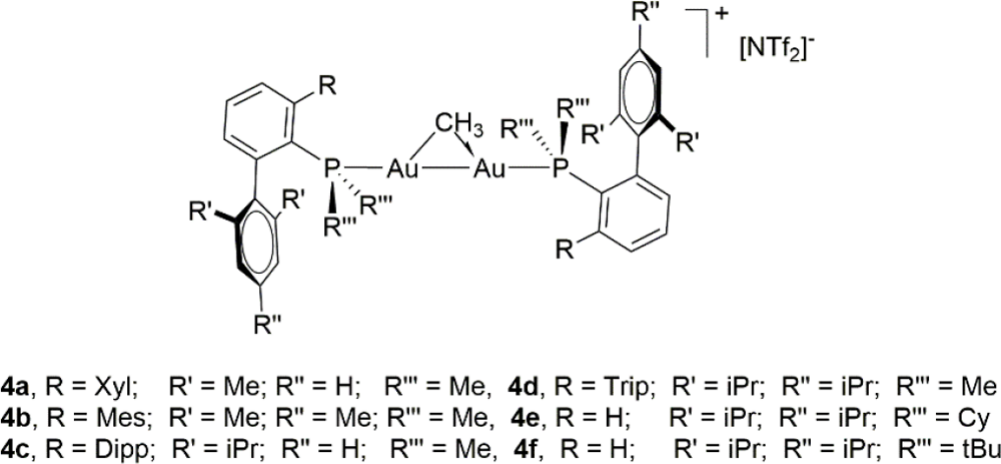
Phosphine-ligated gem-digold methyl complexes with the general formula [Au_2_(μ-CH_3_)(PR_2_Ar′)_2_][NTf_2_] investigated in this work (Xyl = 2,6-C_6_H_3_-Me_2_; Mes = 2,4,6-C_6_H_2_-Me_3_; Dipp = 2,6-C_6_H_3_-^*i*^Pr_2_; Tripp = 2,4,6-C_6_H_2_-^*i*^Pr_3_).

Herein, we explore the formation of ethane from one of the simplest possible gold-based systems, namely, Au(CH_3_)(PPh_3_). To enable reliable mechanistic investigations, we extended our preliminary observations on triphenylphosphine-ligated systems to bulkier terphenyl and biaryl phosphines that provide kinetic stabilization of key digold intermediates. In particular, we have focused on C–C coupling reactions from gem-digold methyl complexes with a general formula [Au_2_(μ-CH_3_)(PMe_2_Ar′)_2_][NTf_2_] and [Au_2_(μ-CH_3_)(XPhos)_2_][NTf_2_] (Figure 1). We studied the impact of the phosphine ligand on the stability of digold complexes, especially the influence on ethane elimination.

## Results and Discussion

### Synthesis of Neutral Gold Complexes Based on Terphenyl and Biaryl Phosphines

**Scheme 2:**
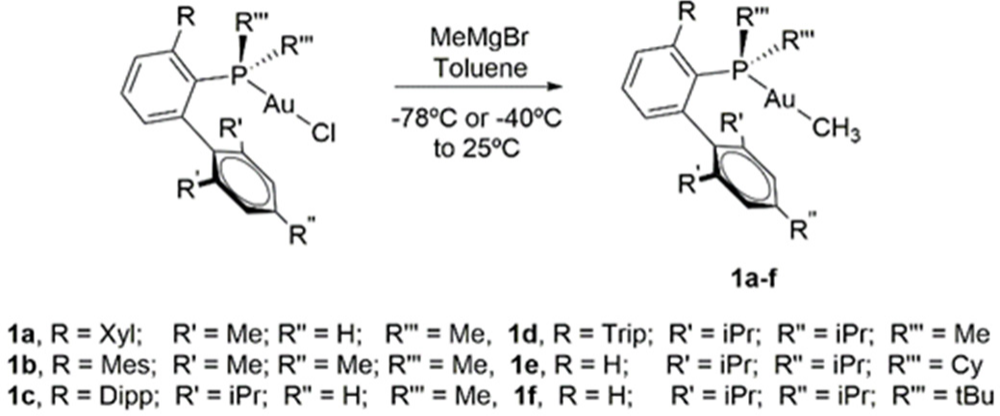
Synthesis of Phosphine-Ligated Gold Methyl Compounds with Terphenyl Phosphines (**1a**–**1d**) and Buchwald Phosphines (**1e** and **1f**)

**Figure 2:**
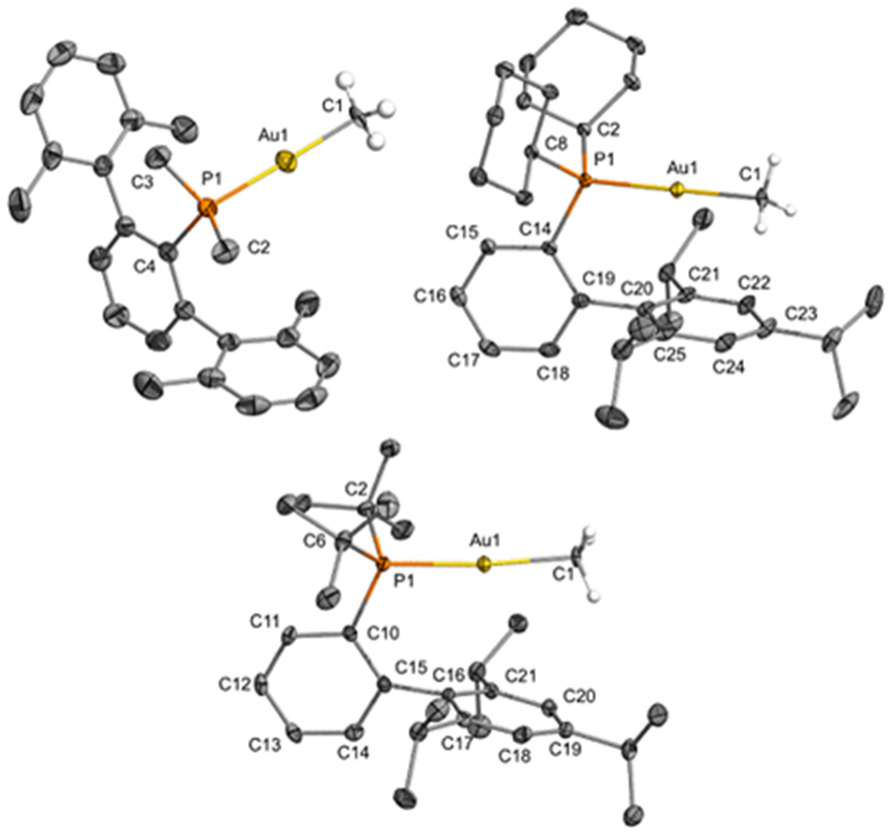
ORTEPs of Au(CH_3_)(PMe_2_Ar^Xyl2^) (**1a**), Au(CH_3_)(XPhos)(**1e**), and Au(CH_3_)(^*t*^BuXPhos) (**1f**) represented at 50% probability. (For **1f**, one of the two chemically equivalent, but crystallographically distinct, structures is shown. For the second structure, see the Supporting Information.) Hydrogen atoms on the phosphine ligands are omitted for clarity. Selected bond lengths (Å): **1a**, Au1–C1 = 2.123(2); P1–C2 = 1.825(3); P1–C3 = 1.823(3); P1–C4 = 1.852(2); Au1–P1 = 2.2900(7). **1e**, Au1–C1 = 2.1146(14); Au1–C20 = 3.1748(17); Au1–C25 = 3.2510(18); Au1–C21 = 3.5023(18); Au–arene (arene ring centroid) = 3.2659(10); Au1–P1 = 2.2917(4). **1f**, Au1–C1 = 2.096(4); Au1–C16 = 3.180(4); Au1–C17 = 3.551(4); Au1–C21 = 3.409(4); Au–arene (benzene ring centroid) = 3.449(2); Au1–P1 = 2.3007(11). Selected bond angles (deg): **1a**, P1–Au1–C1 = 178.97(8); C2–P1–Au1 = 112.8(1); C3–P1–Au1 = 111.95(10); C4–P1–Au1 = 113.14(8). **1e**, P1–Au1–C1 = 179.57(4); C14–P1–Au1 = 117.53(5). **1f**, C1–Au1–P1 = 172.80(12); C10–P1–Au1 = 115.25(13).

Gold complexes with terphenyl phosphine (complexes **1a**–**1d** in Scheme 2) and with biaryl “Buchwald phosphine” ligands (**1e** and **1f**) were synthesized by methylation of Au(I) chloride precursors with MeMgX (X = Cl or Br) in 60–80% isolated yields.^47^ Formation of the new Au–C bonds is evidenced by the appearance of ^1^H NMR resonances in the range from 0.08 to 0.45 ppm with associated ^13^C{^1^H} signals at 3.4 to 8.3 ppm (^2^*J*_CP_ ≈ 100 Hz). Single crystals of **1a**, **1e**, and **1f** were obtained by slow evaporation from a mixture of pentane and diethyl ether or pentane and dichloromethane solution from 5 to −25 °C (Figure 2). The solid-state structures of complexes **1e** and **1f** show a weak κ^1^ type interaction (localized Au···π(arene) contact)^48−50^ between the Au(I) center and the *ipso* carbon of the arenes (C20, **1e**; C16, **1f**) with bond distances of 3.1748(17) and 3.180(4) Å, respectively. The distances between Au centers and arene ring centroids are 3.2659(10) Å (**1e**) and 3.449(2) Å (**1f**), also indicative of intramolecular Au···π(arene) interactions.^50,51^ Structure **1a** does not exhibit this type of contact, in agreement with the preferred geometry adopted by the smaller phosphines of the terphenyl series.^52^ The Au–CH_3_ bond distances are 2.123(2) Å (**1a**), 2.1146(14) Å (**1e**), and 2.096(4) Å (**1f**).

**Figure 3:**
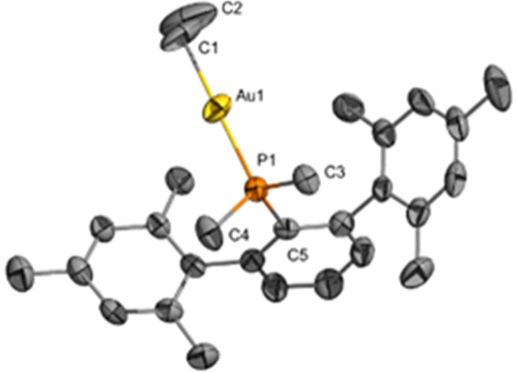
ORTEP of Au(C_2_H_5_)(PMe_2_Ar^Mes2^) (**2a**) at 50% probability (one of the two crystallographically distinct structures, the other one being Au(C_2_H_5_)(PMe_2_Ar^Xyl2^), see Figure S1). Hydrogen atoms on the phosphine ligands are omitted for clarity. Selected bond lengths (Å): Au1–C1 = 2.079(8); C1–C2 = 1.411(15). Selected bond angles (deg): C1–Au1–P1 = 179.6(3); Au1–C1–C2 = 115.0(7); C3–P1–Au1 = 111.5(3); C5–P1–Au1 = 115.63(19); C4–P1–Au1 = 112.2(3).

**Figure 4:**
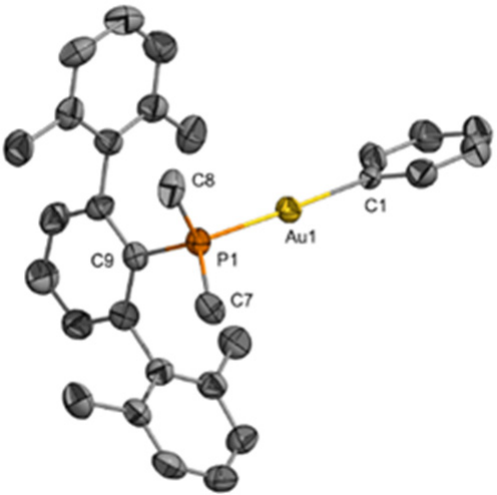
ORTEP of Au(C_6_H_5_)(PMe_2_Ar^Xyl2^) (**3a**) at 50% probability. Hydrogen atoms on the phosphine ligands are omitted for clarity. Selected bond lengths(Å): Au1–C1 = 2.089(7); Au1–P1 = 2.302(2). Selected bond angles (deg): C1–Au1–P1 = 177.7(2); C8–P1–Au1 = 110.4(3); C7–P1–Au1 = 109.9(3); C9–P1–Au1 = 117.5(2).

Terminal ethyl and phenyl complexes Au(C_2_H_5_)(PMe_2_Ar^Xyl2^) (**2a**) and Au(C_6_H_5_)(PMe_2_Ar^Xyl2^) (**3a**) were synthesized with the aim of exploring the possibility of C–C bond heterocoupling with different hydrocarbyl substituents bound to gold (see below). These compounds were prepared by a similar procedure to their methyl analogues and characterized by spectroscopic techniques and single-crystal X-ray diffraction (Figures 3 and 4). The σ Au–C bond distances are 2.079(8) Å (**2a**) and 2.087(7) (**3a**) Å, which are similar to neutral Au–CH_3_ bond distances discussed above. Complex **2a** cocrystallizes in a 1:1 ratio with a molecule of Au(C_2_H_5_)(PMe_2_Ar^Mes2^) (**2b**, see Figure S1), whose geometric parameters are comparable to those of **2a**. This is due to the fact that the crystals were obtained from a phosphine exchange experiment between **2a** and free PMe_2_Ar^Mes2^ that was conducted as part of our mechanistic investigations (see below, Figure S1). The bond distance between C1 and C2 in the Au-ethyl fragment of **2a** is 1.411(15) Å, lying between the carbon–carbon lengths of ethylene (1.34 Å) and ethane (1.54 Å). The electrophilic nature of gold may enhance the C–C bond strength and thus shorten bond length compared to a typical C–C single bond. The structure of complex **3a** is similar to those of compounds **1** and **2a** and does not require further discussion.

### Ethane Elimination from Au(CH_3_)(PPh_3_)

**Scheme 3:**
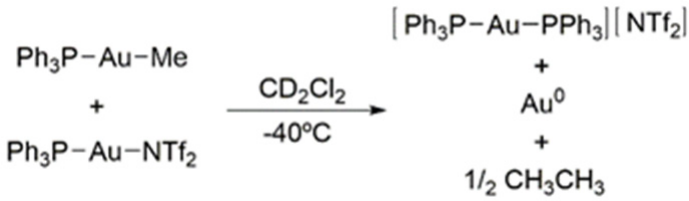
Ethane Elimination from Au(CH_3_)(PPh_3_) in the Presence of 1 equiv of Au(PPh_3_)(NTf_2_)

**Figure 5:**
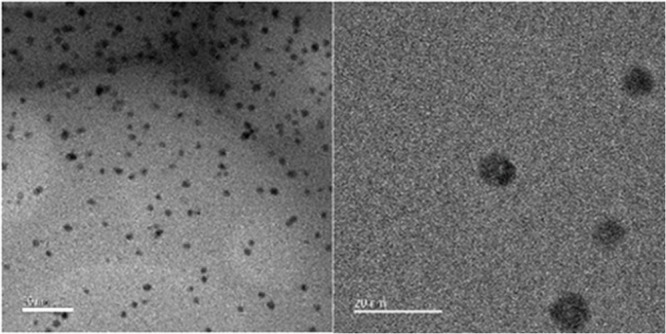
Transmission electron microscopy (TEM) analysis of the insoluble Au^0^ particles produced during ethane evolution in Scheme 3.

For the sake of simplicity and considering the widespread utilization of PPh_3_-based gold complexes, we commenced our studies by exploring ethane elimination from Au(CH_3_)(PPh_3_). This compound is stable at moderate temperatures as heating at 40 °C caused no apparent alteration when monitoring by ^1^H and ^31^P{^1^H} NMR spectroscopy, and no ethane formation was detected. However, in the presence of 1 equiv of Au(PPh_3_)(NTf_2_), Au(CH_3_)(PPh_3_) evolves ethane immediately at room temperature with complete consumption of Au(CH_3_)(PPh_3_) by the time of placing the sample in the NMR probe (<10 min; Scheme 3). The release of ethane is accompanied by clean formation of the homoleptic bisphosphine complex [Au(PPh_3_)_2_][NTf_2_], along with Au(0), as evinced by the formation of black insoluble material. The nature of this solid was interrogated by transmission electron microscopy (TEM) analysis (Figure 5). When a 1:1 molar mixture of Au(PPh_3_)(NTf_2_) and Au(CH_3_)(PPh_3_) was dissolved in dichloromethane at −70 °C, ethane formation was detected immediately by ^1^H NMR spectroscopy (Figure S9). Variable temperature ^1^H and ^31^P{^1^H} NMR analysis from −70 to 25 °C revealed the formation of an intermediate species characterized by a broad ^1^H NMR resonance at 1.6 ppm associated with a ^31^P{^1^H} NMR resonance at 37.5 ppm, which we attribute to the corresponding gem-digold methyl species [Au_2_(μ-CH_3_)(PPh_3_)_2_][NTf_2_] (Figure S8).^46^ However, this compound is only detectable at temperatures below −40 °C, and it rapidly evolves to the final products above this temperature.

Though the transient nature of [Au_2_(μ-CH_3_)(PPh_3_)_2_][NTf_2_] prevented us from exploring its role in further detail, our initial kinetic investigations using 1 equiv of the related Au(PPh_3_)(NO_3_) revealed a second-order dependence on neutral Au(CH_3_)(PPh_3_) for ethane elimination (Figure S6). Nonetheless, we could carry out these studies with related methyl complexes based on biaryl and terphenyl phosphines, as discussed in the following sections. Since we observed the formation of gold nanoparticles during ethane elimination, we decided to probe for a possible catalytic role of Au nanoparticles in the C–C coupling reaction, particularly considering their catalytic role in related processes.^53−55^ However, using independently prepared gold nanoparticles (i.e., Au/TiO_2_ and Au/Fe_2_O_3_) as catalysts did not promote methyl C–C coupling at a comparable rate (*t*_1/2_ ≈ 1 day at 25 °C). Thus, it seems unlikely that Au nanoparticles play a catalytic role in the formation of ethane. In addition, we tested for the possibility of a radical-mediated pathway. To do so, we combined equimolar amounts of Au(CH_3_)(PPh_3_) and [Au(PPh_3_)][NTf_2_] in the presence of excess toluene (10 equiv) as a radical probe. Under these conditions, the formation of CH_3_• radicals should be quenched by toluene by means of hydrogen atom abstraction from the benzylic position.^56^ This process would have released methane, which was not observed during our experiments, thus favoring the likelihood of a nonradical route.

### Synthesis of Cationic gem-Digold Methyl Complexes

**Scheme 4:**
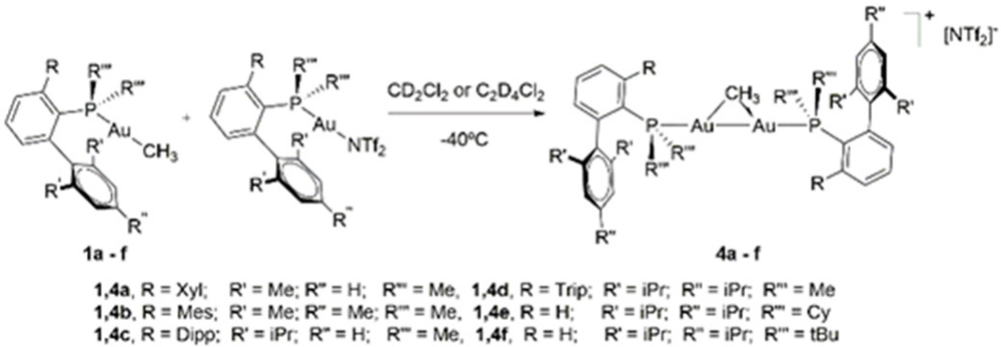
General Synthesis of the gem-Digold Methyl Complexes with Terphenyl Phosphines (**4a**–**4d**) and Buchwald Phosphine-Ligated gem-Digold Methyl Complexes (**4e** and **4f**)

To probe if gem-digold methyl complexes are relevant intermediates during C–C coupling reactions, bulky terphenyl and Buchwald phosphines were used. Some of us have recently demonstrated that gem-digold methyl species are kinetically stabilized by large phosphine substituents,^46^ which should facilitate kinetic investigations. Indeed, using the aforementioned bulky phosphines enabled the isolation and characterization of various uncommon gem-digold methyl complexes of type [Au_2_(μ-CH_3_)(PMe_2_Ar′)_2_][NTf_2_] (**4a**–**4d**). These were synthesized in high yields by mixing a 1:1 molar ratio of a Au(I) methyl complex Au(CH_3_)(PMe_2_Ar′) and the corresponding Au(I) bis(trifluoromethyl sulfonyl)imide (Scheme 4). Alternatively, the addition of 0.5 equiv of [Ph_3_C][B(C_6_F_5_)_4_] to neutral Au(I) methyl complexes Au(CH_3_)(PR_2_Ar′) leads to the same gem-digold species in comparable yields.

**Figure 6:**
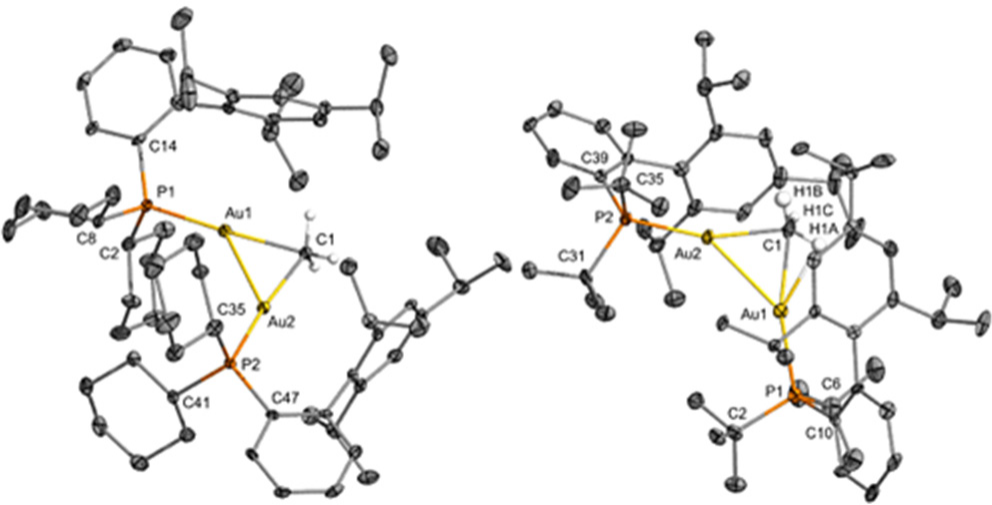
ORTEPs of [Au_2_(*μ-*CH_3_)(XPhos)_2_][NTf_2_] (**4e**) and [(Au)_2_(μ-CH_3_)(^*t*^BuXPhos)_2_][NTf_2_] (**4f**) at 50% probability (for **4e**, only one of the three chemically equivalent, but crystallographically distinct, structures is represented). Hydrogen atoms on the phosphine ligands are omitted for clarity. Selected bond lengths (Å): **4e**, Au1–C1 = 2.221(5); Au2–C1 = 2.235(5); Au1–Au2 = 2.7466(4); Au1–P1 = 2.2637(12); Au2–P2 = 2.2662(13). **4f**, Au1–C1 = 2.204(9); Au2–C1 = 2.207(8); Au1–Au2 = 2.7763(7); Au1–P1 = 2.285(2); Au1–P2 = 2.2798(18). Selected bond angles (deg): **4e**, C1–Au1–P1 = 168.41(14); C1–Au2–P2 = 172.39(13); Au1–C1–Au2 = 76.11(16); C1–Au1–Au2 = 52.17(13); C1–Au2–Au1 = 51.72(12). **4f**, C1–Au1–P1 = 162.9(2); C1–Au2–P2 = 160.9(2); Au1–C1–Au2 = 78.0(3); C1–Au1–Au2 = 51.0 (2); C1–Au2–Au1 = 50.9 (2).

Compounds **4a**–**4f** were characterized by multinuclear NMR spectroscopy, and their purity was confirmed by microanalysis. Distinctive ^1^H NMR signals due to the methyl group, which are slightly shifted to higher frequencies (ca. 0.5–1.2 ppm) compared to their corresponding neutral precursors (**1a**–**1f**), are consistent with the formation of the gem-digold complexes. The presence of the bridging methyl ligand is further confirmed by ^13^C{^1^H} NMR resonances shifted to lower frequencies by approximately 5 ppm compared to the parent compounds **1a**–**1f** and characterized by a drastically reduced scalar-coupling to ^31^P (ca. 50 Hz; cf. ∼100 Hz for **1a**–**1f**). The compounds [Au_2_(*μ-*CH_3_)(XPhos)_2_][NTf_2_] (**4e**) and [(Au)_2_(μ-CH_3_)(^*t*^BuXPhos)_2_][NTf_2_] (**4f**) were additionally authenticated by single-crystal X-ray diffraction (Figure 6; Table 1). The gold methyl bond distances in **4e** and **4f** are ∼0.1 Å longer than in their corresponding neutral methyl complexes **1e** and **1f**. A characteristic Au–arene interaction is discernible for the two structures. While the structure of **4f** exhibits a slightly shortened Au–arene distance (3.390(3) Å on average) than its neutral complex **1f** (3.449(2) Å), compound **4e** (3.432(2) Å on average) presents an apparently weaker Au–arene interaction than its neutral gold compound **1e** (3.2659(1) Å). The presence of intense aurophilic interactions^57,58^ is evinced by Au···Au distances in complexes **4e** and **4f** of 2.7466(6) and 2.7763(7) Å, respectively. These Au···Au distances are slightly longer than those reported for the related **4c** (2.7120(8) Å) and ∼0.1 Å shorter than the Au–Au distance in metallic gold (2.878 Å).

**Table 1:** Summary of Selected Bond Distances of the gem-Digold Methyl Complexes

gem-digold methyl complexes	Au–arene^a^ (Å)	Au–Au (Å)	Au–*ipso* carbon of arene (Å)	Au–CH_3_ (Å)
[Au_2_(μ-CH_3_)(PMe_2_Ar^Dipp2^)_2_][BAr_F_]^34^ (**4c**)	3.259(3)	2.7120(8)	3.027(3)	2.210(5)
	3.321(3)	2.7120(8)	3.102(3)	2.227(4)
[Au_2_(*μ-*CH_3_)(XPhos)_2_][NTf_2_]^b^ (**4e**)	3.400(2)	2.7330(4)	3.093(5)	2.215(5)
	3.465(2)	2.7330(4)	3.185(5)	2.238(5)
[(Au)_2_(μ-CH_3_)(^*t*^BuXPhos)_2_][NTf_2_] (**4f**)	3.366(3)	2.7765 (5)	3.082(8)	2.207(8)
	3.413(3)	2.7765 (5)	3.406(8)	2.204(9)

a Distance from Au to the centroid of the arene rings.

b Average over three independent molecules present in the asymmetric unit.

### Ethane Elimination from gem-Digold Methyl Complexes

**Scheme 5:**
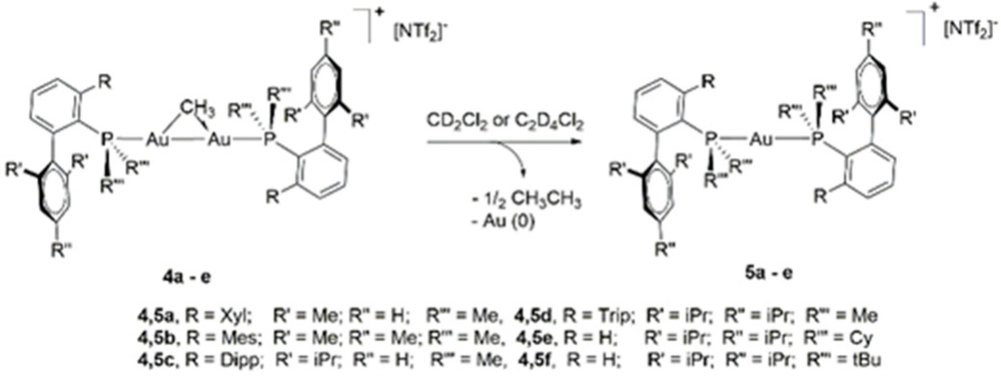
Thermal Decomposition of Terphenyl and Biaryl Phosphine Methyl-Bridged Digold Complexes (**4a**–**4e**) to Gold Bisphosphine (**5a**–**5e**)

As anticipated, the stability of gem-digold methyl complexes largely depends on the steric shielding provided by the phosphine ligand. Thus, the compound [Au_2_(μ-CH_3_)(PMe_2_Ar^Xyl2^)_2_][NTf_2_] (**4a**) is only stable in dichloromethane solution at −30 °C or below. Above −20 °C, **4a** cleanly converts into [Au(PMe_2_Ar^Xyl2^)_2_][NTf_2_] (**5a**), metallic gold, and ethane (Scheme 5). Complex [Au_2_(μ-CH_3_)(PMe_2_Ar^Mes2^)_2_][NTf_2_] (**4b**) reacts in a similar way, whereas bulkier phosphines provide enhanced stability. As such, compounds [Au_2_(μ-CH_3_)(PMe_2_Ar^Dipp2^)_2_][NTf_2_] (**4c**) and [Au_2_(μ-CH_3_)(PMe_2_Ar^Tripp2^)_2_][NTf_2_] (**4d**), in which the methyl substituents in the lateral aryl rings of the terphenyl moiety have been substituted by *iso*-propyl groups, are fairly stable at room temperature, while complexes **4e** and **4f** remain unaltered for hours even at temperatures up to 80 °C. Thus, the investigated Buchwald phosphines confer enhanced stability to gem-digold methyl species compared to terphenyl-based ligands, most likely as a result of the increased steric shielding provided by the cyclohexyl and *tert*-butyl groups directly bound to the phosphorus center in close proximity to the gold nuclei.

**Figure 7:**
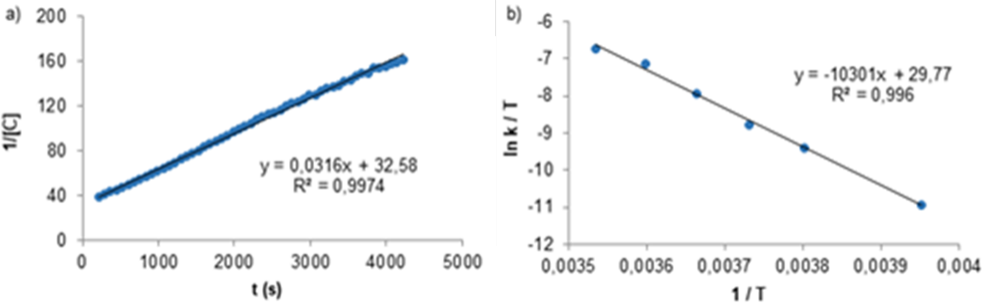
(a) Second-order kinetic representation for the consumption of **4a** at −5 °C in CD_2_Cl_2_. (b) Eyring plot for ethane formation from gem-digold methyl [Au_2_(μ-CH_3_)(PMe_2_Ar^Xyl2^)_2_][NTf_2_] (**4a**).

Overall, these observations indicate that kinetic analysis by ^1^H and ^31^P{^1^H} NMR spectroscopy monitoring is facilitated by larger phosphine ligands compared to PPh_3_. For instance, heating complex **4e** in dichloroethane at 90 °C enabled us to monitor by NMR spectroscopy its evolution to [Au(XPhos)_2_][NTf_2_] (**5e**) with concomitant release of ethane and formation of Au(0) (Figure S5). The thermolysis of **4e** follows a second-order dependence on the digold complex with *k*_obs_ = 5.2(1) × 10^–4^ M^–1^ s^–1^ at 90 °C (Table 2), as previously observed for the PPh_3_-based system. In the case of the more hindered compound **4f**, this reaction does not take place at 100 °C, and intractable digold decomposition occurs at temperatures above 100 °C where the formation of methane, instead of ethane, was observed (Figure S14). This finding indicates that C–C coupling is likely not viable in the most sterically constrained digold system studied herein. This seems to be consistent with a second-order dependence on digold complex concentration during ethane formation, which might imply the need for more than two gold nuclei in close proximity along the reaction coordinate (see below for additional discussion). Similar to complex **4e**, ethane elimination from terphenyl-ligated gem-digold methyl complexes follows a second order dependence on **4a**–**4d** (Figure 7a; see the Supporting Information). Kinetic studies provide rates for ethane elimination from the more sterically hindered **4c** and **4d** of *k*_obs_ = 4.8(3) × 10^–3^ and 2.0(1) × 10^–2^ M^–1^ s^–1^ at 50 °C, respectively. In contrast, the rates of ethane elimination from **4a** and **4b** had to be analyzed at lower temperatures (0 °C), resulting in rates of *k*_obs_ = 9.8(3) × 10^–2^ and 4.9(1) × 10^–1^ M^–1^ s^–1^ at 0 °C, respectively. The corresponding half-life (*t*_1/2_) values associated with these kinetic parameters at the working temperatures are approximately 260 (**4a**, 0 °C), 800 (**4b**, 0 °C), 5600 (**4c**, 50 °C), and 13 000 (**4d**, 50 °C) s.

**Table 2:** Summary of Kinetic Data for Ethane Elimination from gem-Digold Complexes **4a**–**4e**

compound	*T* (°C)	*k* (M^–1^ s^–1^)	Δ*G*^⧧^ (kcal/mol)
[Au_2_(μ-CH_3_)(PMe_2_Ar^Xyl2^)_2_][NTf_2_] (**4a**)	0	9.8(3) × 10^–2^	17.2(1)
[Au_2_(μ-CH_3_)(PMe_2_Ar^Mes2^)_2_][NTf_2_] (**4b**)	0	4.9(1) × 10^–2^	17.6(1)
[Au_2_(μ-CH_3_)(PMe_2_Ar^Dipp2^)_2_][NTf_2_] (**4c**)	50	4.8(3) × 10^–3^	22.4(5)
[Au_2_(μ-CH_3_)(PMe_2_Ar^Tipp2^)_2_][NTf_2_] (**4d**)	50	2.0(1) × 10^–3^	22.9(4)
[Au_2_(μ-CH_3_)(XPhos)_2_][NTf_2_] (**4e**)	90	5.2(1) × 10^–4^	26.4(3)
[Au_2_(μ-CH_3_)(^*t*^BuXPhos)_2_][NTf_2_] (**4f**)	100^a^	N.A.	N.A.

a Methane formation observed instead; N.A. (not available).

Table 2 collects the corresponding activation barriers for C–C coupling from the methyl-bridged complexes **4a**–**4e**, which range from 17.2 kcal/mol at 0 °C for **4a** to 26.4 kcal/mol at 90 °C for **4e**. To complete these studies, we monitored the evolution of ethane from the gem-digold complex **4a** in the temperature interval from −20 to 10 °C. An Eyring analysis provided activation parameters of Δ*H*^⧧^ = 20.5 ± 1.3 kcal/mol and Δ*S*^⧧^ = 11.9 ± 4.8 e.u. (Figure 7b), which correspond to Δ*G*_298_^⧧^ = 16.9 ± 2.7 kcal/mol.

**Scheme 6:**
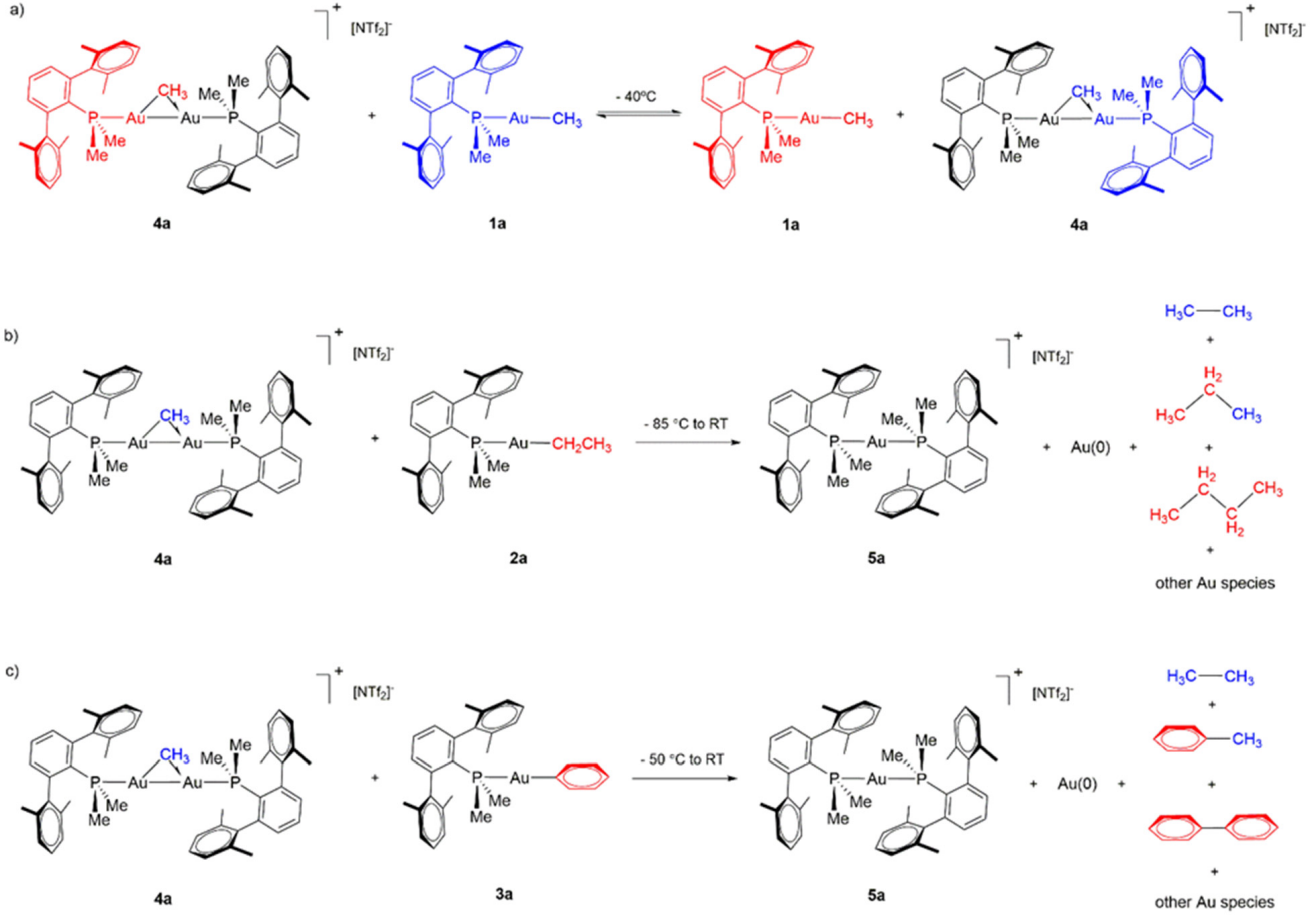
(a) Dynamic Me/Me Exchange Equilibrium between [Au_2_(μ-CH_3_)(PMe_2_Ar^Xyl2^)_2_][NTf_2_] (**4a**) and Au(CH_3_)(PMe_2_Ar^Xyl2^) (**1a**) Species at −40°C; (b) C–C Coupling and Product Distribution in the Reaction between Au(C_2_H_5_)(PMe_2_Ar^Xyl2^) (**2a**) and [Au_2_(μ-CH_3_)(PMe_2_Ar^Xyl2^)_2_][NTf_2_] (**4a**); and (c) C–C Coupling and Product Distribution in the Reaction between Au(C_6_H_5_)(PMe_2_Ar^Xyl2^) (**3a**) and [Au_2_(μ-CH_3_)(PMe_2_Ar^Xyl2^)_2_][NTf_2_] (**4a**)

To obtain a deeper insight into the nature of the Au species involved in C–C coupling processes, we first considered whether dissociation of complexes **4** into their monometallic components,^59^ namely, neutral methyl compounds **1** and triflimide species of type Au(PR_2_Ar′)(NTf_2_), might be relevant. To check the viability of such equilibria, we explored exchange processes of the methyl bridge in compound **4a**. In a first experiment, we examined the exchange between **1a** and **4a** at variable temperatures. For experimental convenience, we accessed an equimolar mixture of both species by adding 0.33 equiv of [Ph_3_C][B(C_6_F_5_)_4_] to **1a** at −40 °C. Under these conditions, one-third of the neutral methyl compound is transformed by methyl abstraction into a cationic gold species that is immediately trapped by unreacted **1a** to provide gem-digold **4a**. Variable temperature ^1^H and ^31^P{^1^H} NMR spectroscopy analysis revealed dynamic behavior in solution (Figure S10), which we attribute to the exchange equilibrium depicted in Scheme 6a. It was possible to identify **4a** by a ^31^P{^1^H} NMR resonance at 0.1 ppm recorded at −85 °C, whereas a broad signal at 21.1 ppm was assigned to **1a**. These signals coalesce at approximately −40 °C, while the major component when reaching 25 °C is the homoleptic bisphosphine compound **5a** that accompanies ethane formation. We further investigated this dynamic behavior by DFT methods (see the Supporting Information for details). Calculations indicate that dissociation of the dinuclear species [Au_2_(μ-CH_3_)(PMe_2_Ar^Xyl2^)_2_][NTf_2_] (**4a**) into the corresponding fragments, Au(CH_3_)(PMe_2_Ar^Xyl2^) (**1a**) and Au(PMe_2_Ar^Xyl2^)(NTf_2_), is only slightly endergonic (Δ*G* = +0.5 kcal/mol), in agreement with our experimental results. The kinetic profile of ethane evolution in these equimolar mixtures is identical, within the experimental error, to that of pure **4a**.

This suggests that, even if carbon–carbon coupling takes place from a trimetallic species involving the participation of compounds **1**, the required dissociation of gem-digold methyl compounds **4** into compounds **1** and [Au(PR_2_Ar′)]^+^ is not likely kinetically relevant.

Substituting methyl compound **1a** by its related ethyl (**2a**) and phenyl (**3a**) derivatives showed the formation of cross-coupling products (Scheme 6b,c). In the case of **2a**, the formation of propane and butane was apparent by ^1^H NMR spectroscopy, while in the reaction between **4a** and **3a** the formation of ethane, biphenyl, and toluene was detected in comparable amounts. GC-MS analysis of solution and gas headspace provided further evidence for cross coupling, since variable amounts of ethane, propane, and butane were measured from the reaction between **2a** and **4a** (Figure S21). In both cases, the main homogeneous gold-containing species when reaching room temperature is **5a**.

**Figure 8:**
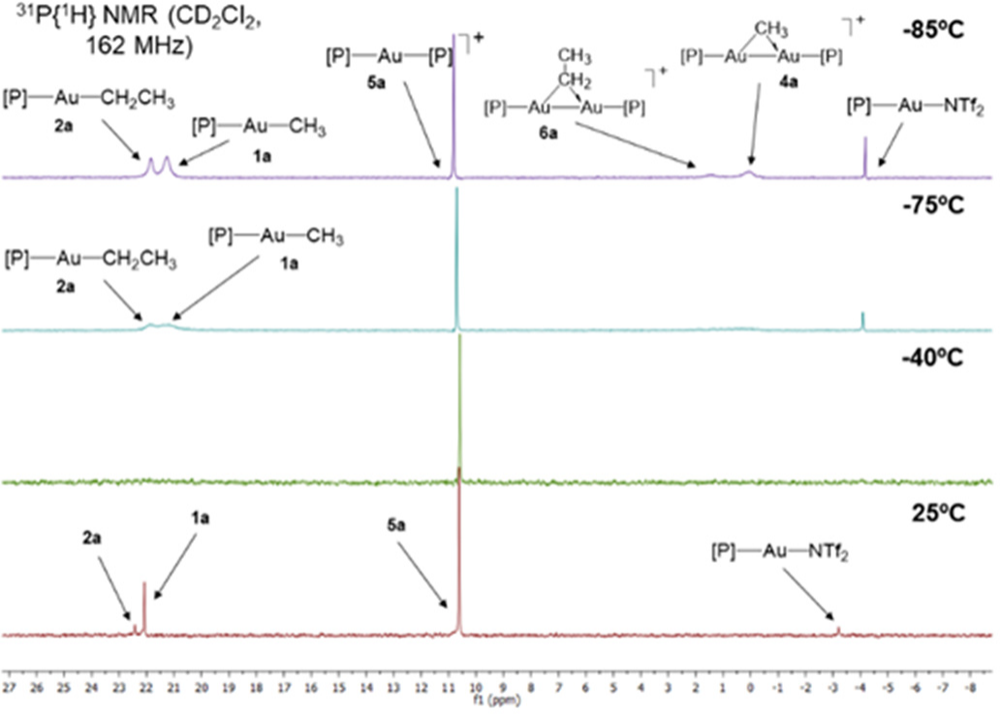
Variable temperature of exchange processes between Au(C_2_H_5_)(PMe_2_Ar^Xyl2^) (**2a**) and [Au_2_(μ-CH_3_)(PMe_2_Ar^Xyl2^)_2_][NTf_2_] (**4a**) monitored by ^31^P{^1^H} NMR spectroscopy.

To gather more information on the exchange between bridging and terminal hydrocarbyl substituents present in gem-digold and neutral compounds, we examined the reaction depicted in Scheme 6b at variable temperatures (Figure 8). A solid mixture of **2a** and **4a** in equimolar amounts was dissolved in CD_2_Cl_2_ at −40 °C to allow the exchange to take place and then cooled to −85 °C. At the latter temperature, the exchange process is halted, and a variety of gold-containing products are identified by ^31^P{^1^H} NMR. These include the neutral hydrocarbyl compounds **1a** and **2a** and their corresponding gem-digold species **4a** and [Au_2_(μ-CH_2_CH_3_)(PMe_2_Ar^Xyl2^)_2_] (**6a**), whose broad resonances were recorded at 21.2, 21.9, 0.1, and 1.4 ppm, respectively. Also, sharp signals due to Au(PMe_2_Ar^Xyl2^)(NTf_2_) and [Au(PMe_2_Ar^Xyl2^)_2_][NTf_2_] (**5a**) were identified at −4.2 and 10.8 ppm, respectively. The latter likely results from local solution warm-up during sample handling. Increasing the temperature to −40 °C results in coalescence of all prior resonances except for that of **5a**, which is clearly not involved in the exchange process. Further raising the temperature to 25 °C leads to full consumption of gold precursors and quantitative formation of bisphosphine compound **5a** along with the appearance of solid Au(0). Similarly, rapid exchange between **4a** and **3a** is evinced by immediate conversion of an equimolar mixture of those compounds into **1a** and [Au_2_(μ-C_6_H_5_)(PMe_2_Ar^Xyl2^)_2_] (**7a**) (Figure S11).

**Scheme 7:**
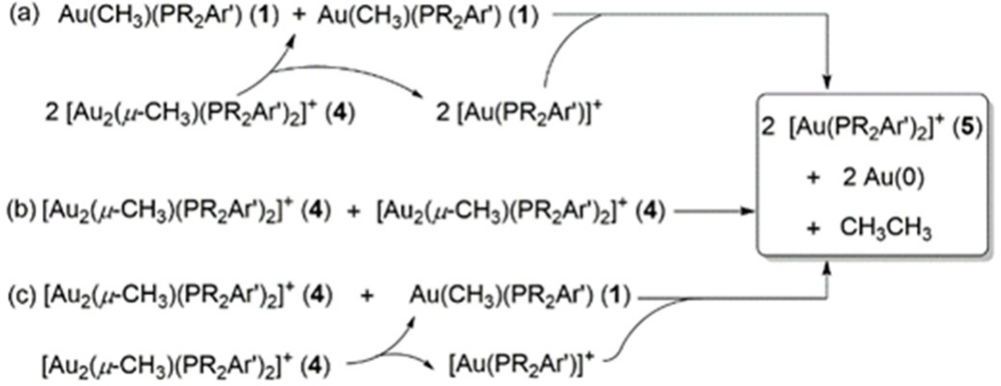
Potential Routes for Ethane Evolution with Regards to the Gold Coupling Partners

Having in mind that the above dynamic behavior reveals the presence of compounds **1**, **4**, and Au(PR_2_Ar′)(NTf_2_) in solution, and also considering the fact that ethane evolution follows a second-order dependence on bridging methyl complexes **4**, we considered three possible routes (Scheme 7). In the first, reductive coupling from two neutral gold methyl compounds of type **1** may take place, similar to prior work by Kochi and co-workers (Scheme 7a).^31,32^ However, it is important to highlight two distinctive features of our studies that contrast with those prior reports. First, reductive coupling from Au(CH_3_)(PPh_3_) only occurred at high temperatures (∼100 °C), while C–C bond formation from bridging digold complexes **4** is more facile. In fact, the C–C coupling reaction readily proceeds at temperatures as low as −60 °C in the case of the PPh_3_-based system (Figures S8 and S9). Second, whereas a first-order dependence on gold was demonstrated for reductive coupling from Au(CH_3_)(PPh_3_),^31,32^ with phosphine dissociation toward “AuMe” as the rate-determining step, we have determined a second-order dependence on digold compounds **4** during ethane evolution. These observations suggest different operating mechanisms in the two cases, a notion that is further supported by DFT methods based on the PMe_2_Ar^Xyl2^ system. In agreement with Kochi’s findings, the computed reaction free energy for phosphine dissociation at **1a** is +32.1 kcal/mol, much higher than the experimentally determined activation free energy for the overall process (Δ*G*_298_^⧧^ = 16.9 ± 2.7 kcal/mol, see above). Phosphine dissociation from **4a** to yield [Au_2_(μ-CH_3_)(PMe_2_Ar^Xyl2^)][NTf_2_], where the metal–metal and metal–arene interactions could stabilize the unsaturated gold center, presented a similarly high value [+31.1 kcal/mol; Figure S20 (including a molecule of CH_2_Cl_2_ in the calculation to compensate the unsaturation of the metal center results in higher reaction free energies: +33.3 and +42.6 kcal/mol for phosphine dissociation from **1a** and **4a**, respectively)]. As anticipated, these data confirm a dissimilar C–C coupling mechanism for compounds **4** compared to that exhibited by monometallic gold-alkyl species.

An alternative route consists of two gem-digold methyl fragments **4** approaching to facilitate the C–C coupling event (Scheme 7b). However, a third pathway to consider, in view of the Coulombic repulsion derived from the approach of two cationic species in route b, is the reaction between **4** and its corresponding neutral methyl species **1** formed by dissociation of a second molecule of **4** into their monometallic fragments (Scheme 7c). First, we explored computationally the direct coupling of methyl groups between two molecules of **4a** (route a) as well as between **4a** and **1a** (route b) by relaxed potential energy scans. These studies indicate that those pathways are unfeasible, both in the singlet and triplet state. We also evaluated the possibility of accessing the hypothetical Au(III) species [Au(CH_3_)_2_(PMe_2_Ar^Xyl2^)][NTf_2_] from the above routes, since reductive coupling of ethane with such a complex should be accessible.^18,29,30^ In fact, we found a feasible barrier (+16.1 kcal/mol) for ethane formation from the hypothetical Au(III) complex [Au(CH_3_)_2_(PMe_2_Ar^Xyl2^)][NTf_2_] (Figure S15). However, [Au(CH_3_)_2_(PMe_2_Ar^Xyl2^)][NTf_2_] would be formed alongside the digold(0) species [Au_2_(PMe_2_Ar^Xyl2^)_2_], with these species being 42.1 kcal/mol higher in energy than its precursors, rendering this pathway inaccessible under the reaction conditions (Figure S15). Similarly, CH_3_^+^ transfer^36^ from **4a** to **1a** presents a computed transition state at +47.0 kcal/mol (**TS1** in Figure S16). In addition, we examined reductive coupling from the hypothetical trinuclear species derived from the above CH_3_^+^ transfer; a transition state at +33.6 kcal/mol was found (**TS2** in Figure S16), further suggesting that this pathway is unaffordable.

Finally, we considered the possible involvement of gold carbene (AuCH_2_) and hydride (AuH) species,^60^ potentially formed by hydride abstraction from Au-methyl complexes. However, the free energy cost to access these high-energy intermediates is calculated to be at least +36.2 kcal/mol (Figures S17 and S18), incompatible with the determined activation parameters. To further rule out this mechanistic route, we carried out an additional experiment with isotopically labeled [Au(CD_3_)(PMe_2_Ar^Xyl2^)] (**1a-*****d***_**3**_; see the Supporting Information for details). Treating an equimolar mixture of **1a** and **1a-*****d***_**3**_ with 2 equiv of Au(PMe_2_Ar^Xyl2^)(NTf_2_) yielded an approximate statistic mixture of CH_3_CH_3_, CH_3_CD_3_, and CD_3_CD_3_ (Figure S12), without further observable H/D scrambling, thus excluding the involvement of gold methylidene species.

Having ruled out the most direct mechanisms involving **1a** and **4a**, we decided to interrogate the participation of compounds Au(PR_2_Ar′)(NTf_2_), especially in consideration of the experimental results indicating that such complexes are accessible under the reaction conditions (see above). These compounds serve as a source of electrophilic [Au(PR_2_Ar′)]^+^ fragments^61,62^ and, as such, might facilitate or drive phosphine dissociation from other Au complexes. Potential phosphine dissociation is implicated from straightforward ligand exchange experiments (see Figure S13), and since it was proposed as the rate-limiting step in Kochi’s earlier system,^31,32^ it is conceivable that it could also play a role for C–C coupling from compounds **4**. To examine this, we monitored ethane evolution from **4a** in the presence of 3 equiv of Au(PMe_2_Ar^Xyl2^)(NTf_2_), though this excess of gold-triflimide did not have notable effects on the rate of ethane formation. This was, however, not surprising in line with our computational results, where the larger barrier originates after binding of [Au(PMe_2_Ar^Xyl2^)]^+^ to **4a**. Nonetheless, even if Au(PMe_2_Ar^Xyl2^)(NTf_2_) is required to facilitate phosphine dissociation, its presence may also affect the observed rate of ethane evolution in an opposite manner by reducing the concentration of Au(CH_3_)(PMe_2_Ar^Xyl2^) (**1a**) in solution, the latter species also being required for C–C coupling. This is because [Au_2_(μ-CH_3_)(PMe_2_Ar^Xyl2^)_2_][NTf_2_] (**4a**) is in dynamic equilibrium in solution with **1a** and Au(PMe_2_Ar^Xyl2^)(NTf_2_), as discussed above. To circumvent the influence of added Au(PMe_2_Ar^Xyl2^)(NTf_2_) on that equilibrium, we investigated the effect of adding 5 equiv of BPh_3_ as an alternative and less disruptive Lewis acid that could facilitate phosphine dissociation. While ethane evolution proceeded at a rate (*t*_1/2_ = 340 s) comparable to that of pure **4a** (*t*_1/2_ = 260 s), we did observe a distinctive change in the kinetic profile. More precisely, this experiment revealed a first-order kinetic dependence on **4a** (Figure S7), in contrast to the second-order profile observed when the consumption of the latter was monitored in pure form.

**Figure 9:**
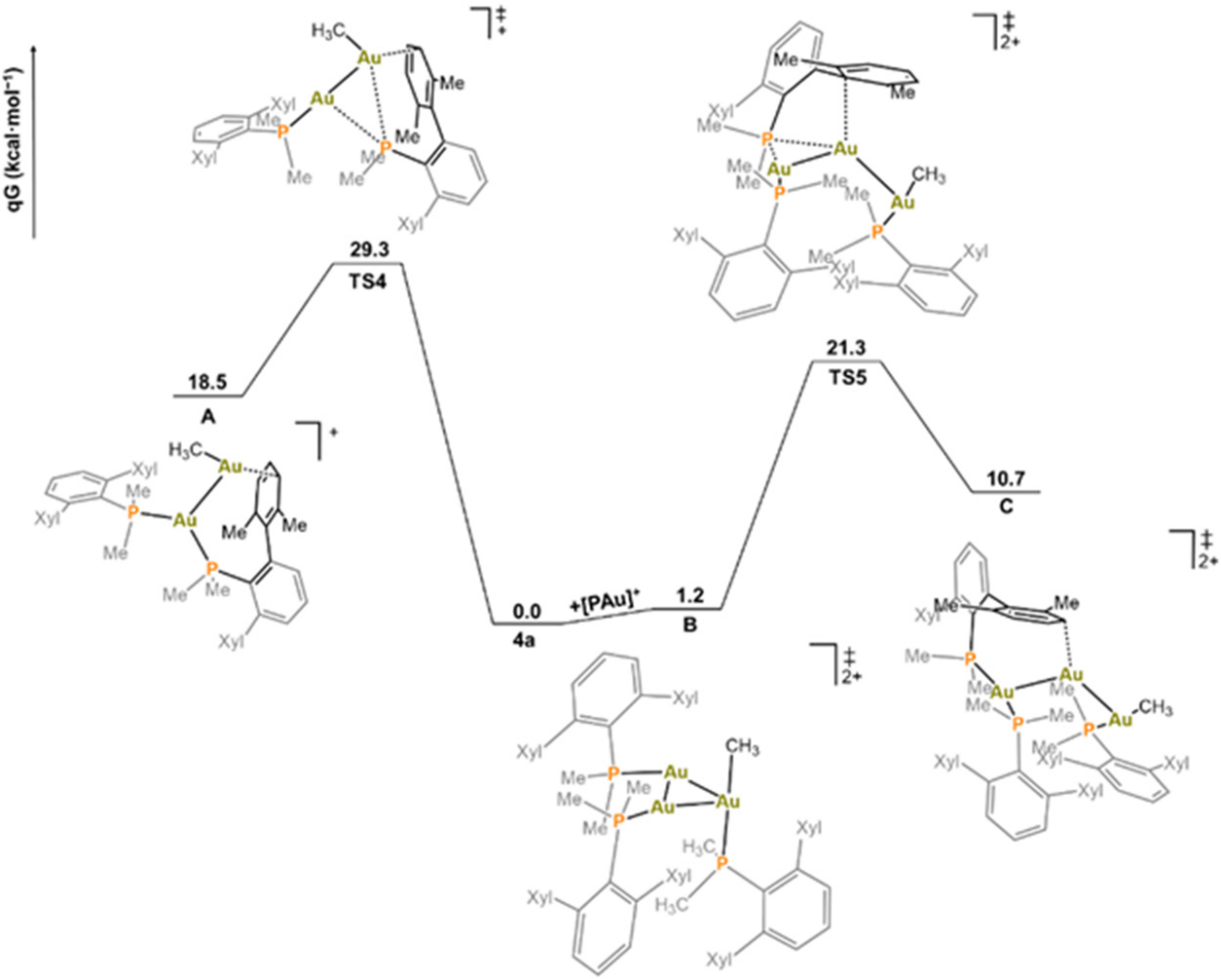
Free energy profile for [Au(PMe_2_Ar^Xyl2^)]^+^-promoted phosphine migration and formation of masked “AuMe” from Au(CH_3_)(PMe_2_Ar^Xyl2^) (**1a**, left) or [Au_2_(μ-CH_3_)(PMe_2_Ar^Xyl2^)_2_]^+^ (**4a**, right) complexes; calculated at the ωB97X-D/6-31G(d,p) level.

Next, we directed our efforts to examining, by computational means, the role of Au(PMe_2_Ar^Xyl2^)(NTf_2_) on the pathways and energetics for the formation of ethane (Figure 9). Since we attribute a Lewis acidic role to this fragment, as supported by our experiments with BPh_3_, we first studied BH_3_ as a simplified Lewis acid. Thus, we examined the reaction between BH_3_ and complex Au(CH_3_)(PMe_2_Ar^Xyl2^) (**1a**). The formation of a Au–BH_3_ adduct is slightly exergonic (Δ*G* = −0.9 kcal/mol), from which the transition state for the formation of a P–B bond (**TS3**) lies at +16.2 kcal/mol above the independently computed **1a** and BH_3_, giving the product at −7.4 kcal/mol (Figure S19). Encouraged by this result, we studied the analogous process with cation [Au(PMe_2_Ar^Xyl2^)]^+^ instead of BH_3_ as the Lewis acid.^63^ A transition state for that process (**TS4**) was found at +29.3 kcal/mol, leading to the formation of a species of formula [(PMe_2_Ar^Xyl2^)_2_AuAu(CH_3_)]^+^, **A** in Figure 9, that lies at +18.5 kcal/mol and represents a form of masked “AuMe” stabilized by aurophilic and metal–arene interactions with the [Au(PMe_2_Ar^Xyl2^)_2_]^+^ fragment. Nonetheless, the large barrier renders this process inaccessible from **4a**, in agreement with the experimentally determined second-order dependence on its concentration.

To account for the second-order dependence on **4a**, we considered its initial dissociation into **1a** and Au(PMe_2_Ar^Xyl2^)(NTf_2_), the latter providing 1 equiv of cation [Au(PMe_2_Ar^Xyl2^)]^+^ amenable to bind a second molecule of **4a**. The resulting trigonal dicationic adduct [Au_3_(μ-CH_3_)(PMe_2_Ar^Xyl2^)_3_]^2+^ (**B**) plus **1a** are only 1.2 kcal/mol above two molecules of **4a** (Figure 9). From trimetallic adduct **B**, the transition state for the formal transfer of a phosphine ligand between gold atoms was found at +21.3 kcal/mol (**TS5**), close enough to the experimentally determined value for the overall process of ethane evolution. This transition state gives trinuclear species **C** at +10.7 kcal/mol, from which dissociation of **5a** is thermodynamically accessible (Δ*G* = +2.5 kcal/mol). This would render the bimetallic intermediate [Au_2_(CH_3_)(PMe_2_Ar^Xyl2^)]^+^ (Figure S20), which is reminiscent of the proposed highly reactive “AuMe” fragment proposed by Kochi.^31,32^ From such a reactive fragment, masked as [Au_2_(CH_3_)(PMe_2_Ar^Xyl2^)]^+^, it is expected that the approach of **1a** would result in ethane elimination and formation of colloidal gold, not necessarily in that order.

**Scheme 8:**
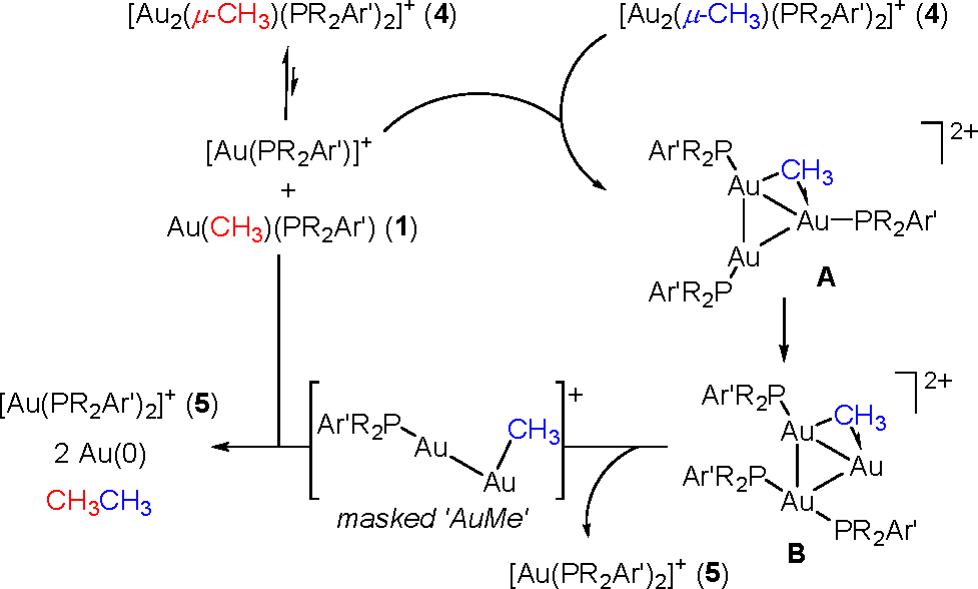
Proposed Mechanism for the Reductive Coupling of Ethane from gem-Digold Compounds **4**

Our combined experimental/computational approach led us to propose the mechanistic picture for C–C coupling at gem-digold compounds **4** depicted in Scheme 8. Compounds **4** readily dissociate in solution to form **1** and Au(PR_2_Ar′)(NTf_2_), the latter functioning as a Lewis acid to favor phosphine migration from a second molecule of **4** by forming a trimetallic intermediate of type **B**.^64^ Following the release of diphosphine compounds **5**, the resulting masked “AuMe” fragment reacts with **1a** to liberate ethane with concomitant formation of elemental Au, eventually leading to the formation of Au nanoparticles. In this picture, phosphine migration from **4a** constitutes the rate-limiting step of the overall process, in analogy to the previously proposed mechanism for reductive coupling from Au(CH_3_)(PPh_3_).^31,32^ In contrast, the remarkable acceleration observed for C–C coupling in compounds **4** compared to **1** seems to be the result of stabilization of key intermediates by the presence of aurophilic interactions combined with the Lewis acidic character of [Au(PR_2_Ar′)]^+^ that enables phosphine migration, thus representing an example of rate acceleration by polymetallic entities compared to monometallic counterparts.^65−67^

## Conclusions

Au-mediated C–C coupling processes have rapidly emerged as versatile and powerful strategies for organic synthesis. Despite numerous reports on the synthetic applicability of gold catalysts, mechanistic understanding has evolved at a slower pace. Previous studies have placed the Au(I)/Au(III) redox couple at the heart of all these transformations, while mechanistic investigations on C–C coupling processes without the apparent advent of Au(III) species is lacking. Herein, we have demonstrated that gem-digold methyl complexes [Au_2_(μ-CH_3_)(PR_2_Ar′)_2_][NTf_2_] (**4**) promote the homocoupling of the bridging methyl fragments to produce ethane at a remarkably higher rate than from its parent neutral species Au(CH_3_)(PR_2_Ar′) (**1**). We have also demonstrated that this approach permits the heterocoupling of the bridging methyl group with ethyl and phenyl fragments. The stability of compounds **4** toward reductive homocoupling is highly dependent on the steric bulk of the phosphine ligand. Whereas the system based on PPh_3_ readily liberates ethane at −40 °C, those bearing terphenyl phosphines (PMe_2_Ar′) exhibit considerably enhanced stability, which is further increased by the use of the more hindered XPhos and ^*t*^BuXPhos, the latter being unable to mediate C–C coupling even at 90 °C. Our kinetic studies revealed second-order dependence on gem-digold methyl complexes **4** during ethane evolution, whereas a distinctive change toward a first-order dependence on the latter was ascertained in the presence of excess BPh_3_ as an external Lewis acid. On the basis of our experimental studies combined with DFT computational methods we have proposed a mechanism that involves rapid dissociation of a molecule of [Au_2_(μ-CH_3_)(PMe_2_Ar′)_2_][NTf_2_] (**4**) toward Au(PMe_2_Ar′)(NTf_2_) and Au(CH_3_)(PMe_2_Ar′) (**1**). While Au(PMe_2_Ar′)(NTf_2_) mediates phosphine migration from a second molecule of **4** via a trimetallic intermediate, compound **1** is proposed to react with the resulting highly reactive and masked “AuMe” fragment to effect the C–C coupling event, most likely by a multinuclear gold species. These studies highlight the relevance of multimetallic mechanisms in mediating uncommon transformations, herein also boosting the rate at which the C–C coupling transformation occurs.

## Experimental Section

### General Methods

**Figure 10:**
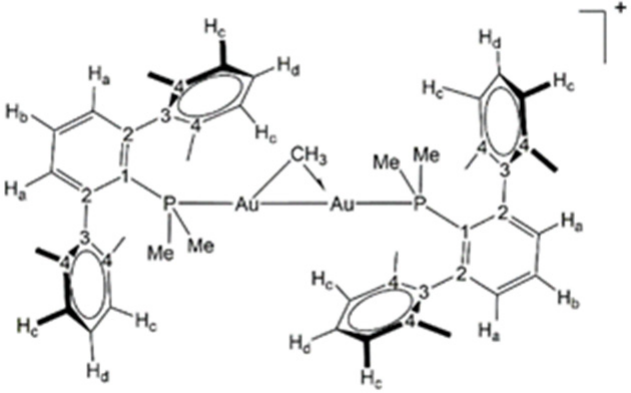
Labeling scheme used for ^1^H and ^13^C{^1^H} NMR assignments.

Unless otherwise noted, all reactions and manipulations were performed under a nitrogen atmosphere in a glovebox or using standard Schlenk techniques with dried and degassed solvents. All solvents were purified via a solvent purification system or by common distillation techniques: Dichloromethane (CH_2_Cl_2_) was distilled under nitrogen over CaH_2_. Toluene (C_7_H_8_), benzene (C_6_H_6_), *n*-hexane (C_6_H_14_), and *n*-pentane (C_5_H_12_) were distilled under nitrogen over sodium. Tetrahydrofuran (THF) and diethyl ether were distilled under nitrogen over sodium/benzophenone. Benzene (C_6_D_6_) was dried over sodium, while CDCl_3_ and CD_2_Cl_2_ were dried over molecular sieves (4 Å) and distilled under nitrogen. Compounds PMe_2_Ar′,^68^ AuCl(THT) (THT = tetrahydrothiophene),^69^ Au(PPh_3_)(NTf_2_),^70,71^ Au(PPh_3_)(NO_3_),^72,73^ AuCl(XPhos),^74^ AuCl(^*t*^BuXPhos),^75^ Au(XPhos)(NTf_2_),^76−78^ Au(^*t*^BuXPhos)(NTf_2_),^78^ AuCl(PMe_2_Ar^Xyl2^),^62^ AuCl(PMe_2_Ar^Dipp2^),^46^ AuCl(PMe_2_Ar^Tripp2^),^79^ Au(PMe_2_Ar^Xyl2^)(NTf_2_),^62^ Au(PMe_2_Ar^Dipp2^)(NTf_2_),^46^ Au(PMe_2_Ar^Tripp2^)(NTf_2_),^79^ Au(CH_3_)(PMe_2_Ar^Dipp2^)^46^ (**1c**), and [Au_2_(μ-CH_3_)(PMe_2_Ar^Dipp2^)_2_][NTf_2_]^46^ (**4c**) were prepared according to previously reported procedures. Compounds **1e** and **1f** were prepared according to the general method described below in yields of around 75%, exhibiting identical spectroscopic data to those previously reported. Au(CH_3_)(XPhos)^78^ and Au(CH_3_)(^*t*^BuXPhos)^80^ were prepared by an alternative method of the published procedures and fully characterized. Methyl(triphenylphosphine)gold(I), chloro(dimethylsulfide)gold(I), silver bis(trifluoromethanesulfonyl)imide acetonitrile adduct, chlorotriphenylphosphinegold(I), 2-dicyclohexylphosphino-2′,4′,6′-triisopropylbiphenyl (XPhos), and 2-di-*tert*-butylphosphino-2′,4′,6′-triisopropylbiphenyl (*^t^*BuXPhos) were purchased from STREM Chemicals and were used as received. Other chemicals were purchased from Sigma-Aldrich and used as received. All new compounds have been characterized by ^1^H NMR spectroscopy, ^31^P NMR spectroscopy, ^13^C NMR spectroscopy, and elemental analysis (see Figure 10). Solution NMR spectra were recorded on Varian Inova 600 or 500 MHz or on Bruker AMX-300, DRX-400, DRX-500, and Avance III 800 MHz spectrometers. Spectra were referenced to external SiMe_4_ or using the residual proton solvent peaks as internal standards (^1^H NMR experiments), or the characteristic resonances of the solvent nuclei (^13^C NMR experiments), while ^31^P was referenced to H_3_PO_4_. Spectral assignments were made by routine one- and two-dimensional NMR experiments where appropriate. For elemental analyses, the LECO TruSpec CHN elementary analyzer and PerkinElmer 2400 Series II analyzer were utilized. GC analysis was performed using a Shimadzu GCMSQP2010-Plus instrument equipped with a PoraBOND-Q capillary column (25 m, 0.25 mm i.d., 3.0 μm film thickness, Agilent Technologies). Helium carrier gas was supplied at a head pressure of 10 psi to provide an initial flow rate of 1.4 mL/min. A 1 mL injection with a split ratio of 1:10 was employed. GC temperature was initially held at 40 °C for 1 min and gradually increased to 120 °C at 5 °C/min. Full-scan mass spectra were collected from 5 to 70 *m*/*z* at a data acquisition rate of 3.5 spectra/s. The MS transfer line was held at 250 °C, and the ion source temperature was 200 °C. Samples analyzed by transmission electron microscopy (TEM) were prepared by dispersing the powders in cyclohexane or hexanes (99.5%, anhydrous, Sigma-Aldrich) and sonicating for 1 min before mounting on Cu-supported holey carbon grids. The Au samples were imaged using an FEI Titan 80-300 operating at 300 kV. The structures of compounds **1a**, **1e**, **1f**, **2a**, **3a**, **4e**, **4f**, and Au(^*t*^BuPhos)(NTf_2_) have been authenticated by X-ray diffraction studies and their corresponding CIF files deposited in the Cambridge Crystallographic Data Centre with nos. 2024182–2024189.

### General Synthesis of Compounds **1**

A suspension of the corresponding gold chloride precursor AuCl(PR_2_Ar′) (0.20 mmol) in toluene (10 mL) was cooled to −78 °C, and a solution of MeMgX (X = Cl or Br; 2.5 equiv) in toluene was added dropwise. The mixture was allowed to warm up slowly for 16 h. The volatiles were removed in vacuo, and the residue was extracted with benzene for **1a**–**1d** or pentane for **1e**–**1f**. Evaporation of the organic solvent led to compounds **1a**–**1f** as white powders in around 60–80% yields. Isotopologue **1a-*****d***_**3**_ was synthesized by the same procedure using freshly prepared CD_3_MgI.^81^ Suitable crystals of compounds **1** can be obtained by slow solvent evaporation from pentane/Et_2_O or pentane/dichloromethane solutions. Spectroscopic and analytical data for selected compounds (others can be found in the SI). Compound **1a**. Yield: 84 mg, 75%. Anal. Calcd for C_25_H_30_AuP: C, 53.8; H, 5.4. Found: C, 53.4; H, 5.5. ^1^H NMR (400 MHz, CD_2_Cl_2_, 25 °C) δ: 7.53 (td, 1 H, ^5^*J*_HP_ = 1.7 Hz, H_b_), 7.23 (t, 2 H, H_d_), 7.14 (d, 4 H, H_c_), 7.07 (dd, 2 H, ^4^*J*_HP_ = 2.9 Hz, H_a_), 2.13 (s, 12 H, *C*H_3_(Xyl)), 1.02 (d, 6 H, ^2^*J*_HP_ = 7.7 Hz, PMe_2_), −0.08 (d, 3 H, ^3^*J*_HP_ = 8.2 Hz, AuCH_3_). All aromatic couplings are of ca. 7.5 Hz. ^13^C{^1^H} NMR (100 MHz, CD_2_Cl_2_, 25 °C) δ: 147.0 (d, ^2^*J*_CP_ = 10 Hz, C_2_), 142.3 (d, ^4^*J*_CP_ = 4 Hz, C_3_), 137.2 (C_4_), 131.8 (d, ^1^*J*_CP_ = 35 Hz, C_1_), 131.6 (d, ^4^*J*_CP_ = 3 Hz, CH_b_), 131.5 (d, ^3^*J*_CP_ = 7 Hz, CH_a_), 128.6 (CH_d_), 128.4 (CH_c_), 22.4 (*C*H_3_(Xyl)), 16.8 (d, ^1^*J*_CP_ = 30 Hz, PMe_2_), 4.7 (d, ^2^*J*_CP_ = 100 Hz, AuCH_3_). ^31^P{^1^H} NMR (162 MHz, CD_2_Cl_2_, 25 °C) δ: 22.1. MS (ESI) m/*z*: calcd for M(Na)^+^, 581.2; expt., 581.4. Compound **1d**. Yield: 106 mg, 70%. Anal. Calcd for C_39_H_58_AuP: C, 62.1; H, 7.7. Found: C, 62.0; H, 7.5. ^1^H NMR (300 MHz, CD_2_Cl_2_, 25 °C) δ: 7.42 (td, 1 H, ^5^*J*_HP_ = 1.8 Hz, H_b_), 7.15 (dd, 2 H, ^4^*J*_HP_ = 3.0 Hz, H_a_), 7.08 (s, 4 H, H_c_), 2.94 (hept, 2 H, ^3^*J*_HH_ = 6.9 Hz, *p*-^*i*^Pr(CH)), 2.58 (hept, 4 H, ^3^*J*_HH_ = 6.9 Hz, *o*-^*i*^Pr(CH)), 1.31 (d, 12 H, ^3^*J*_HH_ = 6.9 Hz; d, 12 H, ^3^*J*_HH_ = 6.9 Hz, *o-*^*i*^Pr(CH_3_), *p-*^*i*^Pr(CH_3_)), 1.07 (d, 6 H, ^2^*J*_HP_ = 7.4 Hz, PMe_2_), 1.02 (d, 12 H, ^3^*J*_HH_ = 6.9 Hz, *o*-^*i*^Pr(CH_3_)), −0.36 (d, 3 H, ^3^*J*_HP_ = 8.2 Hz, AuCH_3_). All aromatic couplings are of ca. 7.5 Hz. ^13^C{^1^H} NMR (125 MHz, CD_2_Cl_2_, 25 °C) δ: 149.9 (C_5_), 146.9 (C_4_), 146.6 (d, ^2^*J*_CP_ = 11 Hz, C_2_), 137.9 (d, ^4^*J*_CP_ = 5 Hz, C_3_), 133.7 (C_1_), 133.4 (d, ^3^*J*_CP_ = 7 Hz, CH_a_), 129.3 (CH_b_), 121.8 (CH_c_), 35.1 (*p*-^*i*^Pr(CH)), 31.9 (*o*-^*i*^Pr(CH)), 26.1 (*o*-^*i*^Pr(CH_3_)), 24.9 (*p*-^*i*^Pr(CH_3_)), 23.4 (*o*-^*i*^Pr(CH_3_)), 17.3 (d, ^1^*J*_CP_ = 30 Hz, PMe_2_), 5.7 (d, ^2^*J*_CP_ = 102 Hz, AuCH_3_). ^31^P{^1^H} NMR (121 MHz, CD_2_Cl_2_, 25 °C) δ: 19.8. MS (ESI) m/*z*: calcd for M(Na)^+^, 777.4; expt., 777.5.

### General Synthesis of Compounds **4**

A solid mixture of the corresponding methyl gold precursor **1a**–**1f** (0.0175 mmol) with 1 equiv of its parent compound [Au(PR_2_Ar′)][NTf_2_](0.0175 mmol) was dissolved in CD_2_Cl_2_ (0.6 mL) under nitrogen at −50 °C to rapidly yield the desired methyl-bridged complex **4a**–**4f** in a quantitative NMR spectroscopic yield. Characterization of the less stable compounds **4a** and **4b** was carried out by multinuclear NMR spectroscopy at low temperature without further purification. Compounds **4c**–**4f** were obtained as colorless microcrystalline substances by precipitation with pentane at −20 °C (**4c**, **4d**) or 25 °C (**4e**, **4f**) in around 90% yields. Alternatively **4a**–**4f** can be prepared in comparable by treating compounds **1a**–**1f** with 1/2 equiv of [Ph_3_C][B(C_6_F_5_)_4_] in dichloromethane by an otherwise identical procedure. Spectroscopic and analytical data for selected compounds (others can be found in the SI). Compound **4a**. ^1^H NMR (400 MHz, CD_2_Cl_2_, −30 °C) δ: 7.61 (t, 2 H, H_b_), 7.25 (t, 4 H, H_d_), 7.08 (m, 12 H, H_a_, H_c_), 1.98 (s, 24 H, *C*H_3_(Xyl)), 1.16 (d, 12 H, ^2^*J*_HP_ = 7.7 Hz, PMe_2_), 0.45 (br. s, 3 H, AuCH_3_···Au). All aromatic couplings are of ca. 7.5 Hz. ^13^C{^1^H} NMR (100 MHz, CD_2_Cl_2_, −30 °C) δ: 147.1 (d, ^2^*J*_CP_ = 11 Hz, C_2_), 141.1 (d, ^4^*J*_CP_ = 5 Hz, C_3_), 136.7 (C_4_), 133.3 (CH_b_), 131.8 (d, ^3^*J*_CP_ = 8 Hz, CH_a_), 129.0 (CH_d_), 128.2 (CH_c_), 127.8 (d, ^1^*J*_CP_ = 38 Hz, C_1_), 21.9 (*C*H_3_(Xyl)), 16.9 (d, ^1^*J*_CP_ = 37 Hz, PMe_2_), 0.6 (AuCH_3_···Au). ^31^P{^1^H} NMR (162 MHz, CD_2_Cl_2_, −20 °C) δ: 1.1. Compound **4d**. Anal. Calcd for C_101_H_113_Au_2_BF_20_P_2_: C, 55.8; H, 5.2. Found: C, 56.1; H, 4.9. ^1^H NMR (400 MHz, CD_2_Cl_2_, 25 °C) δ: 7.51 (t, 2 H, H_b_), 7.17 (dd, 4 H, ^4^*J*_HP_ = 3.3 Hz, H_a_), 7.05 (s, 8 H, H_c_), 2.94 (hept, 4 H, ^3^*J*_HH_ = 7.0 Hz, *p*-^*i*^Pr(CH)), 2.41 (hept, 8 H, ^3^*J*_HH_ = 6.7 Hz, *o*-^*i*^Pr(CH)), 1.31 (d, 24 H, ^3^*J*_HH_ = 7.0 Hz, *p-*^*i*^Pr(CH_3_)), 1.22 (m, 36 H,*o-*^*i*^Pr(CH_3_), PMe_2_), 1.00 (d, 24 H, ^3^*J*_HH_ = 6.6 Hz, *o*-^*i*^Pr(CH_3_)), 0.25 (s, 3 H, AuCH_3_···Au). All aromatic couplings are of ca. 7.5 Hz. ^13^C{^1^H} NMR (100 MHz, CD_2_Cl_2_, 25 °C) δ: 151.1 (C_5_), 147.3 (C_4_), 146.8 (d, ^2^*J*_CP_ = 12 Hz, C_2_), 137.0 (d, ^4^*J*_CP_ = 6 Hz, C_3_), 134.0 (d, ^3^*J*_CP_ = 8 Hz, CH_a_), 131.2 (CH_b_), 129.4 (d, ^1^*J*_CP_ = 56 Hz, C_1_), 122.2 (CH_c_), 35.0 (*p*-^*i*^Pr(CH)), 32.0 (*o*-^*i*^Pr(CH)), 25.8 (*o*-^*i*^Pr(CH_3_)), 24.8 (*p*-^*i*^Pr(CH_3_)), 23.5 (*o*-^*i*^Pr(CH_3_)), 17.7 (d, ^1^*J*_CP_ = 37 Hz, PMe_2_), 0.5 (t, ^2^*J*_CP_ = 53 Hz, ^1^*J*_CH_ = 130 Hz, AuCH_3_···Au). ^31^P{^1^H} NMR (162 MHz, CD_2_Cl_2_, 25 °C) δ: 0.5. MS (ESI) m/*z*: calcd for M^+^, 1493.8; expt., 1493.8. Compound **4e**. Anal. Calcd for C_69_H_101_Au_2_F_6_NO_4_P_2_S_2_: C, 50.5; H, 6.2; N, 0.9. Found: C, 50.3; H, 6.2; N, 0.9. ^1^H NMR (500 MHz, CD_2_Cl_2_, 25 °C) δ: 7.76 (m, 2 H, H_a_), 7.64 (m, 4 H, H_b_), 7.22 (m, 2 H, H_c_), 7.09 (s, 4 H, H_d_), 3.06 (hept, 2 H, ^3^*J*_HH_ = 7.1 Hz, *o*-^*i*^Pr(CH), 2.37 (hept, 4 H, ^3^*J*_HH_ = 7.0 Hz, *p*-^*i*^Pr(CH)), 2.15 (m, 2 H, Cy(CH_2_)), 1.92 (m, 8 H, Cy(CH_2_)), 1.85 (m, 2 H, Cy(CH)), 1.46 (m, 8 H, Cy(CH)), 1.44 (d, 12 H, ^3^*J*_HH_ = 6.0 Hz, *p*-^*i*^Pr(CH_3_)), 1.37 (m, 8 H, Cy(CH)), 1.24 (m, 8 H, Cy(CH)), 1.26 (d, 12 H, ^3^*J*_HH_ = 6.0 Hz, *o*-^*i*^Pr(CH_3_)), 1.03 (d, 6 H, ^3^*J*_HH_ = 6.0 Hz, *o*-^*i*^Pr(CH_3_), c), 0.67 (t, 3 H, ^3^*J*_HP_ = 2.2 Hz, AuCH_3_···Au). ^13^C{^1^H} NMR (201 MHz, CD_2_Cl_2_, 25 °C) δ: 150.3, 147.1, 146.7 (d, *J* = 14 Hz), 137.2 (d, *J* = 6 Hz), 134.2 (d, *J* = 10 Hz), 133.2, 131.1, 127.8 (d, *J* = 6 Hz), 127.5 (d, ^2^*J*_C–P_ = 48 Hz), 121.3, 37.5 (d, *J* = 32 Hz), 34.2, 30.8 (d, *J* = 4 Hz), 30.8, 30.0 (d, *J* = 4 Hz), 26.8 (d, *J* = 12 Hz), 26.7 (d, *J* = 14 Hz), 25.7, 24.9, 24.2, 23.0, 3.1 (t, ^2^*J*_CP_ = 48 Hz). ^31^P{^1^H} NMR (243 MHz, CD_2_Cl_2_, 25 °C) δ: 39.5.

### General Procedure to Measure Kinetic Constants

Kinetic studies were carried using an identical procedure to that described for the general synthesis of compounds **4**, in J-Young NMR tubes under nitrogen atmosphere, and monitoring the disappearance of the *in situ* formed gem-digold methyl compounds **4** by ^1^H and ^31^P{^1^H} NMR. Each kinetic experiment was run in triplicates, and average data are given.

## Supporting Information Available

Synthesis and characterization of new compounds, X-ray diffraction data, information on kinetic studies, variable temperature analysis and exchange experiments, DFT calculations, and MS and NMR spectra (PDF)
XYZ coordinates (XYZ)

## Supplementary Material

ja0c11296_si_001.pdf

ja0c11296_si_003.xyz

## Accession Codes

CCDC 2024182–2024189 contain the supplementary crystallographic data for this paper. These data can be obtained free of charge via www.ccdc.cam.ac.uk/data_request/cif, or by emailing data_request@ccdc.cam.ac.uk, or by contacting The Cambridge Crystallographic Data Centre, 12 Union Road, Cambridge CB2 1EZ, UK; fax: +44 1223 336033.
