# Supplementary material for: Reductive C–C Coupling from Molecular Au(I) Hydrocarbyl Complexes: A Mechanistic Study
Source: J Am Chem Soc. 2021 Feb 5;143(6):2509–22. doi: 10.1021/jacs.0c11296 (PMC8479859; doi:10.1021/jacs.0c11296)
Supplement: Supplementary file 1 — ja0c11296_si_001.pdf [file ja0c11296_si_001.pdf]

Supporting Information for

Reductive C–C coupling from Molecular Au(I) Hydrocarbyl Complexes: A Mechanistic Study

[1]Juan Miranda-Pizarro[ᵀ], [2]Zhongwen Luo[ᵀ], [1]Juan J. Moreno, [2]Diane A. Dickie, [1]Jesús Campos* and [2]T. Brent Gunnoe*

[1] Instituto de Investigaciones Químicas (IIQ), Departamento de Química Inorgánica and Centro de Innovación en Química Avanzada (ORFEO-CINQA). Universidad de Sevilla and Consejo Superior de Investigaciones Científicas (CSIC), Avenida Américo Vespucio 49, 41092 Sevilla, Spain

[2] Department of Chemistry, University of Virginia, Charlottesville, VA 22904, USA

Table of Contents

I. Synthesis and characterization of new gold (I) complexes ……………………….……….S2

II. X-Ray Structural Characterization of new compounds……………………..………S17

III. Kinetic studies.…………………………………………………………….……………..…...S21

IV. Variable temperature experiments and exchange experiments…………..……….S24

V. DFT calculations…………………………………………………………………...……S29

VI. Mass spectroscopy and ^{1}H NMR confirmation of gas sample ethane……………..………S33

VII. NMR spectra of gold (I) compounds……………………………………………..………S35

VIII. References……………………………………………..……...………………...S65

I. Synthesis and characterization of new gold (I) complexes

Compounds $AuCl(PMe_2Ar')$

General synthesis of compounds $AuCl(PMe_2Ar')$. The corresponding terphenyl phosphine (PMe_2Ar^{Xyl2}, PMe_2Ar^{Mes2}, PMe_2Ar^{Dipp2}, PMe_2Ar^{Trip2}: 0.94 mmol) and [Au(tht)Cl] (tht = tetrahydrotiophene) (300 mg, 0.94 mmol) were dissolved in toluene (10 mL) in a Schlenk flask under nitrogen. The initial white suspension became a solution after several hours and was stirred for an overall period of 12 hours. The solvent was removed under vacuum and the resulting white solid washed with pentane and dried to give the corresponding terphenyl phosphine gold chloride complexes as fine white powders in around 90% yields. These complexes can be recrystallized from a 1:3 mixture of CH_2Cl_2/pentane.

$AuCl(PMe_2Ar^{Mes2})$, Ar^{Mes2} =C_6H_3-2,6-(C_6H_2-2,4,6-Me_3)$_2$

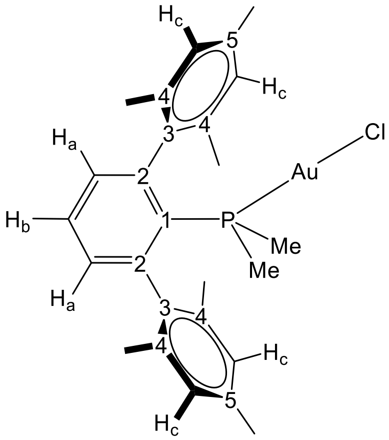

Anal. Calcd. for $C_{26}H_{31}AuClP$: C, 51.5; H, 5.2. **Found:** C, 51.0; H, 5.3.

^{1}H NMR (400 MHz, CD_2Cl_2, 25 ºC) δ: 7.58 (td, 1 H, $^5J_{HP}$ = 1.9 Hz, H_b), 7.08 (dd, 2 H, $^4J_{HP}$ = 3.5 Hz, H_a), 6.98 (s, 4 H, H_c), 2.35 (s, 6 H, *p*-CH_3(Mes)), 2.08 (s, 12 H, *o*-CH_3(Mes)), 1.20 (d, 6 H, $^2J_{HP}$ = 10.5 Hz, PMe_2). All aromatic couplings are of *ca*. 7.5 Hz.

^{13}C{^{1}H} NMR (100 MHz, CD_2Cl_2, 25 ºC) δ: 147.1 (d, $^2J_{CP}$ = 10 Hz, C_2), 139.1 (C_5), 138.5 (d, $^4J_{CP}$ = 5 Hz, C_3), 136.8 (C_4), 132.5 (d, $^4J_{CP}$ = 3 Hz, CH_b), 132.2 (d, $^3J_{CP}$ = 8 Hz, CH_a), 129.5 (CH_c), 127.9 (d, $^1J_{CP}$ = 58 Hz, C_1), 22.2 (*p*-CH_3(Mes)), 21.7 (*o*-CH_3(Mes)), 18.0 (d, $^1J_{CP}$ = 40 Hz, PMe_2).

^{31}P{^{1}H} NMR (162 MHz, CD_2Cl_2, 25 ºC) δ: -3.5.

MS (ESI) m/*z* Calcd. for $M(Na)^+$: 629.1. **Expt.:** 629.3.

Compounds $Au(CH_3)(PR_2Ar')$ (1a-f)

$Au(CH_3)(PMe_2Ar^{Mes2})$, Ar^{Mes2} =C_6H_3-2,6-(C_6H_2-2,4,6-Me_3)$_2$, (1b)

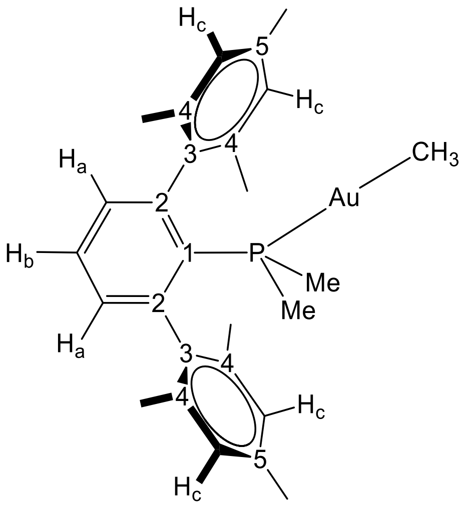

Yield: 70 mg, 60 %

Anal. Calcd. for $C_{27}H_{34}AuP$: C, 55.3; H, 5.8. **Found**: C, 54.9; H, 5.5.

^{1}H NMR (300 MHz, CD_2Cl_2, 25 ºC) δ: 7.49 (td, 1 H, $^5J_{HP}$ = 1.7 Hz, H_b), 7.03 (dd, 2 H, $^4J_{HP}$ = 2.9 Hz, H_a), 6.95 (s, 4 H, H_c), 2.34 (s, 6 H, *p*-CH_3(Mes)), 2.08 (s, 12 H, *o*-CH_3(Mes)), 1.03 (d, 6 H, $^2J_{HP}$ = 7.7 Hz, PMe_2), -0.08 (d, 3 H, $^3J_{HP}$ = 8.2 Hz, $AuCH_3$). All aromatic couplings are of *ca*. 7.5 Hz.

^{13}C{^{1}H} NMR (100 MHz, CD_2Cl_2, 25 ºC) δ: 147.1 (d, $^2J_{CP}$ = 10 Hz, C_2), 139.5 (d, $^4J_{CP}$ = 4 Hz, C_3), 138.3 (C_5), 136.1 (C_4), 132.4 (d, $^1J_{CP}$ = 36 Hz, C_1), 131.7 (d, $^4J_{CP}$ = 7 Hz, CH_a), 131.4 (d, $^3J_{CP}$ = 2 Hz, CH_b), 128.2 (CH_c), 22.3 (*o*-CH_3(Mes)), 21.7 (*p*-CH_3(Mes)), 17.0 (d, $^1J_{CP}$ = 30 Hz, PMe_2), 3.4 (d, $^2J_{CP}$ = 100 Hz, $AuCH_3$).

^{31}P{^{1}H} NMR (160 MHz, CD_2Cl_2, 25 ºC) δ: 21.1.

MS (ESI) m/*z* Calcd. for $M(Na)^+$: 609.2. **Expt.**: 609.3.

Au(CH_3)(XPhos) (1e)

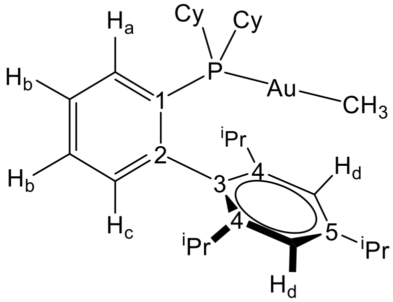

Yield: 103 mg, 75%.

Anal. Calcd. for $C_{34}H_{52}AuP$: C, 59.3; H, 7.6. **Found**: C, 59.1; H, 7.8.

^{1}H NMR (500 MHz, CD_2Cl_2, 25 ºC) δ: 7.63 (td, 1 H, $^3J_{HH}$ = 7.5 Hz, H_a), 7.46 (q, 2 H, $^3J_{HH}$ = 7.7 Hz, H_b), 7.18 (m, 1 H, H_c), 7.05 (s, 2 H, H_d), 2.95 (hept, 1 H, $^3J_{HH}$ = 6.9 Hz, *o*-^{i}Pr(CH)), 2.34 (hept, 2 H, $^3J_{HH}$ = 7.0, *p*-^{i}Pr(CH)), 2.18 (m, 2 H, Cy(CH)), 2.1 (m, 2 H, Cy(CH)), 1.84 (m, 4 H, Cy(CH)), 1.76 (m, 2 H, Cy(CH)), 1.69 (m, 2 H, Cy(CH)), 1.38 (m, 2 H, Cy(CH)), 1.24 (m, 6 H, Cy(CH)), 1.34 (d, 6 H, $^3J_{HH}$ = 6.9 Hz, *p*-^{i}Pr(CH_3)), 1.31 (d, 6 H, $^3J_{HH}$ = 7.0 Hz, *o*-^{i}Pr(CH_3)), 0.96 (d, 6 H, $^3J_{HH}$ = 6.8 Hz, *o*-^{i}Pr(CH_3), (c)), -0.41 (d, 3 H, $^3J_{HP}$ = 7.6 Hz, AuCH_3).

^{13}C{^{1}H} NMR (201 MHz CD_2Cl_2, 25 ºC)[1] δ: 148.6, 147.4 (d, *J* = 18.1 Hz), 145.9, 137.4 (d, *J* = 4.0 Hz), 133.4 (d, *J* = 8.0 Hz), 131.5 (d, *J* = 34.2 Hz), 129.5 (d, *J* = 2.0 Hz), 126.7 (d, $^2J_{CP}$ = 6.0 Hz), 121.0, 37.5 (d, *J* = 26.1 Hz), 34.2, 30.8, 30.5 (d, *J* = 4.0 Hz), 30.0 (d, *J* = 4.0 Hz), 27.2 (d, *J* = 12.1 Hz), 26.9 (d, *J* = 12.1 Hz), 26.0, 25.3, 24.2, 22.62, 8.3 (d, $^2J_{CP}$ = 94.5 Hz).

^{31}P{^{1}H} NMR (243 MHz, CD_2Cl_2, 25 ºC) δ: 47.5.

$Au(CH_3)(^tBuXPhos)$ (1f)

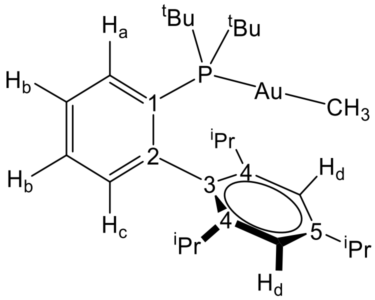

Yield: 93 mg, 73%.

Anal. Calcd. for $C_{30}H_{48}AuP$: C, 56.6; H, 7.6. **Found**: C, 56.5; H, 7.2.

^{1}H NMR (500 MHz, CD_2Cl_2, 25 ºC) δ: 7.92 (td, 1 H, $^3J_{HH}$ = 7.7 Hz, H_a), 7.46 (q, 2 H, $^3J_{HH}$ = 7.5 Hz, H_b), 7.22 (m, 1 H, H_c), 7.04 (s, 2 H, H_d), 2.95 (hept, 1 H, $^3J_{HH}$ = 6.7 Hz, *p*-^{i}Pr(CH)), 2.44 (hept, 2 H, $^3J_{HH}$ = 6.6 Hz, *o*-^{i}Pr(CH)), 1.42 (d, 18 H, $^3J_{HP}$ = 14.2 Hz, ^{t}Bu), 1.34 (d, 6 H, $^3J_{HH}$ = 6.9 Hz, *p*-^{i}Pr(CH_3)), 1.30 (d, 6 H, $^3J_{HH}$ = 7.0 Hz, *o*-^{i}Pr(CH_3)), 0.92 (d, 6 H, $^3J_{HH}$ = 6.9 Hz, *o*-^{i}Pr(CH_3)), -0.45 (d, 3 H, $^3J_{HP}$ = 7.4 Hz, $AuCH_3$).

^{13}C{^{1}H} NMR (201 MHz, CD_2Cl_2, 25 ºC)[1] δ: 148.6, 148.2, 146.1, 137.8, 136.4, 134.62, 132.19 (d, $^1J_{C\text{-}P}$ = 28.1 Hz), 129.36, 126.0, 121.1, 37.75 (d, $^1J_{C\text{-}P}$ = 18 Hz), 34.1, 31.15 (d, $^2J_{C\text{-}P}$ = 6.0 Hz), 30.71, 25.79, 24.13, 22.55, 6.2 (d, $^2J_{C\text{-}P}$ = 90.5 Hz).

^{31}P{^{1}H} NMR (243 MHz, CD_2Cl_2, 25 ºC) δ: 70.6.

Compound $Au(CH_2CH_3)(PMe_2Ar^{Xyl2})$ (2a)

A suspension of $AuCl(PMe_2Ar^{Xyl2})$ (116 mg, 0.20 mmol) in toluene (10 mL) was cooled to -78 ºC and a commercial solution of EtMgBr in Et_2O (3 M, 130 μL, 0.4 mmol) was added dropwise. The mixture was allowed to warm up slowly for 16 hours. The volatiles were removed in vacuum and the residue extracted with benzene. Evaporation of the organic solvent led to compound **2a** as an analytically pure white powder (69 mg, 60%). Suitable crystals of **2a** can be obtained by slow solvent evaporation from pentane/Et_2O solutions.

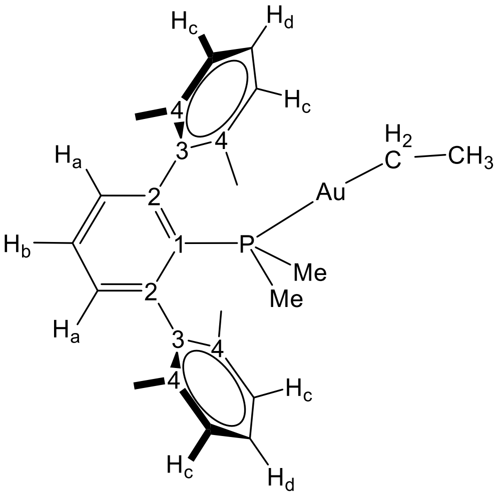

Anal. Calcd. for $C_{26}H_{32}AuP$: C, 54.6; H, 5.6. **Found**: C, 54.7; H, 5.2.

^{1}H NMR (300 MHz, CD_2Cl_2, 25 ºC) δ: 7.52 (td, 1 H, $^5J_{HP}$ = 1.6 Hz, H_b), 7.23 (m, 2 H, H_d), 7.13 (d, 4 H, H_c), 7.05 (dd, 2 H, $^4J_{HP}$ = 2.7 Hz, H_a), 2.15 (s, 12 H, CH_3(Xyl)), 1.16 (q, 3 H, $^3J_{HH}$ = 7.7 Hz, $AuCH_2CH_3$), 0.94 (d, 6 H, $^2J_{HP}$ = 7.4 Hz, PMe_2), 0.81 (quint, 2 H, $^3J_{HH}$ = 7.7 Hz, $AuCH_2CH_3$). All aromatic couplings are of *ca*. 7.5 Hz.

^{13}C{^{1}H} NMR (100 MHz, CD_2Cl_2, 25 ºC) δ: 146.7 (d, $^2J_{CP}$ = 9 Hz, C_2), 142.2 (d, $^3J_{CP}$ = 3 Hz, C_3), 137.2 (C_4), 131.4 (d, $^3J_{CP}$ = 7 Hz, CH_a), 131.4 (s, CH_b), 123.2 (d, $^1J_{CP}$ = 40 Hz, C_1), 128.7 (s, CH_d), 128.4 (CH_c), 22.4 (CH_3(Xyl)), 19.8 (d, $^2J_{CP}$ = 102 Hz, $AuCH_2CH_3$), 17.3 (d, $^3J_{CP}$ = 4 Hz, $AuCH_2CH_3$), 16.7 (d, $^1J_{CP}$ = 30 Hz, PMe_2).

^{31}P{^{1}H} NMR (160 MHz, C_6D_6, 25 ºC) δ: 23.1.

Compound Au(C_6H_5)(PMe_2Ar^{Xyl2}) (3a)

A suspension of AuCl(PMe_2Ar^{Xyl2}) (116 mg, 0.20 mmol) in toluene (10 mL) was cooled to -78 ºC and a commercial solution of PhLi in dibutyl ether (1.8 M, 220 μL, 0.4 mmol) was added dropwise. The mixture was allowed to warm up slowly for 16 hours. The volatiles were removed in vacuum and the residue extracted with benzene. Evaporation of the organic solvent led to compound **3a** as an analytically pure white powder (77 mg, 62%). Suitable crystals of **3a** can be obtained by slow solvent evaporation from pentane/Et_2O solutions.

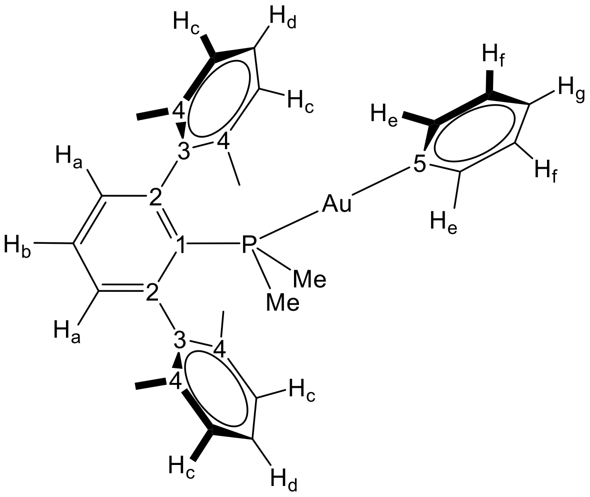

Anal. Calcd. for $C_{30}H_{32}AuP$: C, 58.1; H, 5.2. **Found**: C, 58.1; H, 5.4.

^{1}H NMR (400 MHz, CD_2Cl_2, 25 ºC) δ: 7.59 (td, 1 H, $^5J_{HP}$ = 1.5 Hz, H_b), 7.32-7.24 (m, 4 H, H_d, H_f), 7.22-7.14 (m, 6 H, H_c, H_e), 7.12(dd, 2 H, $^4J_{HP}$ = 2.9Hz, H_a), 6.98 (t, 1 H, H_g), 2.25 (s, 12 H, CH_3(Xyl)), 1.08 (d, 6 H, $^2J_{HP}$ = 8.0 Hz, PMe_2). All aromatic couplings are of *ca*. 7.5 Hz.

^{13}C{^{1}H} NMR (100 MHz, CD_2Cl_2, 25 ºC) δ: 171.7 (d, $^2J_{CP}$ = 122 Hz, C_5), 146.6 (d, $^2J_{CP}$ = 9 Hz, C_2), 142.0 (d, $^3J_{CP}$ = 3 Hz, C_3), 140.4 (CH_f), 137.3 (C_4), 131.6 (CH_b), 131.6 (d, $^3J_{CP}$ = 7 Hz, CH_a), 131.2 (d, $^1J_{CP}$ = 37 Hz, C_1), 128.9 (CH_d), 128.6 (CH_c), 127.8 (d, $^3J_{CP}$ = 7 Hz, CH_e), 125.7 (CH_g), 22.5 (CH_3(Xyl)), 17.0 (d, $^1J_{CP}$ = 31 Hz, PMe_2).

^{31}P{^{1}H} NMR (162 MHz, CD_2Cl_2, 25 ºC) δ: 17.1.

Compounds $[Au_2(\mu\text{-}CH_3)(PR_2Ar')_2]^+$ (4a-f)

$[Au_2(\mu\text{-}CH_3)(PMe_2Ar^{Mes2})_2][B(C_6F_5)_4]$ (4b)

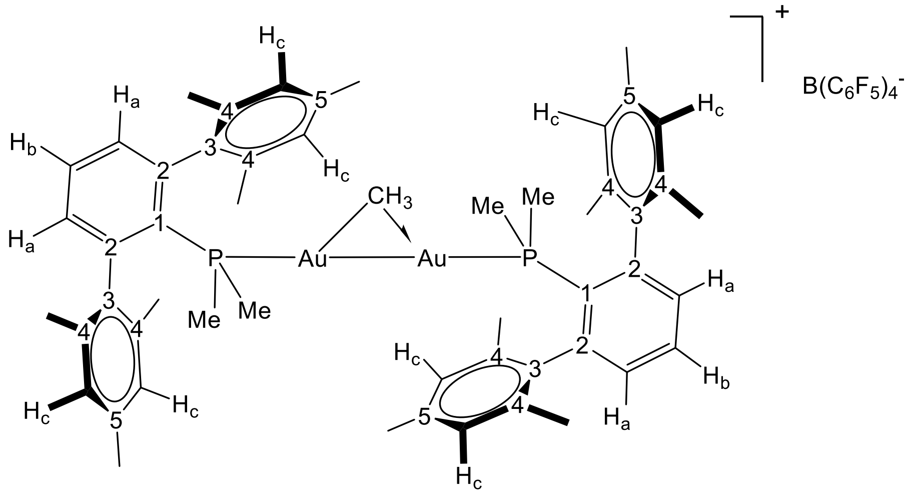

^{1}H NMR (400 MHz, CD_2Cl_2, -30 ºC) δ: 7.59 (td, 2 H, $^5J_{HP}$ = 1.4 Hz, H_b), 7.08 (dd, 4 H, $^4J_{HP}$ = 3.3 Hz, H_a), 6.91 (s, 8 H, H_c), 2.31 (s, 12 H, *p*-CH_3(Mes)), 1.91 (s, 24 H, *o*-CH_3(Mes)), 1.24 (d, 12 H, $^2J_{HP}$ = 9.8 Hz, PMe_2), 0.63 (s, 3 H, $AuCH_3\cdots Au$). All aromatic couplings are of *ca*. 7.5 Hz.

^{13}C{^{1}H} NMR (100 MHz, CD_2Cl_2, -30 ºC) δ: 147.4 (d, $^2J_{CP}$ = 12 Hz, C_2), 138.6 (C_5), 138.2 (d, $^4J_{CP}$ = 6 Hz, C_3), 136.5 (C_4), 133.2 (CH_b), 132.0 (d, $^4J_{CP}$ = 8 Hz, CH_a), 129.0 (CH_c), 126.7 (d, $^1J_{CP}$ = 54 Hz, C_1), 21.8 (*o*-CH_3(Mes)), 21.4 (*p*-CH_3(Mes)), 16.9 (d, $^1J_{CP}$ = 37 Hz, PMe_2), 1.2 (t, $^2J_{CP}$ = 54 Hz, $AuCH_3\cdots Au$).

^{31}P{^{1}H} NMR (162 MHz, CD_2Cl_2, 0 ºC) δ: 2.0.

[Au$_2$(µ-CH$_3$)(XPhos)$_2$][NTf$_2$] (4e)

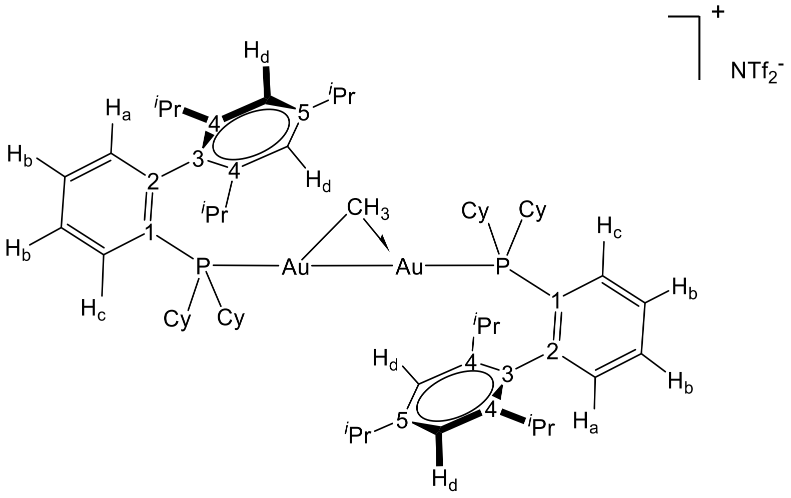

Anal. Calcd. for $C_{69}H_{101}Au_2F_6NO_4P_2S_2$: C, 50.5; H, 6.2; N, 0.9 **Found**: C, 50.3; H, 6.2; N, 0.9.

^{1}H NMR (500 MHz, CD_2Cl_2, 25 ºC) δ: 7.76 (m, 2 H, H_a), 7.64 (m, 4 H, H_b), 7.22 (m, 2 H, H_c), 7.09 (s, 4 H, H_d), 3.06 (hept, 2 H, $^3J_{HH}$ = 7.1 Hz, *o*-^{i}Pr(CH), 2.37 (hept, 4 H, $^3J_{HH}$ = 7.0 Hz, *p*-^{i}Pr(CH)), 2.15 (m, 2 H, Cy(CH$_2$)), 1.92 (m, 8 H, Cy(CH$_2$)), 1.85 (m, 2 H, Cy(CH)), 1.46 (m, 8 H, Cy(CH)), 1.44 (d, 12 H, $^3J_{HH}$ = 6.0 Hz, *p*-^{i}Pr(CH$_3$)), 1.37 (m, 8 H, Cy(CH)), 1.24 (m, 8 H, Cy(CH)), 1.26 (d, 12 H, $^3J_{HH}$ = 6.0 Hz, *o*-^{i}Pr(CH$_3$)), 1.03 (d, 6 H, $^3J_{HH}$ = 6.0 Hz, *o*-^{i}Pr(CH$_3$), c), 0.67 (t, 3 H, $^3J_{HP}$ = 2.2 Hz, AuCH$_3$···Au).

^{13}C{^{1}H} NMR (201 MHz, CD_2Cl_2, 25 ºC) δ: 150.3, 147.1, 146.7 (d, *J* = 14 Hz), 137.2 (d, *J* = 6 Hz), 134.2 (d, *J* = 10 Hz), 133.2, 131.1, 127.8 (d, *J* = 6 Hz), 127.5 (d, $^2J_{C\text{-}P}$ = 48 Hz), 121.3, 37.5 (d, *J* = 32 Hz), 34.2, 30.8 (d, *J* = 4 Hz), 30.8, 30.0 (d, *J* = 4 Hz), 26.8 (d, *J* = 12 Hz), 26.7 (d, *J* = 14 Hz), 25.7, 24.9, 24.2, 23.0, 3.1 (t, $^2J_{CP}$ = 48 Hz).

^{31}P{^{1}H} NMR (243 MHz, CD_2Cl_2, 25 ºC) δ: 39.5.

^{19}F{^{1}H} NMR (565 MHz, CD_2Cl_2, 25 ºC) δ: -79.7.

[Au$_2$(μ-CH$_3$)(^{t}BuXPhos)$_2$][NTf$_2$] (4f)

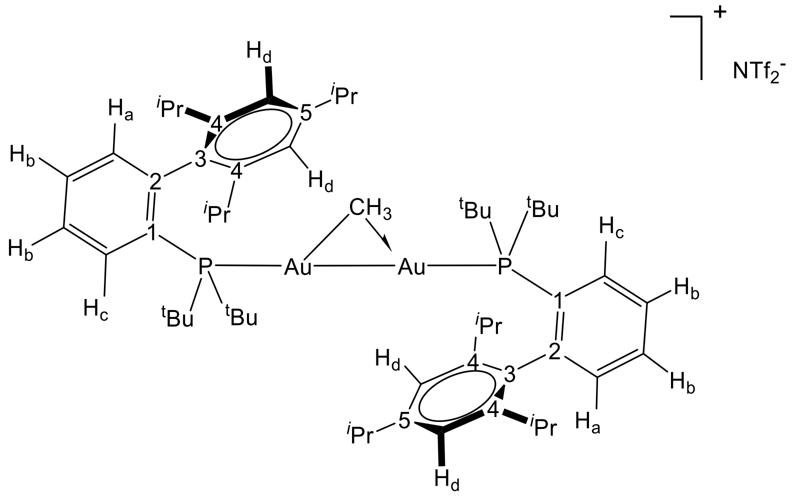

Anal. Calcd. for $C_{61}H_{93}Au_2F_6NO_4P_2S_2$: C, 47.6; H, 6.1; N, 0.9. **Found**: C, 47.9; H, 5.9; N, 0.9.

^{1}H NMR (500 MHz, CD_2Cl_2, 25 ºC) δ: 7.90 (m, 2H, H$_a$), 7.54 (m, 4 H , H$_b$), 7.15 (m, 2 H, H$_c$), 6.98 (s, 4 H, H$_d$), 2.91 (hept, 2 H, $^3J_{HH}$ = 6.8 Hz, *p*-^{i}Pr(CH)), 2.44 (hept, 4H, $^3J_{HH}$ = 6.4 Hz, *p*-^{i}Pr(CH)), 1.42 (d, 36 H, $^3J_{HP}$ = 18.0 Hz, ^{t}Bu), 1.26 (d, 6 H, $^3J_{HH}$ = 6.0 Hz, *o*-^{i}Pr(CH$_3$)), 1.16 (d, 6 H, $^3J_{HH}$ = 6.0 Hz, *p*-^{i}Pr(CH$_3$)), 0.88 (d, 6 H, $^3J_{HH}$ = 6.0 Hz, *o*-^{i}Pr(CH$_3$)), 0.87 (d, 3 H, $^3J_{HP}$ = 2.3 Hz, AuCH$_3$···Au).

^{13}C{^{1}H} NMR (201 MHz, CD_2Cl_2, 25 ºC) δ: 150.2, 147.5, 147.0 (d, $^1J_{C-P}$ = 14 Hz), 137.4(d, $^2J_{C-P}$ = 4 Hz), 135.2 (d, $^2J_{C-P}$ = 4 Hz), 135.1, 130.9, 128.7 (d, $^2J_{C-P}$ = 40 Hz), 127.1 (d, $^2J_{C-P}$ = 6.0 Hz), 121.7, 38.9 (d, $^1J_{C-P}$ = 24 Hz), 34.2, 31.3 (d, $^2J_{C-P}$ = 6 Hz), 30.7, 25.4, 24.0, 23.2, 2.7 (d, $^2J_{C-P}$ = 46 Hz, CH$_3$).

^{31}P{^{1}H} NMR (243 MHz, CD_2Cl_2, 25 ºC) δ: 66.8.

^{19}F{^{1}H} NMR (565 MHz, CD_2Cl_2, 25 ºC) δ: -79.6.

Compounds $[Au(PMe_2Ar')_2]^+$ (5)

For convenience, compounds **5** can be independently prepared by dissolving in dichloromethane (5 mL) an equimolar solid mixture of a halide gold precursor $AuCl(PMe_2Ar')$ (116 mg, 0.20 mmol) with the same PMe_2Ar' phosphine (69 mg, 0.20 mmol) in the presence of one equivalent of $AgNTf_2$ (78 mg, 0.20 mmol) or $NaBAr^F$ (117 mg, 0.20 mmol). The solution was stirred for 30 minutes and then filtrated through a celite bed. The volatiles were reduced under vacuum, and the residue washed with pentane to provide compounds **5** as white solids in *ca*. 90% yields.

$[Au(PMe_2Ar^{Xyl2})_2][BAr^F_4]$ (5a)

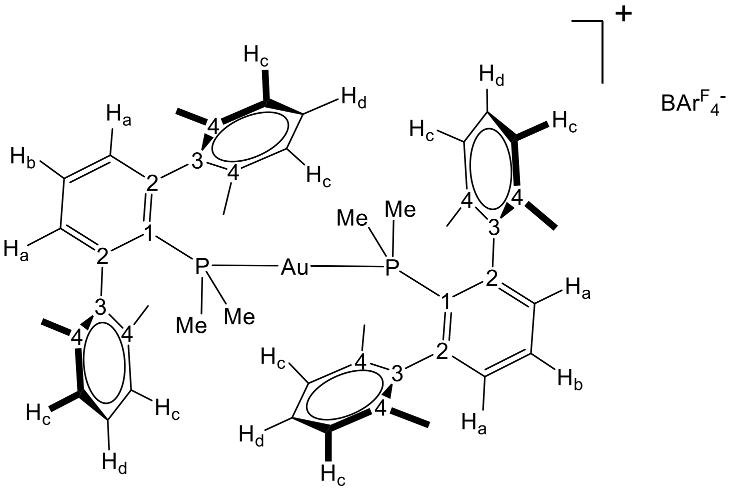

Anal. Calcd. for $C_{80}H_{66}AuBF_{24}P_2$: C, 54.8; H, 3.8. **Found**: C, 54.4; H, 3.9.

^{1}H NMR (400 MHz, CD_2Cl_2, 25 ºC) δ: 7.63 (t, 2 H, H_b), 7.23 (t, 4 H, H_d), 7.11 (d, 12 H, H_a, H_c), 2.03 (s, 24 H, CH_3(Xyl)), 1.05 (vt, 12 H, $^2J_{HP}$ = 3.7 Hz, PMe_2). All aromatic couplings are of *ca*. 7.5 Hz.

^{13}C{^{1}H} NMR (100 MHz, CD_2Cl_2, 25 ºC) δ: 146.8 (vt, $^2J_{CP}$ = 6 Hz, C_2), 140.9 (vt, $^4J_{CP}$ = 2 Hz, C_3), 136.9 (C_4), 133.5 (CH_b), 132.3 (vt, $^3J_{CP}$ = 4 Hz, CH_a), 129.6 (CH_d), 128.8 (CH_c), 126.7 (vt, $^1J_{CP}$ = 28 Hz, C_1), 22.3 (CH_3(Xyl)), 16.8 (vt, $^1J_{CP}$ = 18 Hz, PMe_2).

^{31}P{^{1}H} NMR (162 MHz, CD_2Cl_2, 25 ºC) δ: 10.6.

EM (ES) m/z Calcd. for M^+: 889.34. **Expt.**: 889.5.

[Au(PMe$_2$Ar^{Mes2})$_2$][BAr$^F_4$] (5b)

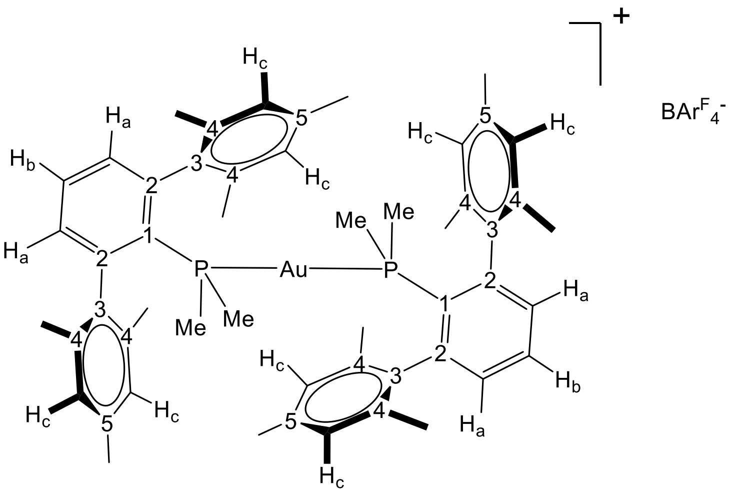

Anal. Calcd. for $C_{84}H_{74}AuBF_{24}P_2$: C, 55.8; H, 4.1. **Found**: C, 55.9; H, 4.1.

^{1}H NMR (500 MHz, CD_2Cl_2, 25 ºC) δ: 7.61 (t, 1 H, H$_b$), 7.08 (dd, 2 H, $^4J_{HP}$ = 3.5 Hz, H$_a$), 6.93 (s, 4 H, H$_c$), 2.30 (s, 12 H, *p*-CH$_3$(Mes)), 2.00 (s, 24 H, *o*-CH$_3$(Mes)), 1.06 (vt, 6 H, $^2J_{HP}$ = 3.7 Hz, PMe$_2$). All aromatic couplings are of *ca*. 7.5 Hz.

^{13}C{^{1}H} NMR (125 MHz, CD_2Cl_2, 25 ºC) δ: 146.9 (vt, $^2J_{CP}$ = 6 Hz, C$_2$), 139.2 (C$_5$), 138.2 (C$_3$), 136.8 (C$_4$), 133.3 (CH$_b$), 132.4 (vt, $^3J_{CP}$ = 4 Hz, CH$_a$), 129.5 (CH$_c$), 127.3 (vt, $^1J_{CP}$ = 29 Hz, C$_1$), 22.2 (*p*-CH$_3$(Mes)), 21.6 (*o*-CH$_3$(Mes)), 16.9 (vt, $^1J_{CP}$ = 18 Hz, PMe$_2$).

^{31}P{^{1}H} NMR (162 MHz, CD_2Cl_2, 25 ºC) δ: 11.5.

EM (ES) m/*z* Calcd. for M$^+$: 945.40. **Expt.**: 945.4.

[Au(PMe$_2$Ar^{Dipp2})$_2$][BAr$^F{}_4$] (5c)

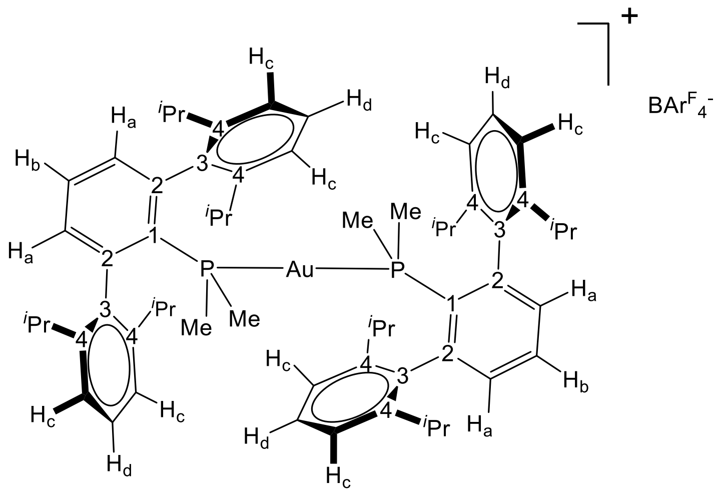

Anal. Calcd. for $C_{96}H_{98}AuBF_{24}P_2$: C, 58.3; H, 5.0. **Found**: C, 58.0; H, 4.7.

^{1}H NMR (500 MHz, CD_2Cl_2, 25 ºC) δ: 7.50 (t, 2 H, H$_b$), 7.38 (t, 4 H, H$_d$), 7.23 (d, 8 H, H$_c$), 7.07 (dd, 4 H, $^4J_{HP}$ = 4.0 Hz, H$_a$), 2.42 (hept, 8 H, $^3J_{HH}$ = 6.8 Hz, ^{i}Pr(CH)), 1.23 (d, 24 H, $^3J_{HH}$ = 6.9 Hz ^{i}Pr(CH$_3$)), 1.13 (vt, 12 H, $^2J_{HP}$ = 3.6 Hz, PMe$_2$), 0.99 (d, 24 H, $^3J_{HH}$ = 6.6 Hz, ^{i}Pr(CH$_3$)). All aromatic couplings are of *ca*. 7.5 Hz.

^{13}C{^{1}H} NMR (125 MHz, CD_2Cl_2, 25 ºC) δ: 147.6 (C$_4$), 146.1 (vt, $^2J_{CP}$ = 6 Hz, C$_2$), 139.3 (C$_3$), 134.2 (vt, $^3J_{CP}$ = 5 Hz, CH$_a$), 131.3 (CH$_b$), 130.5 (CH$_d$), 1228.1 (d, $^1J_{CP}$ = 40 Hz, C$_1$), 124.5 (CH$_c$), 32.1 (^{i}Pr(CH)), 25.6 (^{i}Pr(CH$_3$)), 23.4 (^{i}Pr(CH$_3$)), 16.7 (vt, $^1J_{CP}$ = 17 Hz, PMe$_2$).

^{31}P{^{1}H} NMR (200 MHz, CD_2Cl_2, 25 ºC) δ: 6.3.

EM (ES) m/*z* Calcd. for M$^+$: 1113.59. **Expt.**: 1113.7.

$[Au(PMe_2Ar^{Trip2})_2][B(C_6F_5)_4]$ (5d)

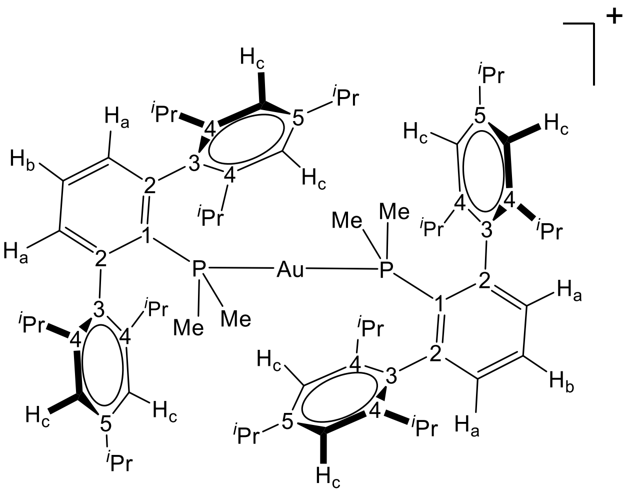

Anal. Calcd. for $C_{108}H_{122}AuBF_{24}P_2$: C, 60.5; H, 5.7. **Found**: C, 60.1; H, 5.3.

^{1}H NMR (400 MHz, CD_2Cl_2, 25 ºC) δ: 7.45 (t, 2 H, H_b), 7.05 (s, 8 H, H_c), 7.03 (vdt, 4 H, $^4J_{HP}$ = 2.0 Hz, H_a), 2.84 (hept, 4 H, $^3J_{HH}$ = 7.0 Hz, *p*-^{i}Pr(CH)), 2.39 (hept, 8 H, $^3J_{HH}$ = 6.8 Hz, *o*-^{i}Pr(CH)), 1.22 (d, 48 H, $^3J_{HH}$ = 6.9 Hz, *o,p*-^{i}Pr(CH_3)), 1.03 (vt, 12 H, $^2J_{HP}$ = 3.7 Hz, PMe_2), 0.97 (d, 24 H, $^3J_{HH}$ = 6.9 Hz, *o*-^{i}Pr(CH_3)). All aromatic couplings are of *ca*. 7.5 Hz.

^{13}C{^{1}H} NMR (100 MHz, CD_2Cl_2, 25 ºC) δ: 150.4 (C_5), 147.5 (C_4), 146.5 (vt, $^2J_{CP}$ = 7 Hz, C_2), 137.0 (vt, $^3J_{CP}$ = 3 Hz, C_3), 134.3 (vt, $^3J_{CP}$ = 4 Hz, CH_a), 131.2 (CH_b), 128.1 (vt, $^1J_{CP}$ = 31 Hz, C_1), 122.4 (CH_c), 35.0 (*p*-^{i}Pr(CH)), 32.1 (*o*-^{i}Pr(CH)), 25.6 (*o*-^{i}Pr(CH_3)), 24.4 (*o/p*-^{i}Pr(CH_3)), 23.3 (*o/p*-^{i}Pr(CH_3)), 16.8 (vt, $^1J_{CP}$ = 17 Hz, PMe_2).

^{31}P{^{1}H} NMR (162 MHz, CD_2Cl_2, 25 ºC) δ: 7.1.

EM (ES) m/*z* Calcd. for M^+: 1281.77. **Expt.**: 1282.0.

Compound $[Au_2(\mu\text{-}C_2H_5)(PMe_2Ar')_2][NTf_2]$ (6a)

A solid mixture of ethyl gold precursor **2a** (10 mg, 0.0175 mmol) with 1 equivalent of its parent compound $[Au(PMe_2Ar^{Xyl2})][NTf_2]$ (14 mg, 0.0175 mmol) were dissolved in CD_2Cl_2 (0.6 mL) under nitrogen at -50ºC to rapidly yield the desired ethyl-bridged complex **6a** in quantitative NMR spectroscopic yield. Characterization of compound **6a** was carried out by multinuclear NMR spectroscopy at low temperature without further purification. Alternatively, **6a** can be prepared in comparable yield by treating compounds **2a** (20 mg, 0.035 mmol) with half equivalent of $[Ph_3C][B(C_6F_5)_4]$ (16 mg, 0.0175 mmol) in dichloromethane under otherwise identical conditions.

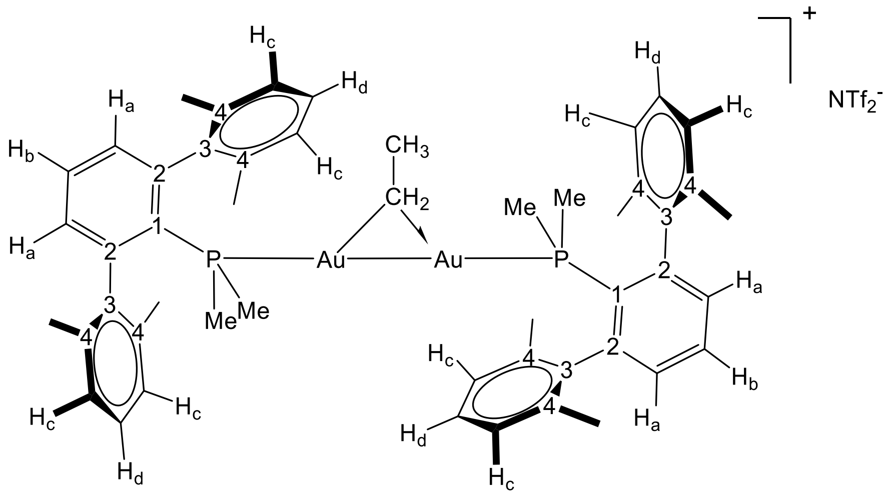

^{1}H NMR (400 MHz, CD_2Cl_2, -65 ºC) δ: 7.62 (br t, 2 H, H_b), 7.26 (t, 4 H, H_d), 7.16 -6.96 (m, 12 H, H_a, H_c), 1.94 (s, 24 H, CH_3(Xyl)), 1.38 (m, 3 H, AuCH_2CH$_3$...Au), 1.18 (d, 12 H, $^2J_{HP}$ = 7.8 Hz, PMe_2), 0.96 (m, 3 H, AuCH$_2$$CH_3$...Au). All aromatic couplings are of *ca*. 7.5 Hz.

^{13}C{^{1}H} NMR (100 MHz, CD_2Cl_2, -65 ºC) δ: 146.5 (d, $^2J_{CP}$ = 13 Hz, C_2) , 140.5 (C_3), 136.3 (C_4), 133.0 (CH_b), 131.1 (CH_a), 128.2 (CH_d), 127.5 (CH_c), 125.7 (d, $^1J_{CP}$ = 52 Hz, C_1), 21.8 (CH_3(Xyl)), 16.2 (d, $^1J_{CP}$ = 37 Hz, PMe_2), 20.8 (d, $^1J_{CP}$ = 131 Hz, AuCH_2CH$_3$···Au), 15.7 (AuCH$_2$$CH_3$···Au).

^{31}P{^{1}H} NMR (162 MHz, CD_2Cl_2, -65 ºC) δ: 2.08.

Compound $[Au_2(\mu\text{-}C_6H_5)(PMe_2Ar')_2][NTf_2]$ (7a)

A solid mixture of ethyl gold precursor **3a** (11 mg, 0.0175 mmol) with 1 equivalent of its parent compound $[Au(PMe_2Ar^{Xyl2})][NTf_2]$ (14 mg, 0.0175 mmol) were dissolved in CD_2Cl_2 (0.6 mL) under nitrogen at -50ºC to rapidly yield the desired phenyl-bridged complex **7a** in quantitative NMR spectroscopic yield. Characterization of compound **7a** was carried out by multinuclear NMR spectroscopy at low temperature without further purification. Alternatively, **7a** can be prepared in comparable yield by treating compounds **3a** (22 mg, 0.035 mmol) with half equivalent of $[Ph_3C][B(C_6F_5)_4]$ (16 mg, 0.0175 mmol) in dichloromethane under otherwise identical conditions.

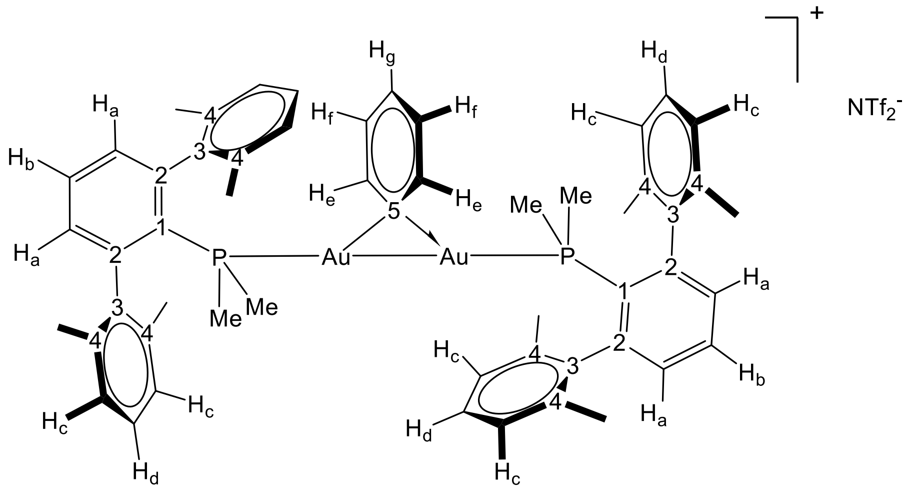

^{1}H NMR (400 MHz, CD_2Cl_2, -20 ºC) δ: 7.58 (t, 3 H, $^5J_{HP}$ = 1.6 Hz (H_b), H_b, H_g), 7.50 (t, 2 H, H_f), 7.42 (d, 2 H, H_e), 7.09 (t, 4 H, H_d), 7.04 (dd, 4 H, $^4J_{HP}$ = 3.3 Hz, H_a), 6.96 (d, 8 H, H_c), 1.92 (s, 24 H, CH_3(Xyl)), 1.01 (d, 12 H, $^2J_{HP}$ = 9.7 Hz, PMe_2). All aromatic couplings are of *ca*. 7.5 Hz.

^{13}C{^{1}H} NMR (100 MHz, CD_2Cl_2, -35 ºC) δ: 150.2 (CH_e), 146.0 (d, $^2J_{CP}$ = 10 Hz, C_2), 140.5 (C_{arom}), 136.7 (d, J_{CP} = 27 Hz, C_{arom}), 136.4 (C_{arom}), 132.6 (CH_b), 131.3 (d, $^3J_{CP}$ = 8 Hz, CH_a), 128.8 (CH_d), 128.8 (d, J_{CP} = 34 Hz, C_{arom}), 128.2 (CH_f), 128.0 (CH_c), 126.3 (d, $^1J_{CP}$ = 52 Hz, C_1), 22.0 (CH_3(Xyl)), 16.8 (d, $^1J_{CP}$ = 37 Hz, PMe_2).

^{31}P{^{1}H} NMR (162 MHz, CD_2Cl_2, -20 ºC) δ: 3.1.

II. X-Ray Structural Characterization of new compounds.

A suitable single crystal of **1a, 1e, 4e, 1f, 4f, 2a, 3a** or Au(^{t}BuXPhos)([NTf_2) was coated with Paratone oil and mounted on a MiTeGen MicroLoop. The X-ray intensity data were measured on a Bruker Kappa APEXII Duo system (**1e**, **4e**, **1f**, **4f**, [Au^{t}BuXPhos][NTf_2]), a Bruker D8 Quest APEX-III single crystal diffractometer with a Photon III detector and a IμS 3.0 microfocus X-ray source (**1a**, **2a** and **3a**). The frames were integrated with the Bruker SAINT software package using a narrow-frame algorithm. Data were corrected for absorption effects using the Multi-Scan method (SADABS). The structure was solved and refined using the Bruker SHELXTL Software Package[2] within APEX3[3] and OLEX2.[4] Non-hydrogen atoms were refined anisotropically. Hydrogen atoms were placed in geometrically calculated positions with U_{iso} = 1.2U_{equiv} of the parent atom (U_{iso} = 1.5U_{equiv} for methyl), except for the hydrogen atoms of the bridging methyls in 4a and 5a. Those were located in the electron density map and refined isotropically with restraints on their bonds.

In **4e**, the relative occupancies of the disordered gold atoms was freely refined, with constraints on the anistropic displacement parameters of the disordered atoms. In **1f**, one isopropyl substituent was found to be disordered over two positions. The relative occupancies were allowed to refine freely, with constraints on the anisotropic displacement parameters of the disordered atoms. In **2a**, one ethyl group was disordered over two positions. The relative occupancies were allowed to refine freely, with constraints on the anisotropic displacement parameters of the disordered atoms and restraints on the disordered bonds.

A summary of all crystallographic data and refinement parameters for each compound is provided in Tables S1 and S2. Atomic coordinates, anisotropic displacement parameters and bond lengths and angles can be found in the cif files which have been deposited in the Cambridge Crystallographic Data Centre with no. 2024182-2024189. These data can be obtained free of charge from The Cambridge Crystallographic Data Centre via www.ccdc.cam.ac.uk/data_request/cif.

Table S1. Crystal data table for **1a**, **1e**, **4e** and **1f**.

	**1a**	**1e**	**4e**	**1f**
**CCDC**	2024182	2024183	2024187	2024184
**Chemical formula**	$C_{25}H_{30}AuP$	$C_{34}H_{52}AuP$	$C_{69}H_{101}Au_2F_6NO_4P_2S_2$	$C_{30}H_{48}AuP$
**FW (g/mol)**	558.42	688.69	1642.49	636.62
**T (K)**	193.0	100(2)	100(2)	100(2)
**λ (Å)**	0.71073	0.71073	0.71073	0.71073
**Crystal size (mm)**	0.20 x 0.18 x 0.12	0.152 x 0.240 x 0.325	0.238 x 0.379 x 0.478	0.224 x 0.248 x 0.275
**Crystal habit**	colorless block	colorless block	colorless block	colorless block
**Crystal system**	orthorhombic	monoclinic	triclinic	monoclinic
**Space group**	Pbca	C 2/c	P 1	P c
**a (Å)**	13.8037(8)	40.915(2)	15.0694(16)	18.9574(17)
**b (Å)**	18.0029(11)	8.9350(5)	16.8288(18)	8.4438(8)
**c (Å)**	18.0091(10)	17.4189(9)	22.109(3)	19.3004(17)
**α (°)**	90	90	104.309(3)	90
**β (°)**	90	97.5600(10)	98.936(3)	111.859(2)
**γ (°)**	90	90	95.867(3)	90
**V ($Å^3$)**	4475.4(5)	6312.6(6)	5308.7(10)	2867.3(5)
**Z**	8	8	3	4
**ρ_{calc} ($g/cm^{3)}$**	1.658	1.449	1.541	1.475
**μ (mm^{-1})**	6.653	4.731	4.306	5.201
**F(000)**	2192.0	2800	2484	1288
**θ range (°)**	2.18 to 28.30	2.01 to 30.54	1.38 to 30.61	2.13 to 30.55
**Index ranges**	-18 ≤ h ≤ 18<br>-23 ≤ k ≤ 23<br>-21 ≤ h ≤ 24	-42 ≤ h ≤ 58<br>-12 ≤ k ≤ 12<br>-24 ≤ h ≤ 24	-21 ≤ h ≤ 21<br>-24 ≤ k ≤ 24<br>-31 ≤ h ≤ 31	-27 ≤ h ≤ 27<br>-12 ≤ k ≤ 12<br>-27 ≤ h ≤ 27
**Reflns coll.**	95964	40992	129263	64529
**Ind. reflns**	5544 [R_{int} = 0.0366	9646 [R_{int} = 0.0241]	63746 [R_{int} = 0.0333]	17476 [R_{int} = 0.0345]
**Data / restraints / parameters**	5544/0/251	9646 / 0 / 332	63746 / 25 / 2370	17476 / 2 / 616
**Goodness-of-fit on F^2**	1.093	1.051	0.864	1.030
**R_1 [I>2σ(I)]**	0.0203	0.0166	0.0312	0.0222
**wR_2 [all data]**	0.0529	0.0375	0.0644	0.0434

Table S2. Crystal data table for **4f**, **2a**, **3a** and Au(^{t}BuXPhos)(NTf$_2$).

	**4f**	**2a**	**3a**	**Au(^{t}BuXPhos)(NTf$_2$)**
**CCDC**	2024188	2024185	2024186	2024189
**Chemical formula**	$C_{62}H_{95}Au_2Cl_2F_6NO_4P_2S_2$	$C_{54}H_{68}Au_2P_2$	$C_{30}H_{32}AuP$	$C_{31}H_{45}AuF_6NO_4PS_2$
**FW (g/mol)**	1623.28	1172.95	620.49	901.64
**T (K)**	100(2)	193(2)	193(2)	100(2)
**λ (Å)**	0.71073	0.71073	0.71073	0.71073
**Crystal size (mm)**	0.110 x 0.220 x 0.302	0.18 x 0.16 x 0.11	0.13 x 0.10 x 0.10	0.270 x 0.390 x 0.404
**Crystal habit**	colorless plate	colorless block	colorless block	colorless block
**Crystal system**	orthorhombic	monoclinic	monoclinic	triclinic
**Space group**	P na2$_1$	P2$_1$/c	P2$_1$/c	P -1
**a (Å)**	20.9449(16)	14.2800(9)	13.316(2)	9.2607(11)
**b (Å)**	24.264(2)	18.6671(12)	11.617(2)	11.2123(13)
**c (Å)**	13.1754(10)	18.2973(11)	17.075(3)	18.925(2)
**α (°)**	90	90	90	104.530(3)
**β (°)**	90	91.008(3)	102.132(11)	96.111(3)
**γ (°)**	90	90	90	105.683(3)
**V (Å^3)**	6695.8(9)	4876.7(5)	2582.4(8)	1799.1(4)
**Z**	4	4	4	2
**ρ_{calc} (g/cm$^{3)}$**	1.610	1.598	1.596	1.664
**μ (mm^{-1})**	4.628	6.109	5.774	4.315
**F(000)**	3256	2320	1224	900
**θ range (°)**	1.28 to 26.46	2.10 to 27.54	2.14 to 27.49	1.13 to 30.57
**Index ranges**	-25 ≤ h ≤ 26<br>-30 ≤ k ≤ 30<br>-16 ≤ h ≤ 16	-18 ≤ h ≤ 18<br>-24 ≤ k ≤ 24<br>-23 ≤ h ≤ 20	-16 ≤ h ≤ 17<br>-15 ≤ k ≤ 15<br>-22 ≤ h ≤ 22	-13 ≤ h ≤ 13<br>-16 ≤ k ≤ 16<br>-27 ≤ h ≤ 27
**Reflns coll.**	60632	60230	39055	84256
**Ind. reflns**	13651 [R_{int} = 0.0470]	11214 [R_{int} = 0.0408	5914 [R_{int} = 0.0752	11015 [R_{int} = 0.0289]
**Data / restraints / parameters**	13651 / 7 / 766	11214 / 1 / 545	5914 / 0 / 295	11015 / 1 / 436
**Goodness-of-fit on F^2**	1.071	1.027	1.097	1.114
**R$_1$ [I>2σ(I)]**	0.0323	0.0456	0.0448	0.0134
**wR$_2$ [all data]**	0.0697	0.1242	0.1292	0.0338

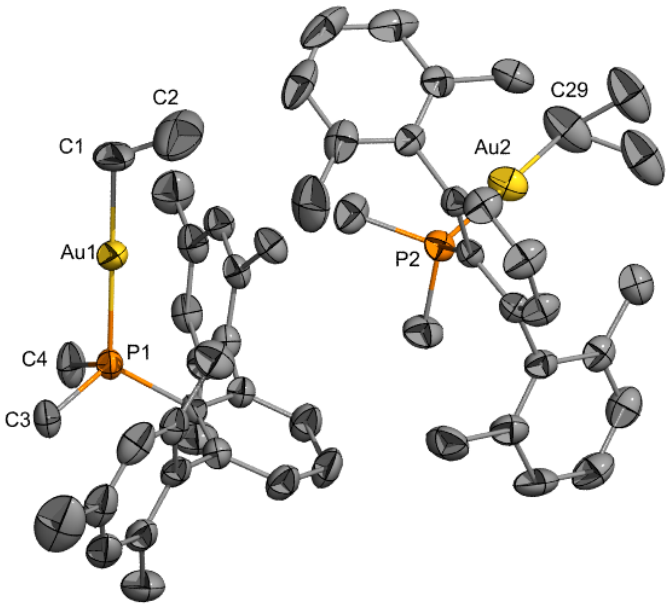

Figure S1. ORTEP structure of co-crystallized $[Au(C_2H_5)(PMe_2Ar^{Xyl2})]$ (**2a**), and $[Au(C_2H_5)(PMe_2Ar^{Mes2})]$ (**2b**) at 50% probability. Hydrogen atoms have been omitted for clarity.

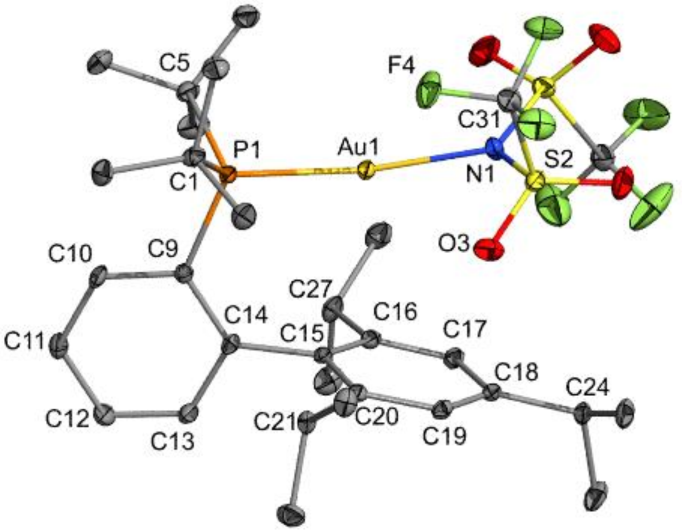

Figure S2. ORTEP structure of $Au(^tBuXPhos)(NTf_2)$ represented at 50% probability and where hydrogen atoms have been omitted for clarity. Selected bond lengths (Å): Au1–C15 = 3.1904(2); Au1–C16 = 3.3652(2); Au1–C20 = 3.6406(2); Au···arene centroid = 3.4804(9); Au1–N1 = 2.132(2); Au1–P1 = 2.250(3); Selected bond angles (°): P1–Au1–N1 = 170.05(5); C9–P1–Au1 = 114.70(6).

III. Kinetic studies

Kinetic studies were performed to determine the order on bridging digold methyl compounds (**4**) during C-C coupling processes. Ethane evolution and the disappearance of compounds **4** has been monitored at several temperatures by ^{1}H and $^{31}P\{^{1}H\}$ NMR spectroscopy as reported in the Experimental Section of the main text. Table S3 collects the average data obtained for triplicates run for each experiment. Figures S3-S5 represent selected examples of the second-order kinetic plots from which data were acquired. Figure S6 depicts the corresponding kinetic plot for our preliminary experiments run under the same conditions but between an equimolar mixture of $Au(CH_3)(PPh_3)$ and $Au(PPh_3)(NO_3)$.

Table S3. Summary of kinetic data for C-C bond formation from compounds **4**.

**Compound**	T (ºC)	k ($M^{-1}\cdot s^{1}$)	ΔG‡ (kcal mol^{-1})
$[Au_2(\mu\text{-}CH_3)(PMe_2Ar^{Xyl2})_2][NTf_2]$ (**4a**)	10	2.9(4) x 10^{-1}	17.2 ± 0.8 (10ºC)
$[Au_2(\mu\text{-}CH_3)(PMe_2Ar^{Xyl2})_2][NTf_2]$ (**4a**)	5	2.23(9) x 10^{-1}	17.1 ± 0.1 (5ºC)
$[Au_2(\mu\text{-}CH_3)(PMe_2Ar^{Xyl2})_2][NTf_2]$ (**4a**)	0	9.8(4) x 10^{-2}	17.2 ± 0.1 (0ºC)
$[Au_2(\mu\text{-}CH_3)(PMe_2Ar^{Xyl2})_2][NTf_2]$ (**4a**)	-5	4.2(1) x 10^{-2}	17.3 ± 0.2 (-5ºC)
$[Au_2(\mu\text{-}CH_3)(PMe_2Ar^{Xyl2})_2][NTf_2]$ (**4a**)	-10	2.24(5) x 10^{-2}	17.3 ± 0.2 (-10ºC)
$[Au_2(\mu\text{-}CH_3)(PMe_2Ar^{Xyl2})_2][NTf_2]$ (**4a**)	-20	4.8(1) x 10^{-4}	17.4 ± 0.1 (-20ºC)
$[Au_2(\mu\text{-}CH_3)(PMe_2Ar^{Mes2})_2][B(C_6F_5)_4]$ (**4b**)	0	4.9(1) x 10^{-2}	17.6 ± 0.1 (0ºC)
$[Au_2(\mu\text{-}CH_3)(PMe_2Ar^{Dipp2})_2][NTf_2]$ (**4c**)	50	4.8(3) x 10^{-3}	22.4 ± 0.5 (50ºC)
$[Au_2(\mu\text{-}CH_3)(PMe_2Ar^{Tipp2})_2][B(C_6F_5)_4]$ (**4d**)	50	2.0(1) x 10^{-3}	22.9 ± 0.4 (50ºC)
$[Au_2(\mu\text{-}CH_3)(XPhos)_2][NTf_2]$ (**4e**)	90	5.2(1) x 10^{-4}	26.4 (90ºC)

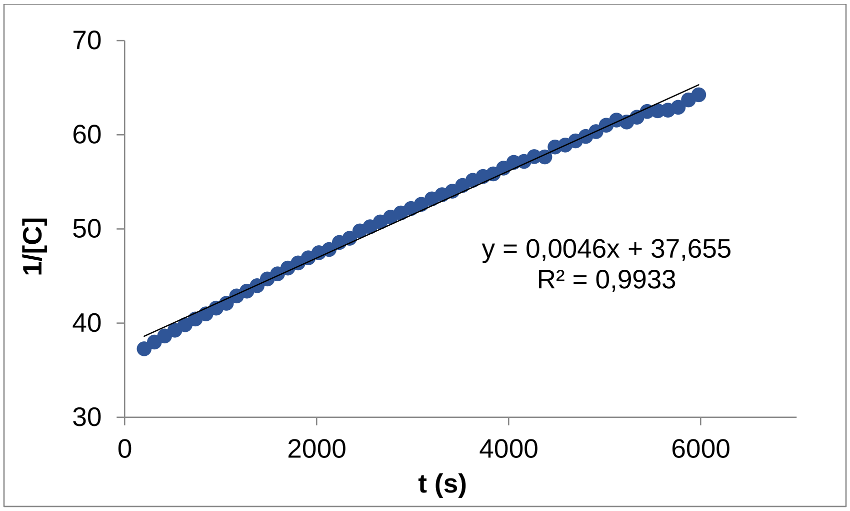

Figure S3. Selected representative second-order kinetic plot for ethane evolution from $[Au_2(\mu\text{-}CH_3)(PMe_2Ar^{Xyl2})_2][NTf_2]$ (**4a**) in CD_2Cl_2 at -20ºC.

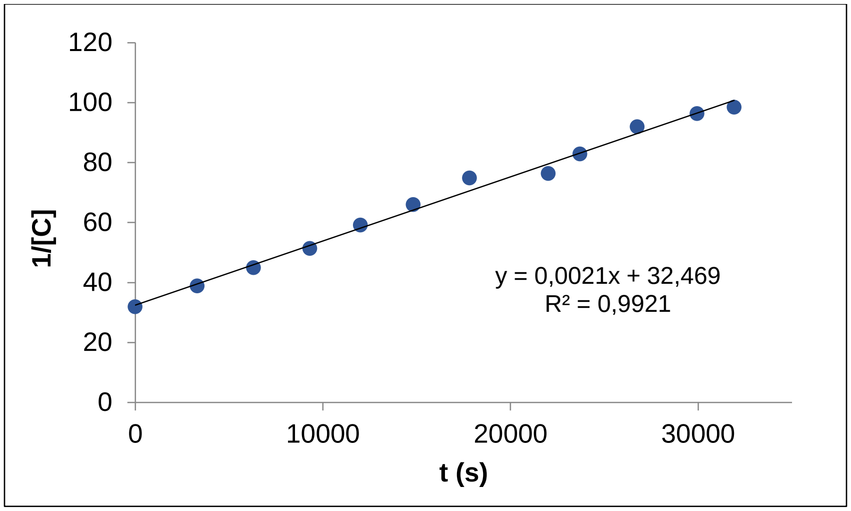

Figure S4. Selected representative second-order kinetic plot for ethane evolution from [Au_2(*μ*-CH_3)(PMe_2Ar^{Tipp2})$_2$][NTf_2] (**4d**) in CD_2Cl_2 at 50ºC.

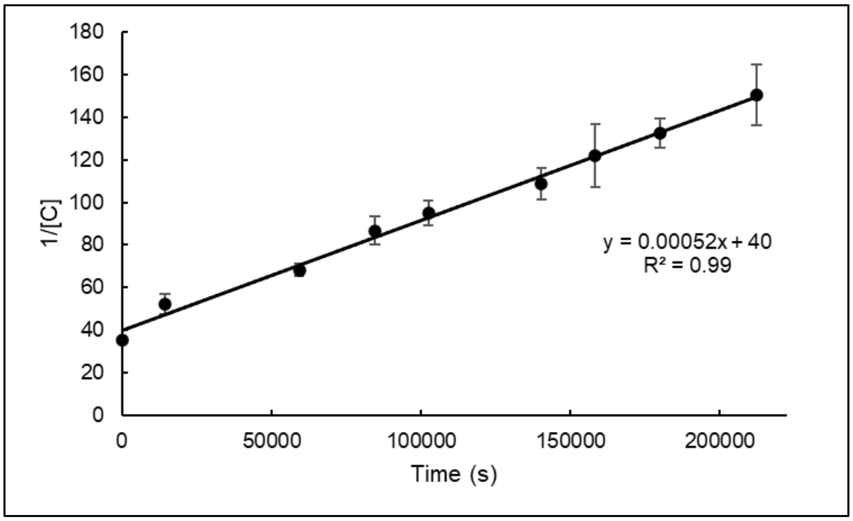

Figure S5. Selected representative second-order kinetic plot for ethane evolution from [Au_2(*μ*-CH_3)(XPhos)$_2$][NTf_2] (**4e**) in CD_2ClCD_2Cl at 90ºC.

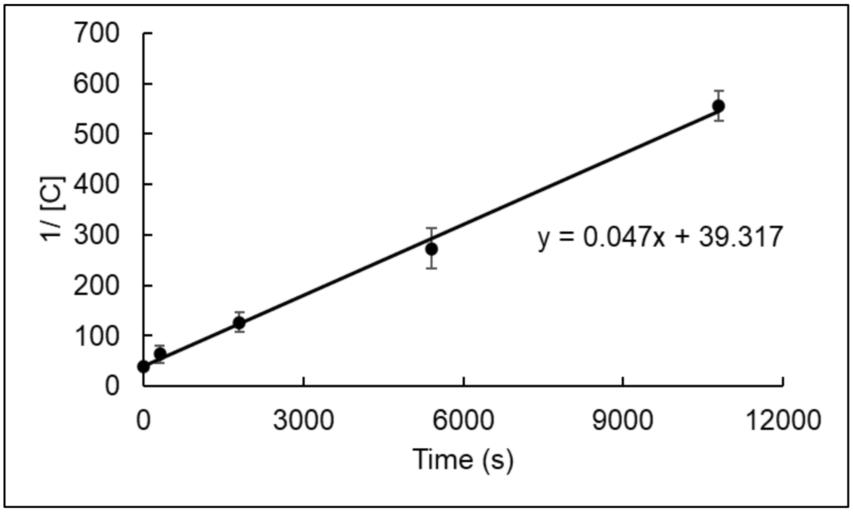

Figure S6. Selected representative second-order kinetic plot for ethane evolution from the equimolar reaction between $Au(CH_3)(PPh_3)$ and $Au(PPh_3)(NO_3)$ in CD_2Cl_2 at 25 ºC.

Effect of BPh_3. To examine the effect of BPh_3 on the rate of ethane evolution under pseudofirstorder conditions a similar procedure to that employed for other kinetic experiments was followed. An equimolar solid mixture of **1a** (2 mg, 0.0038 mmol) and $Au(PMe_2Ar^{Xyl2})(NTf_2)$ (3 mg, 0.0038 mmol) was placed in a J-Young NMR tube. Excess BPh_3 (5 mg, 0.019 mmol) was added under nitrogen and the mixture dissolved in CD_2Cl_2 at -40ºC. The reaction was monitored at -10 ºC by ^1H and $^{31}P\{^1H\}$ NMR spectroscopy, resulting in a measured pseudo-first order kinetic constant of -8.1(2) x 10^{-4} s^{-1}. Figure S7 evinces the first-order dependence on **4a** in the presence of BPh_3.

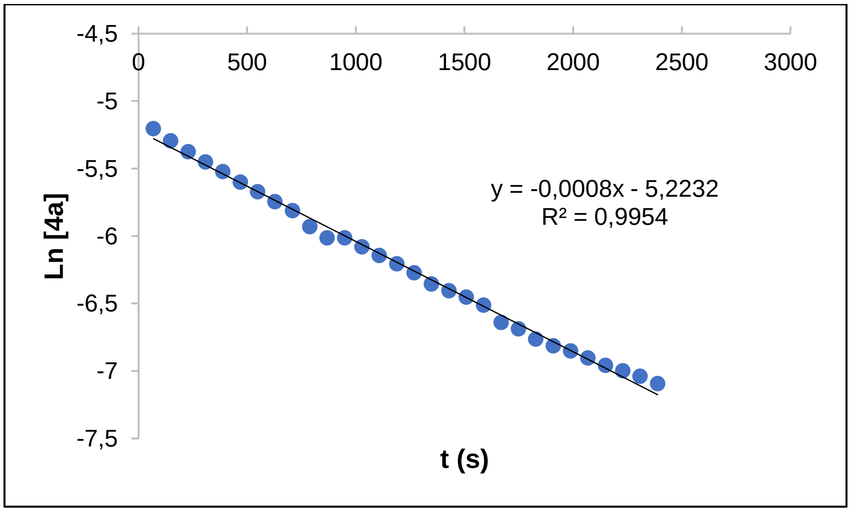

Figure S7. One of the representative pseudofirst order kinetic experiments performed with $[Au_2(\mu\text{-}CH_3)(PMe_2Ar^{Xyl2})_2][NTf_2]$ (**4a**) at -10 ºC with 5 equivalents of BPh_3.

IV. Variable temperature studies and exchange reactions.

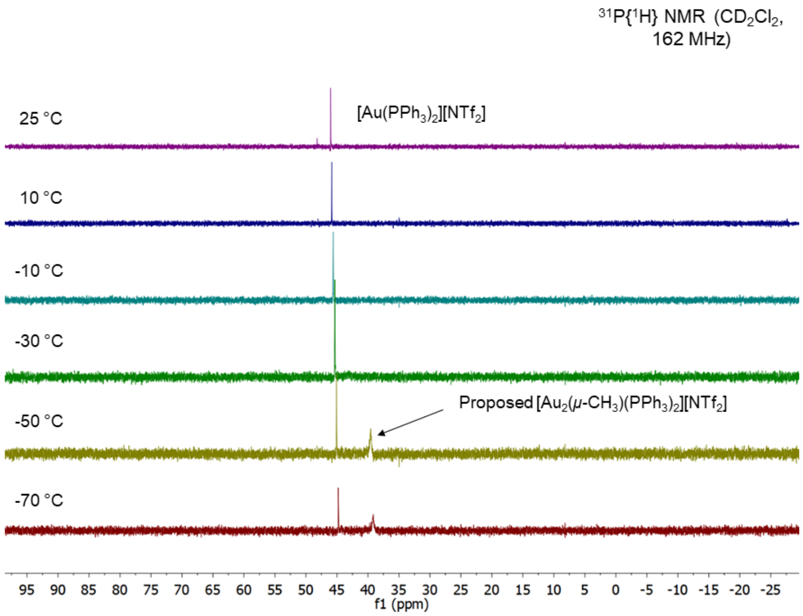

Figure S8. Variable temperature $^{31}P\{^{1}H\}$ NMR monitoring of the reaction between an equimolar mixture of $Au(CH_3)(PPh_3)$ and $Au(PPh_3)(NTf_2)$ to yield $[Au(PPh_3)_2][NTf_2]$, Au(0) and C_2H_6.

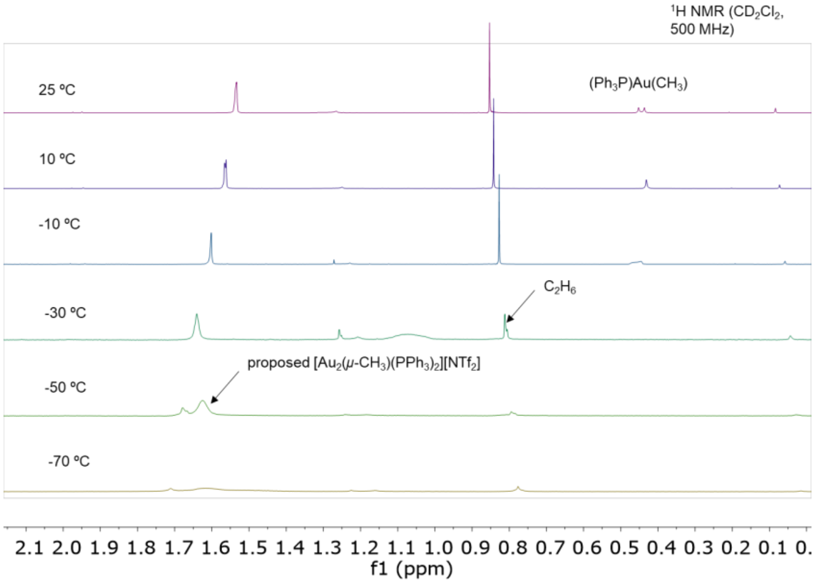

Figure S9. Variable temperature ^{1}H NMR monitoring of the reaction between an equimolar mixture of $Au(CH_3)(PPh_3)$ and $Au(PPh_3)(NTf_2)$ to yield $[Au(PPh_3)_2][NTf_2]$, Au(0) and C_2H_6.

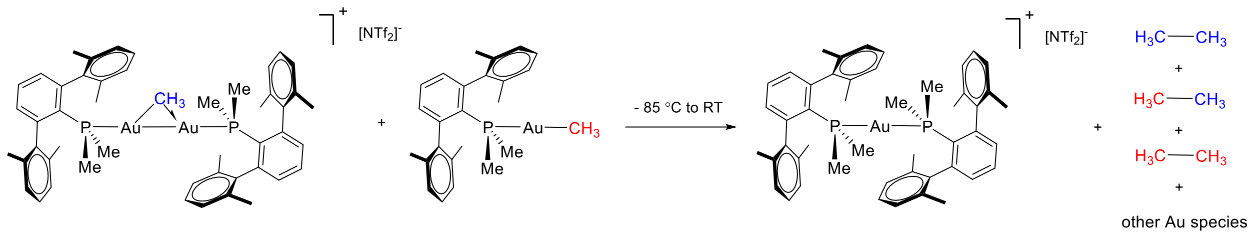

4a **1a** **5a**

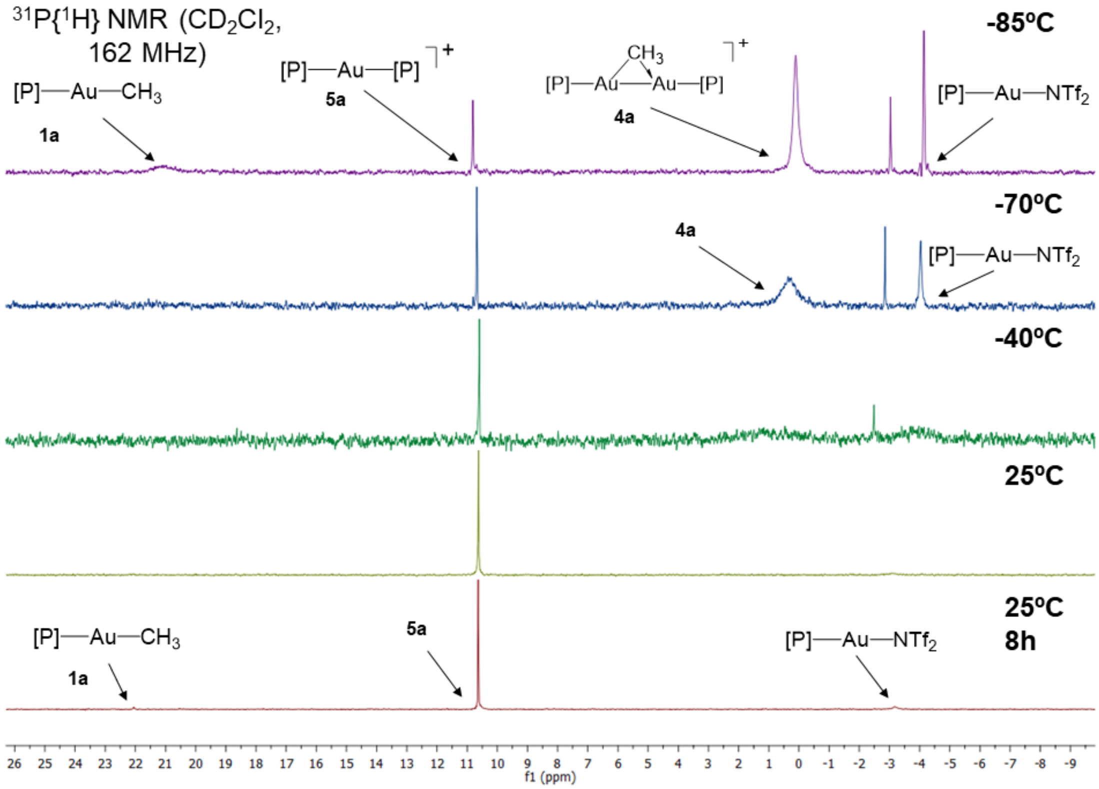

Figure S10. Variable temperature $^{31}P\{^1H\}$ NMR monitoring of the reaction between $Au(CH_3)(PMe_2Ar^{Xyl2})$ (**1a**) (0.012 mmol) and $[Au_2(\mu\text{-}CH_3)(PMe_2Ar^{Xyl2})_2][NTf_2]$ (**4a**) (0.012 mmol) in CD_2Cl_2 evidencing rapid dynamic exchange (coalescence at around -40 ºC).

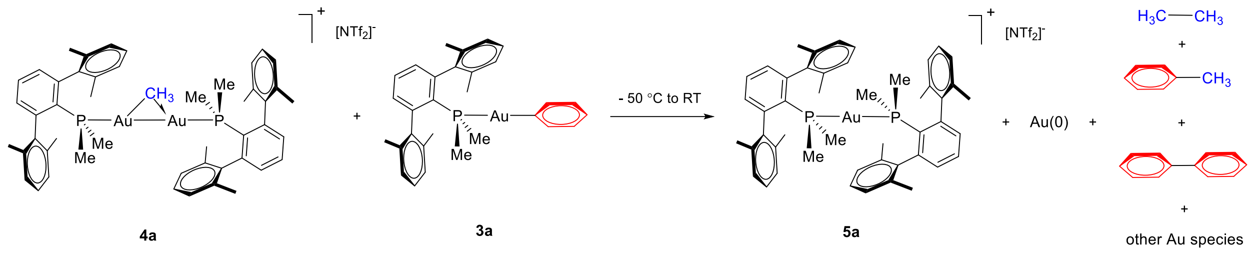

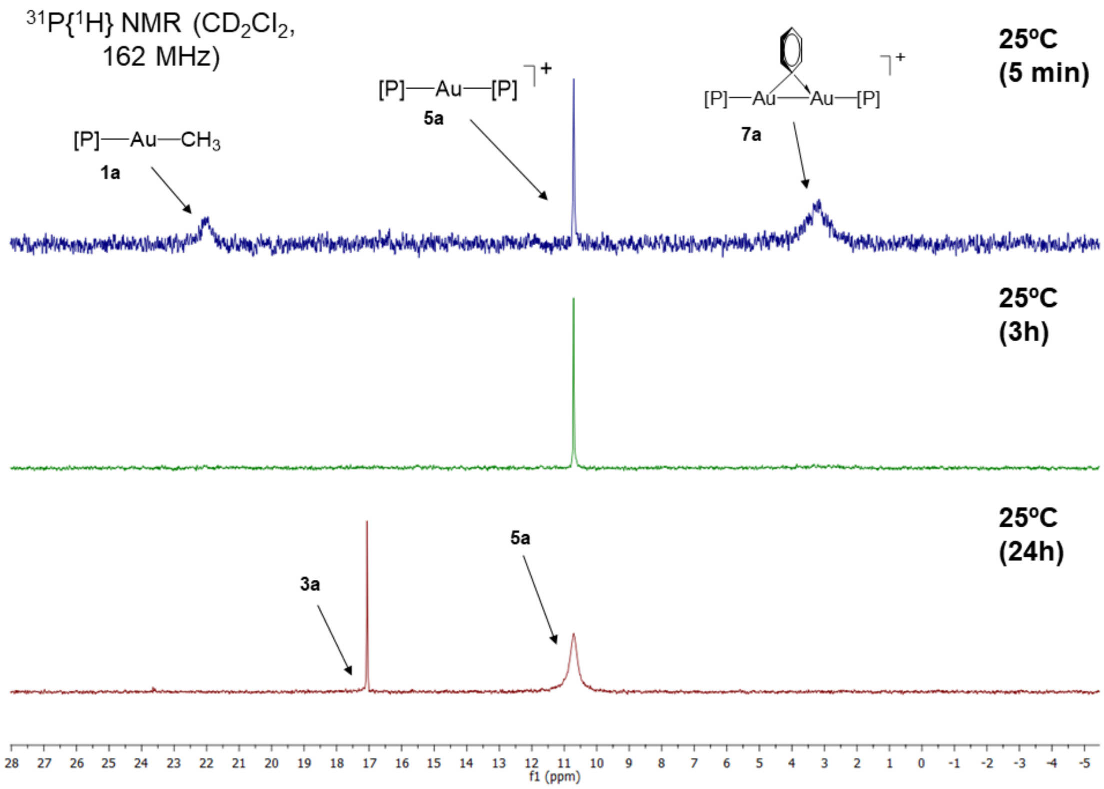

Figure S11. $^{31}P\{^1H\}$ NMR monitoring of the reaction between $[Au_2(\mu\text{-}CH_3)(PMe_2Ar^{Xyl2})_2][NTf_2]$ (**4a**) (0.012 mmol) and $Au(C_6H_5)(PMe_2Ar^{Xyl2})$ (**3a**) (0.012 mmol) in CD_2Cl_2 at 25 ºC.

Isotopic labelling (CH_3/CD_3) experiment. Analogously to the exchange experiments detailed above, a solid mixture of **1a** (0.011 mmol), **1a-*d*$_3$** (0.011 mmol) and $Au(PMe_2Ar^{Xyl2})(NTf_2)$ (0.022 mmol) were placed in a screwcap sealed NMR tube which was cooled to -20ºC. CD_2Cl_2 was added slowly and the tube shaken at the same temperature for a few minutes, then the mixture was warmed up to room temperature and analyzed spectroscopically. Figure S12 shows a distinctive pattern for a 2:1 mixture of CD_3CH_3 (0.83 ppm) and C_2H_6 (0.85 ppm).

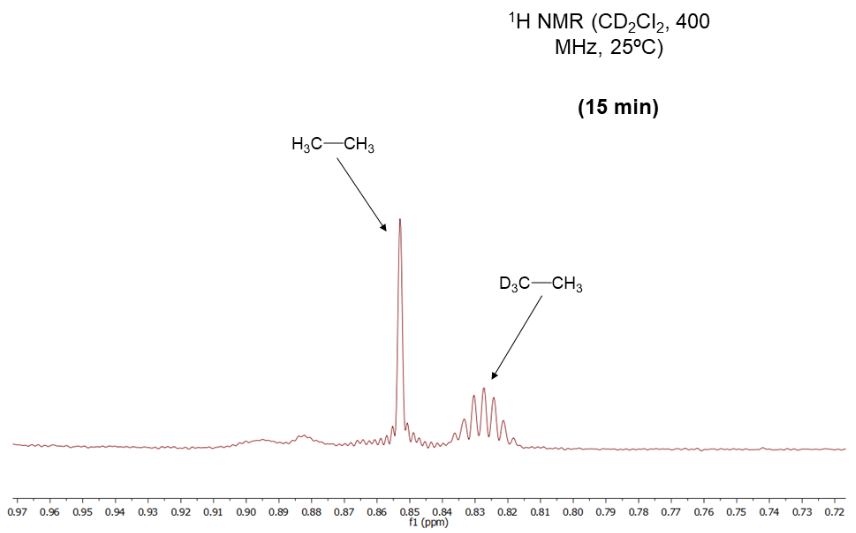

Figure S12. ^{1}H NMR monitoring of the reaction between $Au(CH_3)(PMe_2Ar^{Xyl2})$ (**1a**) (0.011 mmol), $Au(CD_3)(PMe_2Ar^{Xyl2})$ (**1a-*d*$_3$**) (0.011 mmol) and $Au(PMe_2Ar^{Xyl2})(NTf_2)$ (0.022 mmol) in CD_2Cl_2 at 25 ºC.

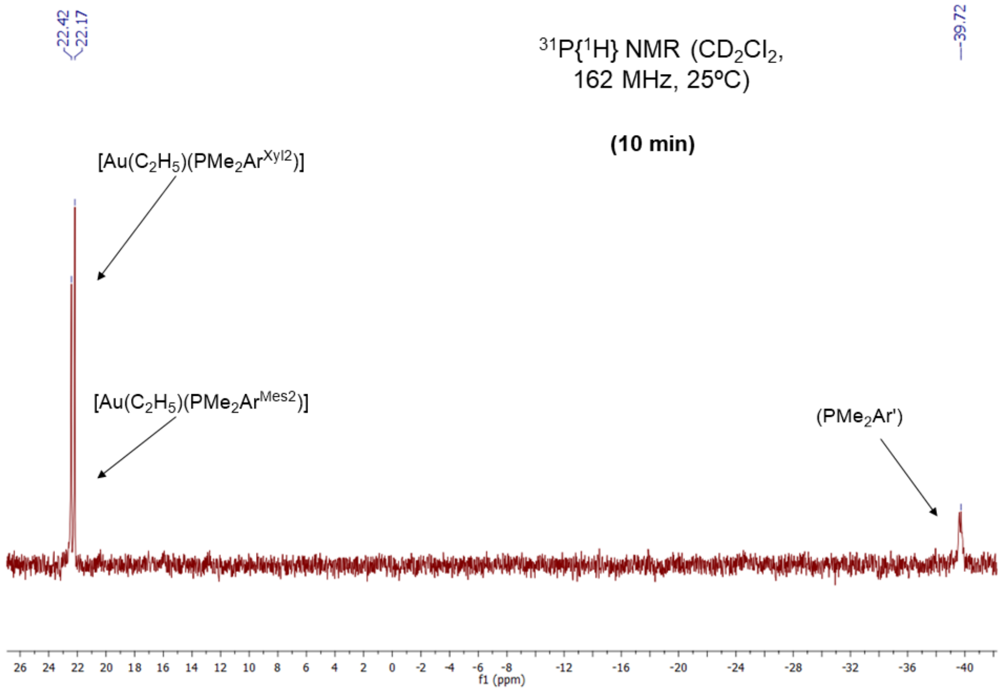

Figure S13. ^{31}P{^{1}H} NMR spectrum resulting from the exchange reaction between $Au(C_2H_5)(PMe_2Ar^{Xyl2})$ (**2a**) (0.0175 mmol) and PMe_2Ar^{Mes2} (0.0175 mmol) in CD_2Cl_2 at 25 ºC after 10 minutes.

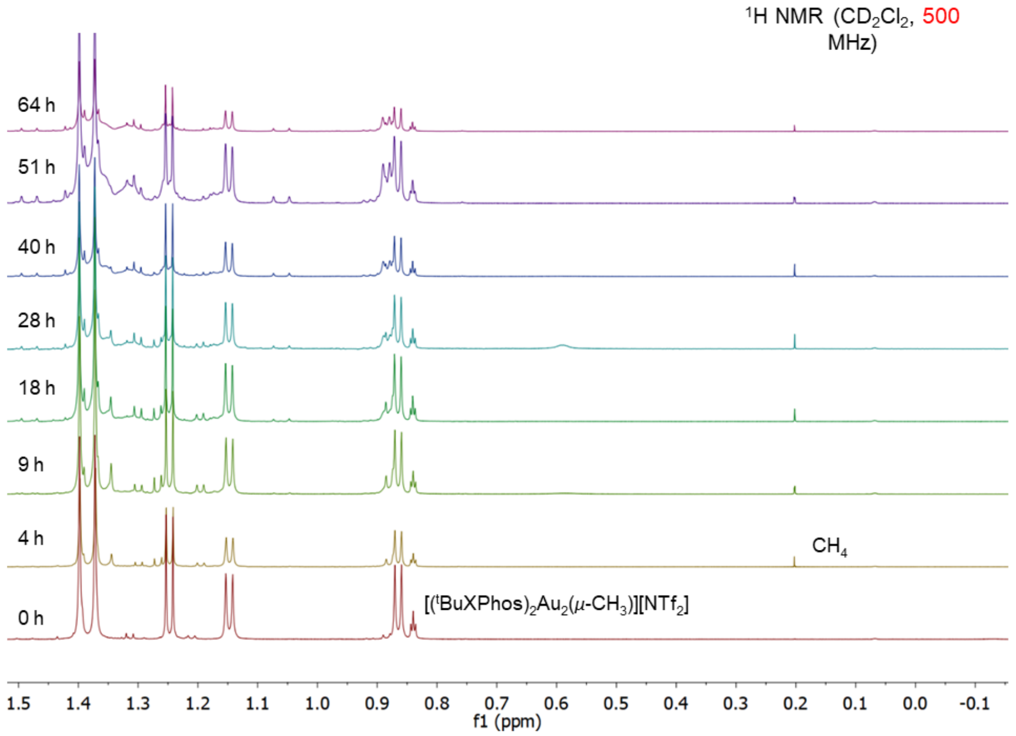

Figure S14. $^{31}P\{^1H\}$ NMR monitoring of the thermolysis of compound **4f** at 90 ºC in $C_2D_4Cl_2$.

V. DFT calculations.

Calculations were performed with Gaussian 09, Revision E.01 software package[5] employing the range-separated hybrid functional ωB97X-D[6] without geometry constraints. The 6-31G(d,p)[7] basis set was used to represent the C, H, P, N, O, S, F and B atoms and the Stuttgart/Dresden Effective Core Potential and its associated basis set (SDD)[8] to describe the Au atoms. Solvent effects (dichloromethane) were included during optimization with the SMD continuum model.[9] Vibrational analysis was carried out on the stationary points to characterize them as minima or transition states as well as to calculate the thermal corrections to enthalpy and free energy. Free energies were corrected (ΔG_{qh}) to account for errors associated with the harmonic oscillator approximation. Thus, according to Truhlar's quasi harmonic approximation, all vibrational frequencies below 100 cm^{-1} were set to this value so that the entropy contribution was not overestimated.[10] These anharmonic corrections were calculated with the Goodvibes code.[11]

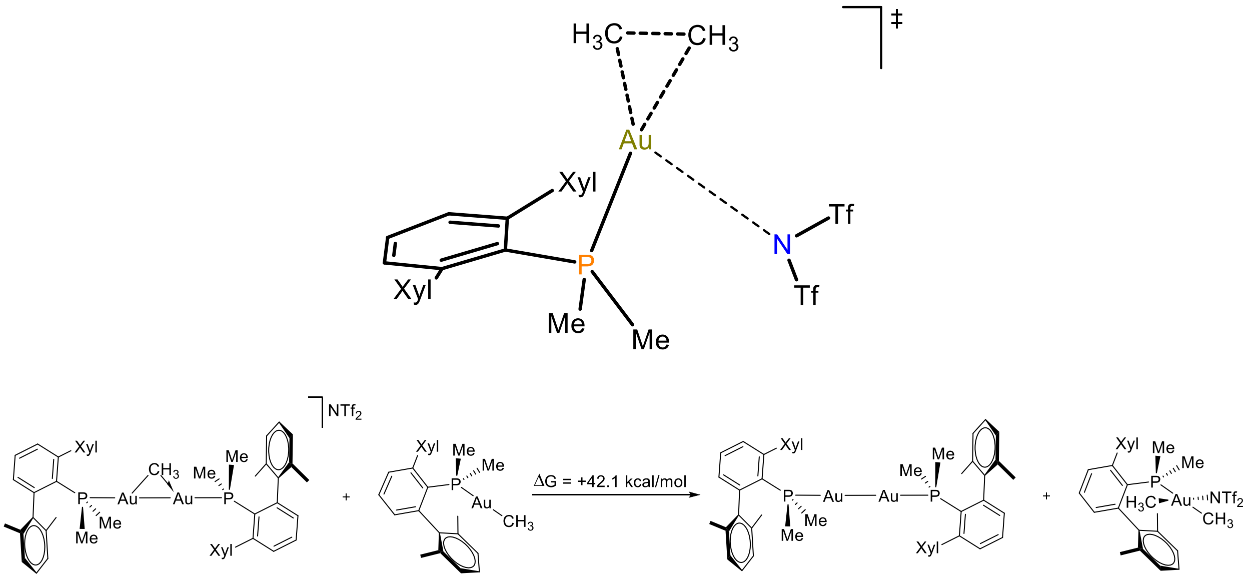

Figure S15. Top: transition state for ethane liberation at mononuclear Au(III) species, at +16.1 kcal/mol relative to $Au(CH_3)_2(PMe_2Ar^{Xyl2})(NTf_2)$. Bottom: formation of $[Au_2(PMe_2Ar^{Xyl2})_2]$ and $Au(CH_3)_2(PMe_2Ar^{Xyl2})(NTf_2)$ from complexes **4a** and **1a**; ΔG = +42.1 kcal/mol.

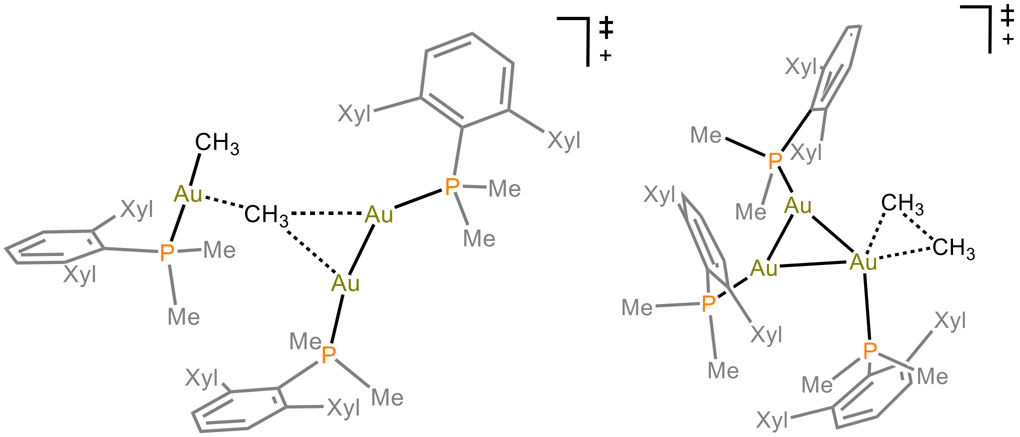

Figure S16. High energy CH_3^+ transfer (left, **TS1**; $\Delta G^‡$ = 47.0 kcal/mol) and C-C coupling (right, **TS2;** $\Delta G^‡$ = 33.6 kcal/mol) transition states.

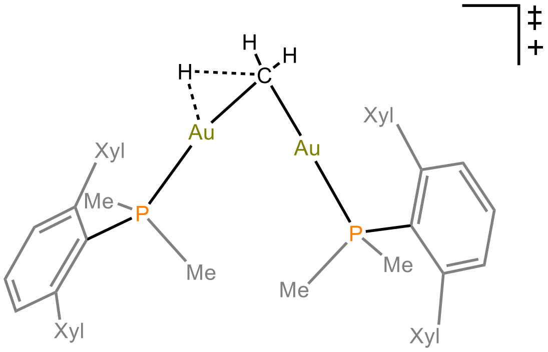

Figure S17. Transition state for the formation of Au–H and Au–CH_2 moieties, +36.2 kcal/mol relative to **4a**.

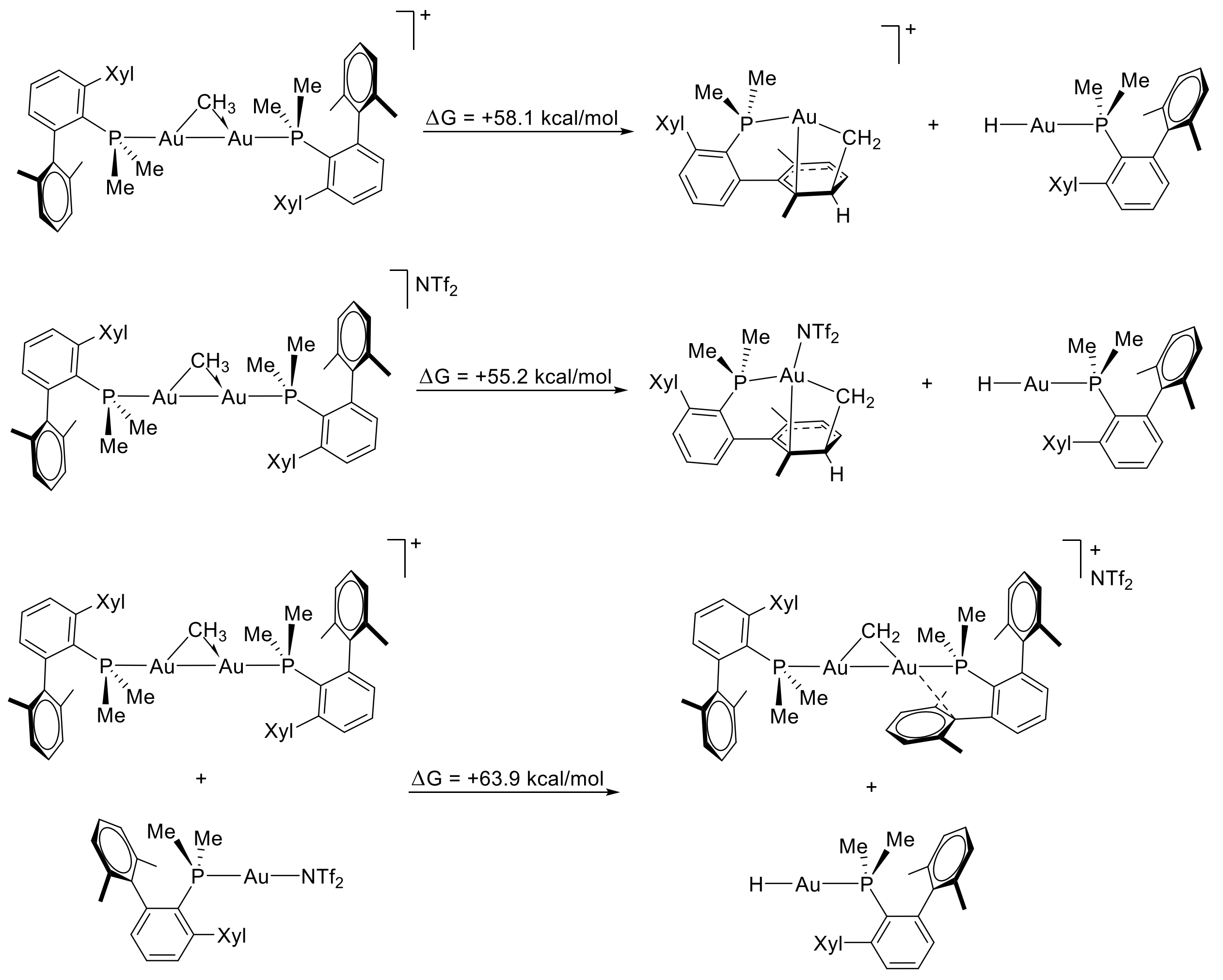

Figure S18. Top: Formation of a neutral gold hydride and a cationic $AuCH_2$ complex from **4a**; ΔG = +58.1 kcal/mol. Mid: Formation of a neutral gold hydride and a $AuCH_2$ complex including bound triflimide **4a**; ΔG = +55.2 kcal/mol. Bottom: Formation of a bridging dicationic Au_2CH_2 complex from **4a** and $Au(PMe_2Ar^{Xyl2})(NTf_2)$; ; ΔG = +63.9 kcal/mol.

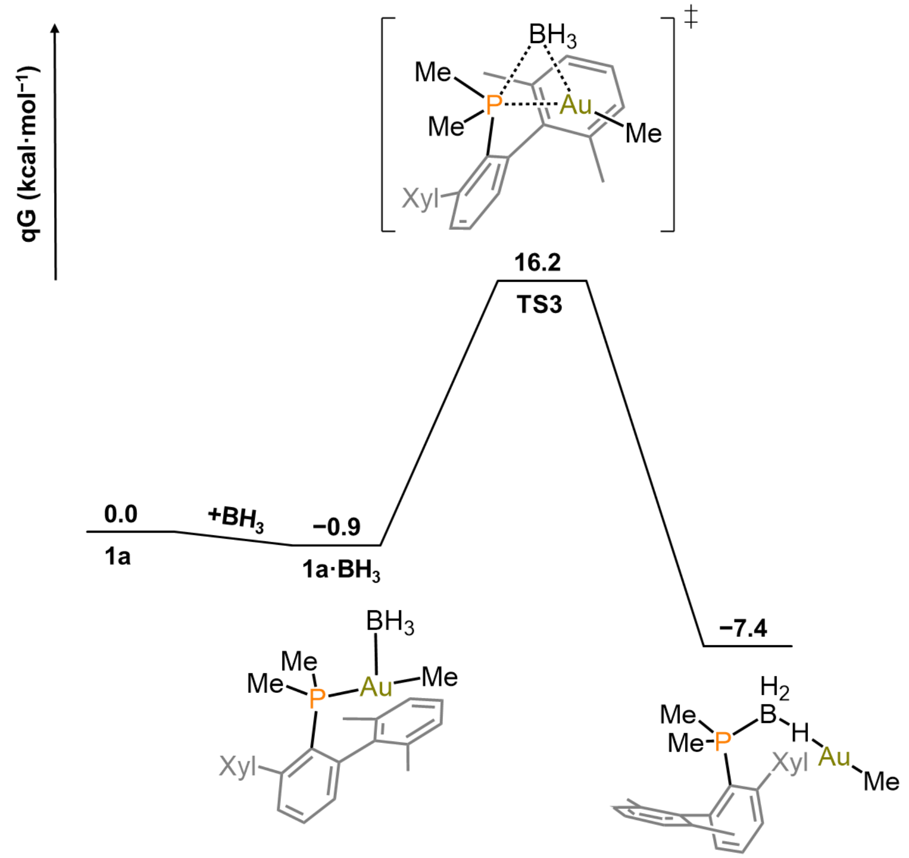

Figure S19. DFT-computed reaction coordinated for the BH_3-mediated P-Au bond cleavage from $Au(CH_3)(PMe_2Ar^{Xyl2})$ (**1a**).

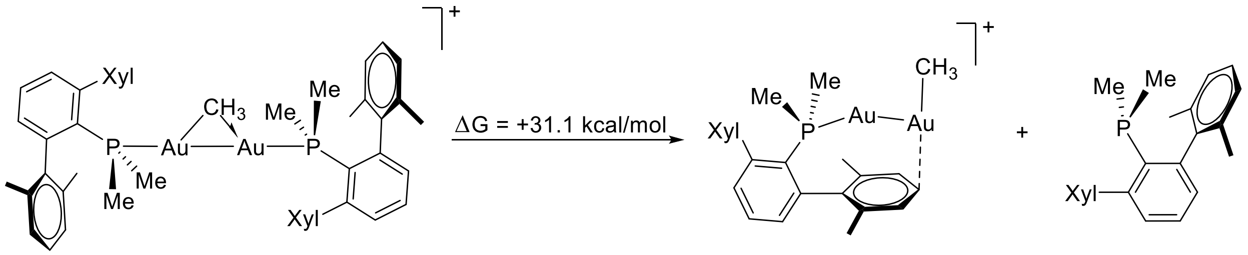

Figure S20. Phosphine dissociation from $[Au_2(\mu\text{-}CH_3)(PMe_2Ar^{Xyl2})_2][NTf_2]$ (**4a**) is calculated to occur with ΔG = +31.1 kcal/mol.

VI. GC-MS spectra.

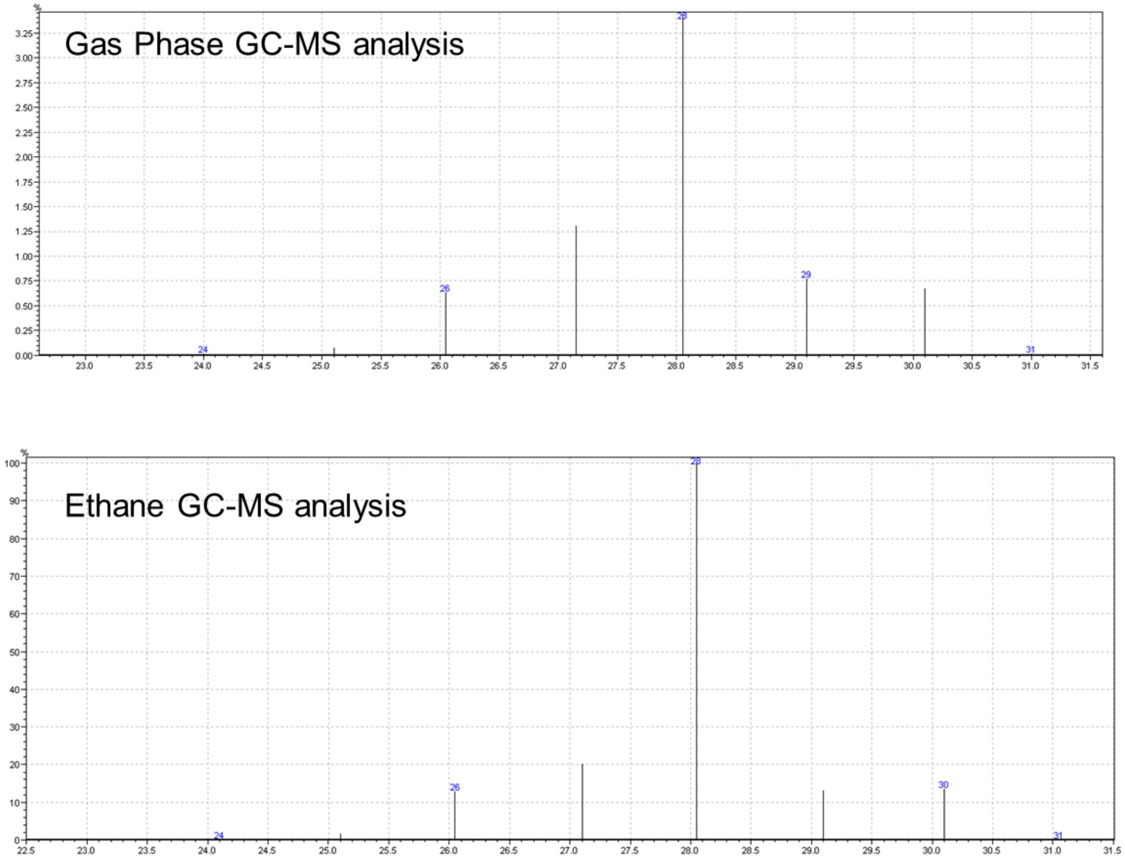

Figure S21. MS spectra from GC-MS analysis of the headspace from the C-C coupling reaction from [Au_2(μ-CH_3)($XPhos)_2$][NTf_2] (**4f**) (above) and pure ethane (below).

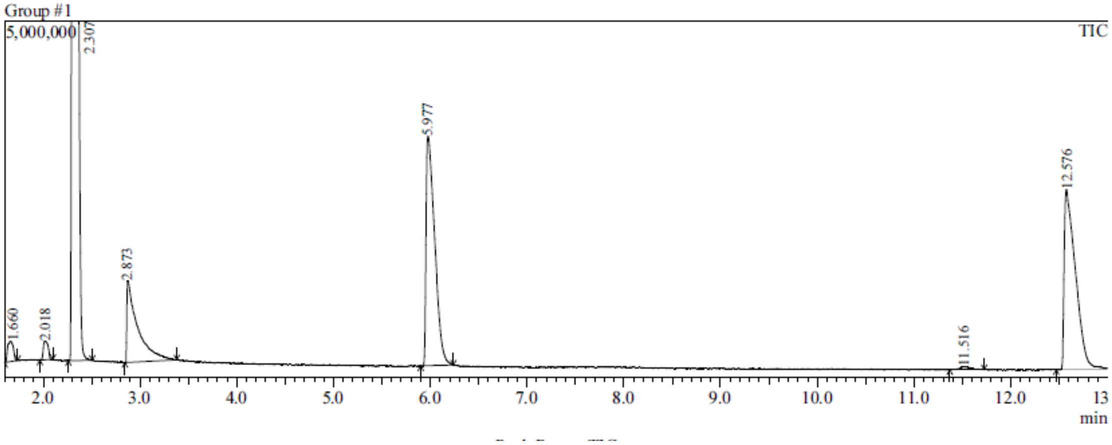

Peak Report TIC

Peak#	R.Time	Area	Area%	Mark	Name
1	1.660	1134110	0.96	MI	Carbon dioxide (CAS) Dry ice
2	2.018	904971	0.76	MI	Ethene (CAS) Ethylene
3	2.307	66970637	56.50	MI	Ethane (CAS) Bimethyl
4	2.873	8837406	7.46	MI	Water (CAS) Ice
5	5.977	19856765	16.75	MI	Propane (CAS) R 290
6	11.516	348395	0.29	MI	Propane, 2-methyl- (CAS) Isobutane
7	12.576	20474715	17.27	MI	Butane (CAS) n-Butane
		118526999	100.00		

Figure S22. GC-MS spectra and analysis of the reaction described in Scheme 6b and Figure 8 between [$Au_2(\mu\text{-}CH_3)(PMe_2Ar^{Xyl2})_2$] (**4a**) (0.012 mmol) and [$Au(C_2H_5)(PMe_2Ar^{Xyl2})$] (**2a**) (0.012 mmol) after 3 hours at 25 ºC.

VII. NMR spectra of gold (I) compounds.

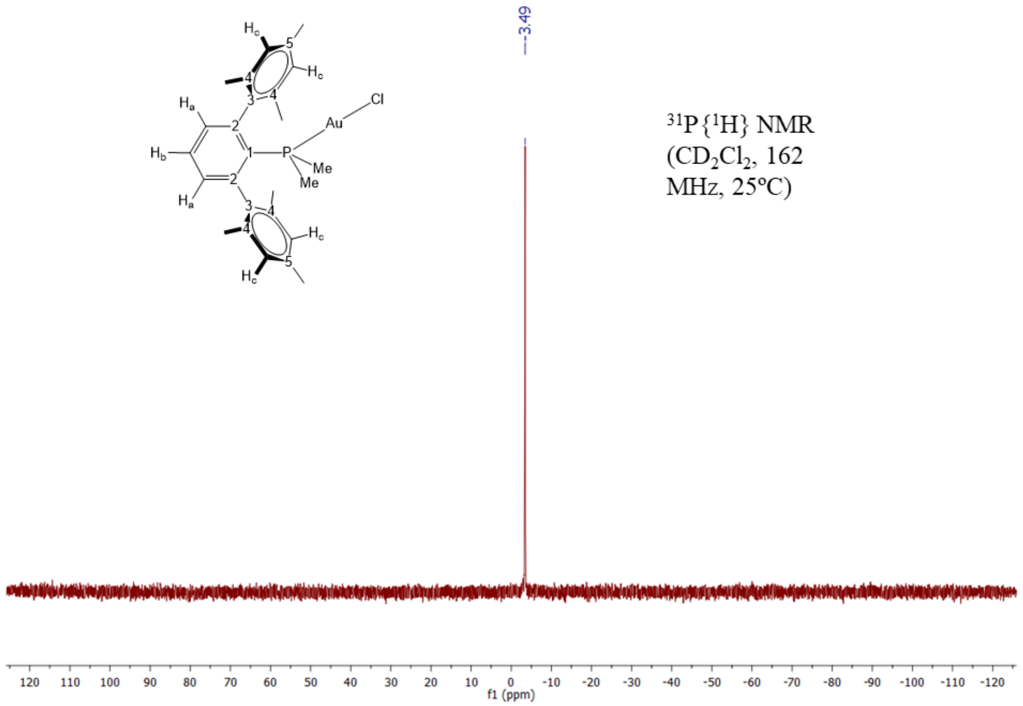

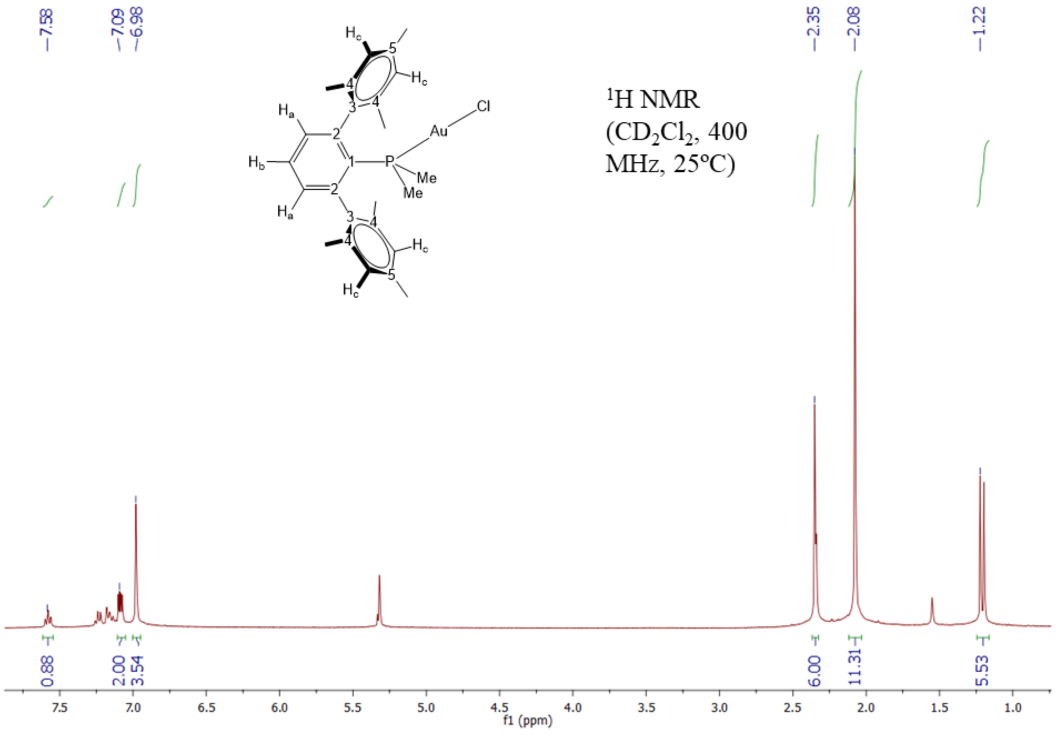

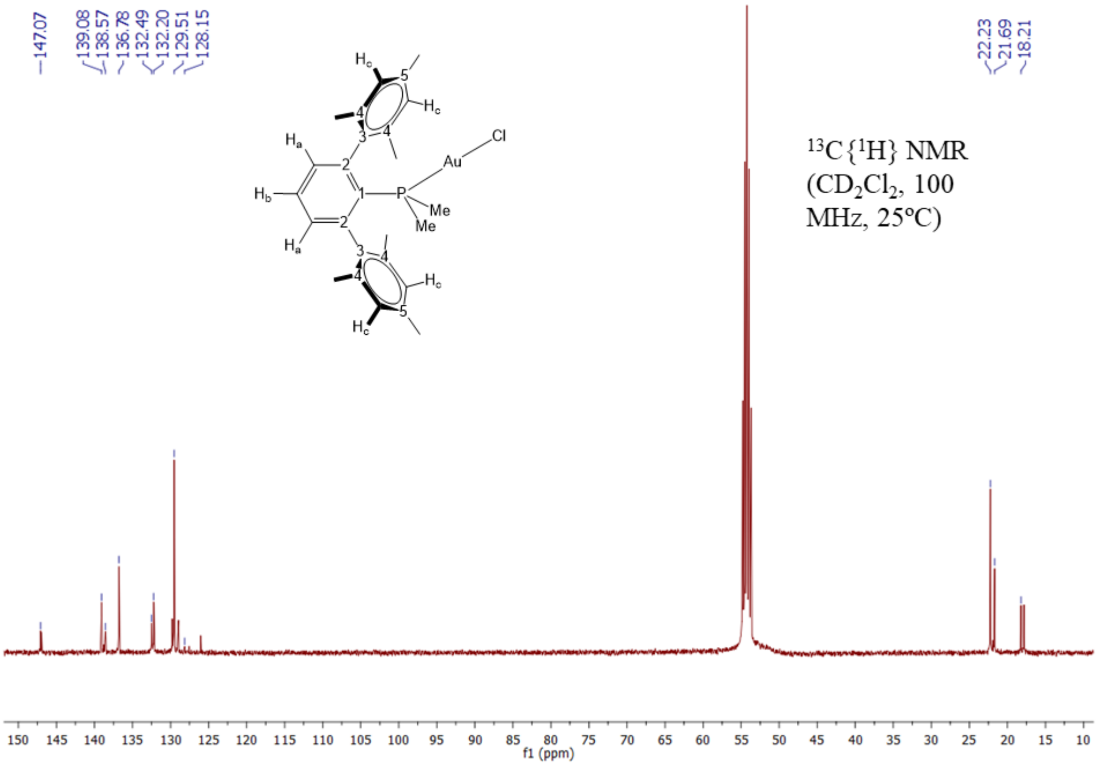
147.07
139.08
138.57
136.78
132.49
132.20
129.51
128.15
22.23
21.69
18.21
$^{13}C\{^1H\}$ NMR
(CD_2Cl_2, 100
MHz, 25ºC)
f1 (ppm)

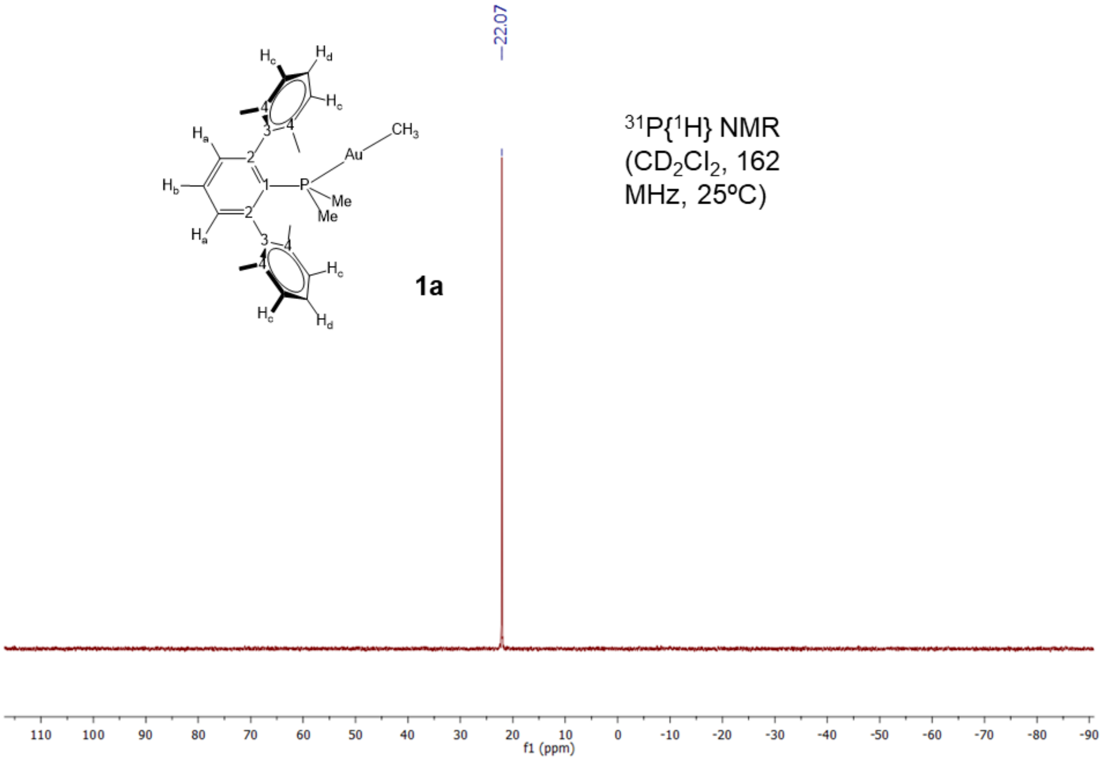
22.07
$^{31}P\{^1H\}$ NMR
(CD_2Cl_2, 162
MHz, 25ºC)
1a
f1 (ppm)

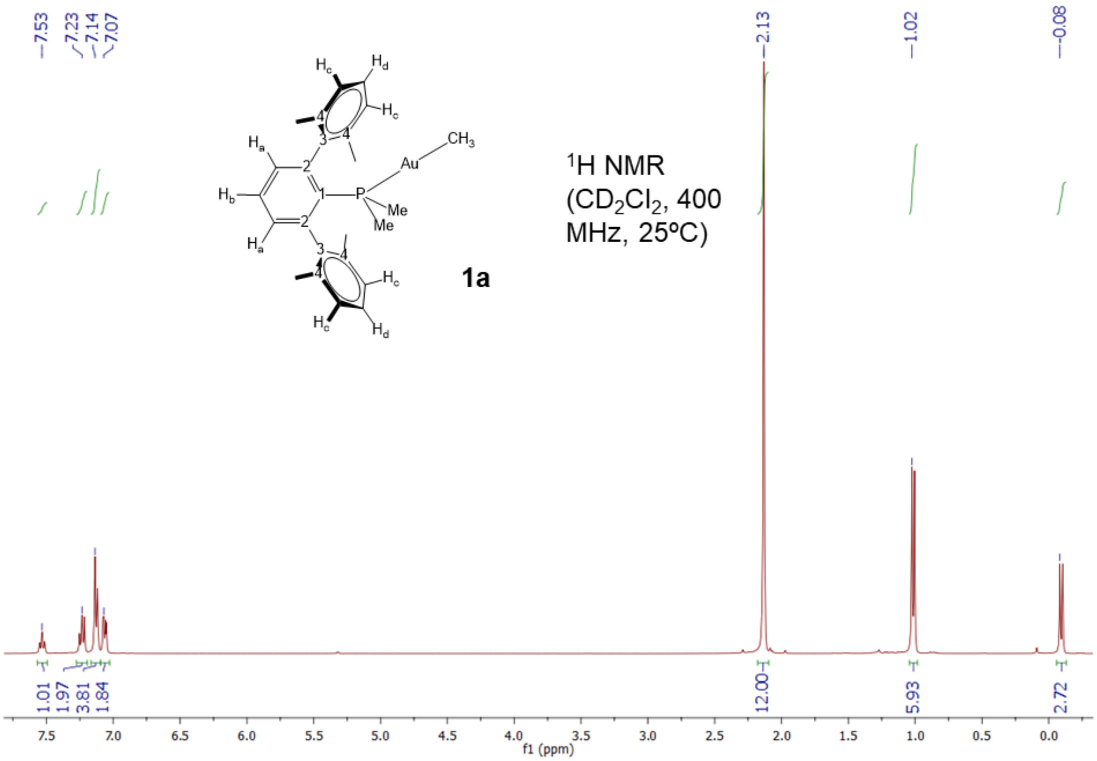
7.53
7.23
7.14
7.07
2.13
1.02
0.08
1a
^{1}H NMR
(CD_2Cl_2, 400
MHz, 25ºC)
1.01
1.97
3.81
1.84
12.00
5.93
2.72
f1 (ppm)

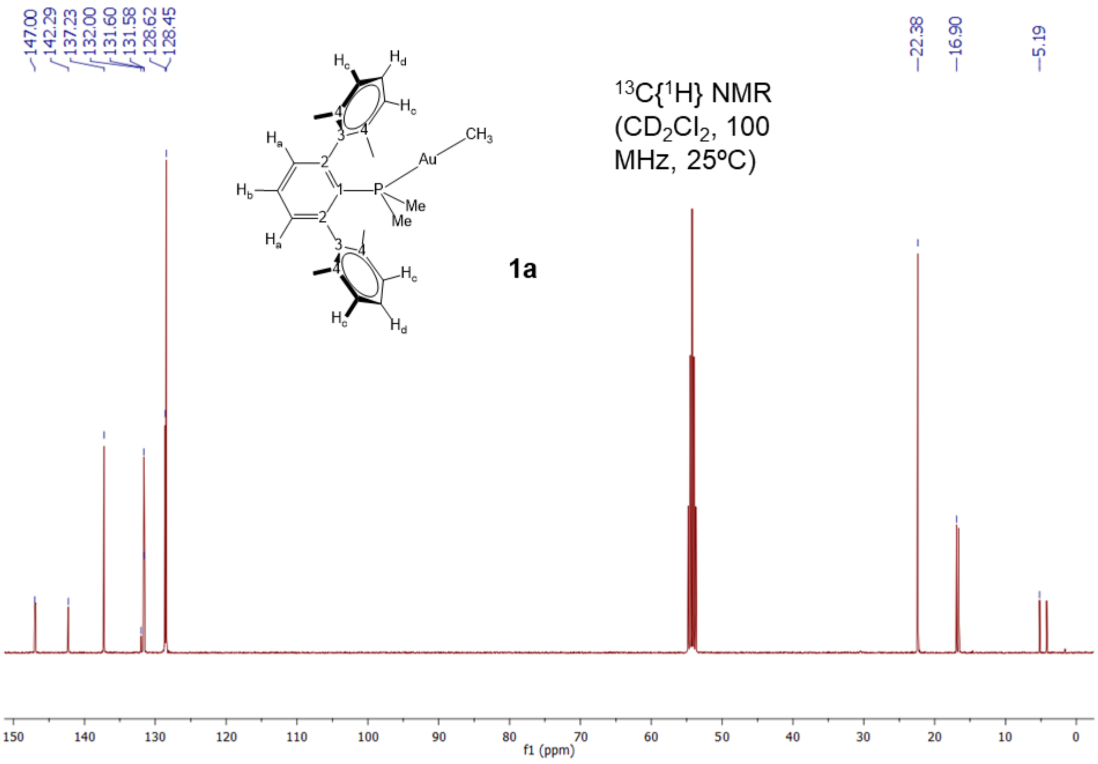
147.00
142.29
137.23
132.00
131.60
131.58
128.62
128.45
22.38
16.90
5.19
1a
^{13}C{^{1}H} NMR
(CD_2Cl_2, 100
MHz, 25ºC)
f1 (ppm)

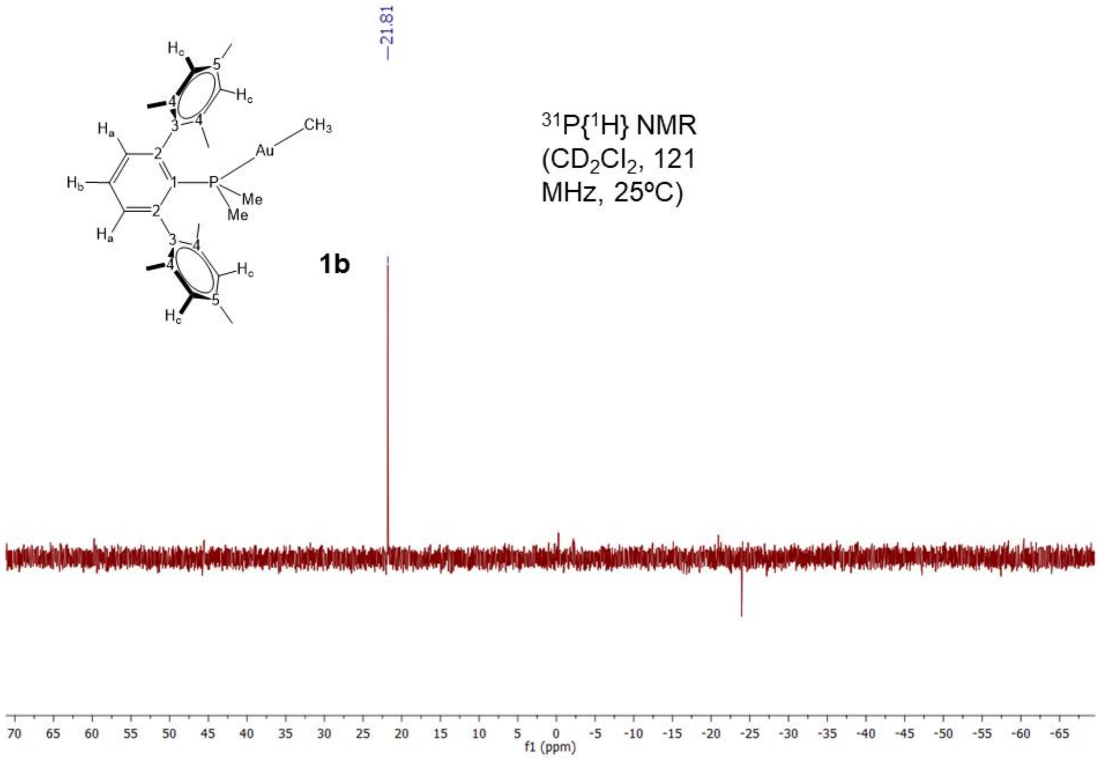
-21.81
$^{31}P\{^1H\}$ NMR
(CD_2Cl_2, 121
MHz, 25ºC)
1b
f1 (ppm)

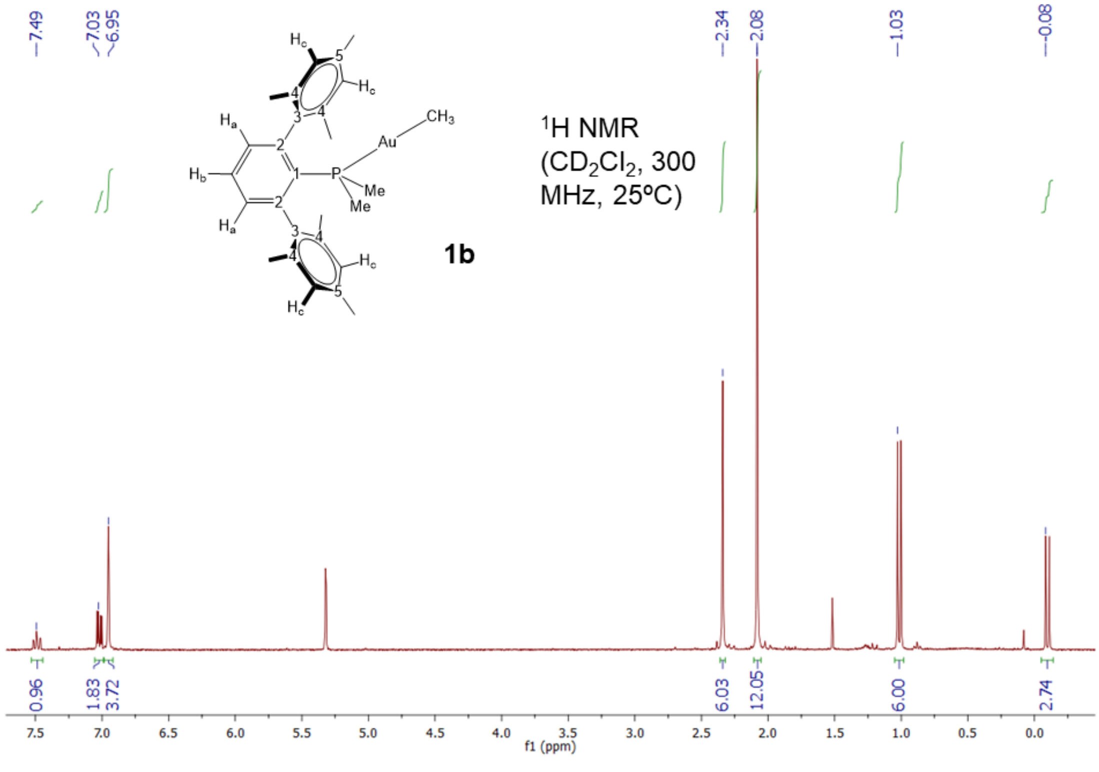
-7.49
-7.03
-6.95
-2.34
-2.08
-1.03
-0.08
^1H NMR
(CD_2Cl_2, 300
MHz, 25ºC)
1b
0.96
1.83
3.72
6.03
12.05
6.00
2.74
f1 (ppm)

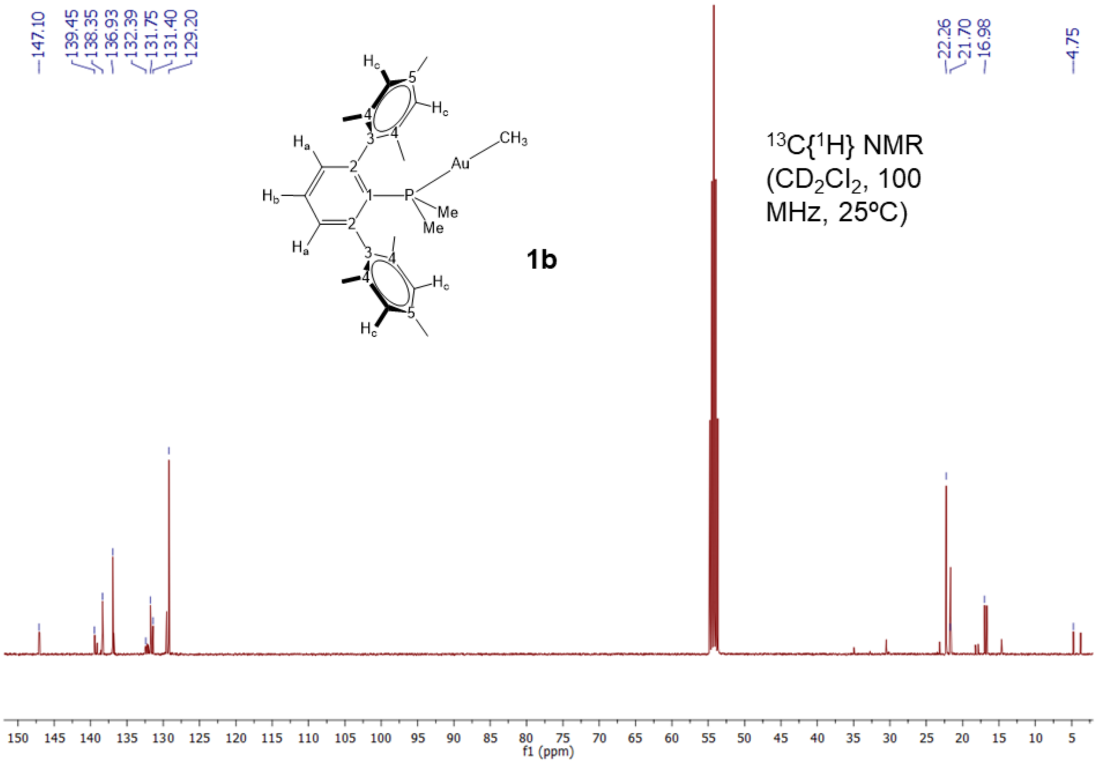
147.10
139.45
138.35
136.93
132.39
131.75
131.40
129.20
22.26
21.70
16.98
4.75
1b
$^{13}C\{^1H\}$ NMR
(CD_2Cl_2, 100
MHz, 25ºC)
f1 (ppm)

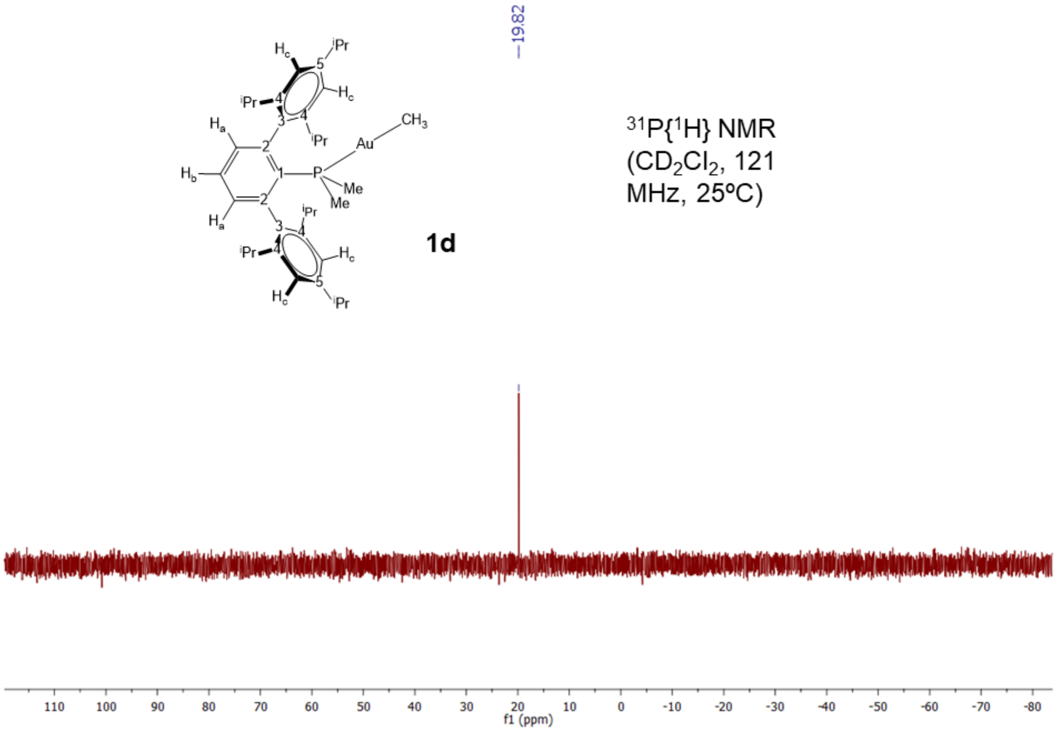
19.82
1d
$^{31}P\{^1H\}$ NMR
(CD_2Cl_2, 121
MHz, 25ºC)
f1 (ppm)

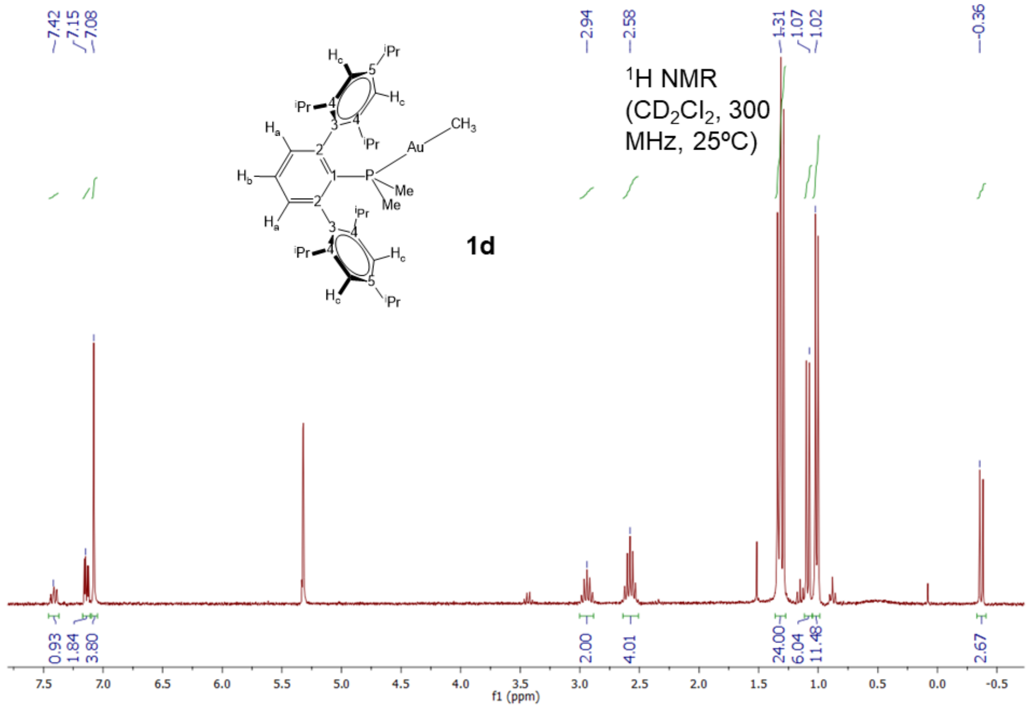
7.42
7.15
7.08
2.94
2.58
1.31
1.07
1.02
-0.36
1H NMR
(CD2Cl2, 300
MHz, 25ºC)
1d
0.93
1.84
3.80
2.00
4.01
24.00
6.04
11.48
2.67
f1 (ppm)

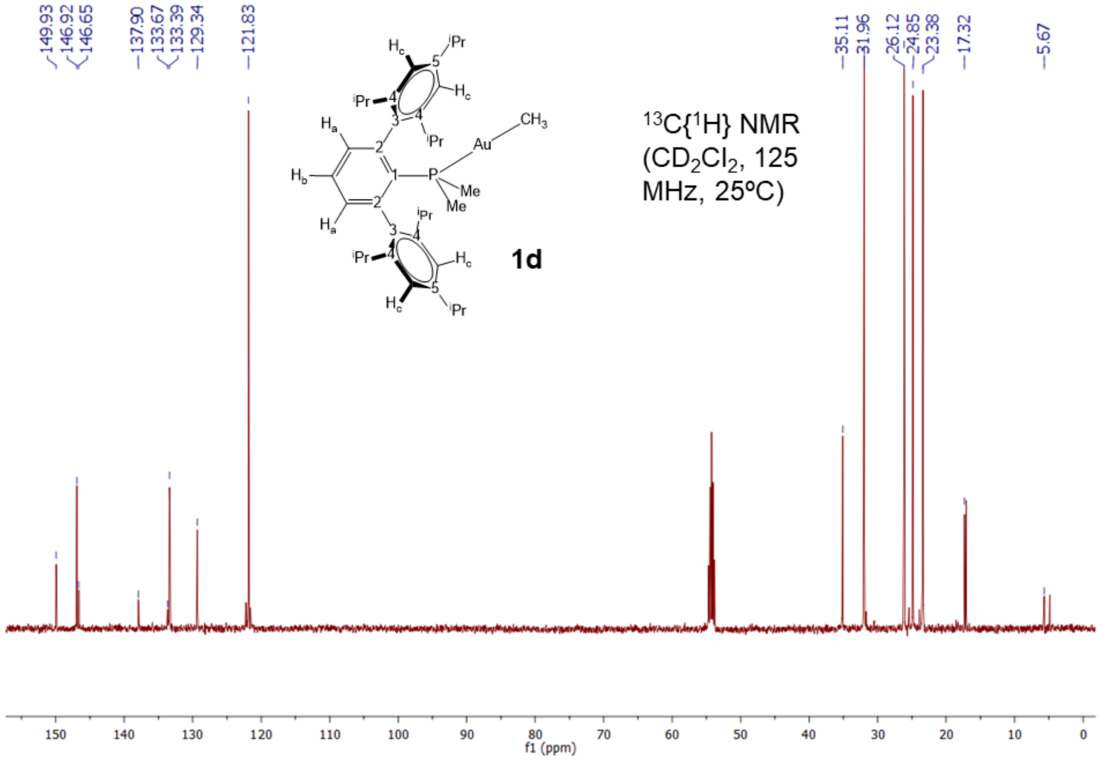
149.93
146.92
146.65
137.90
133.67
133.39
129.34
121.83
35.11
31.96
26.12
24.85
23.38
17.32
5.67
13C{1H} NMR
(CD2Cl2, 125
MHz, 25ºC)
1d
f1 (ppm)

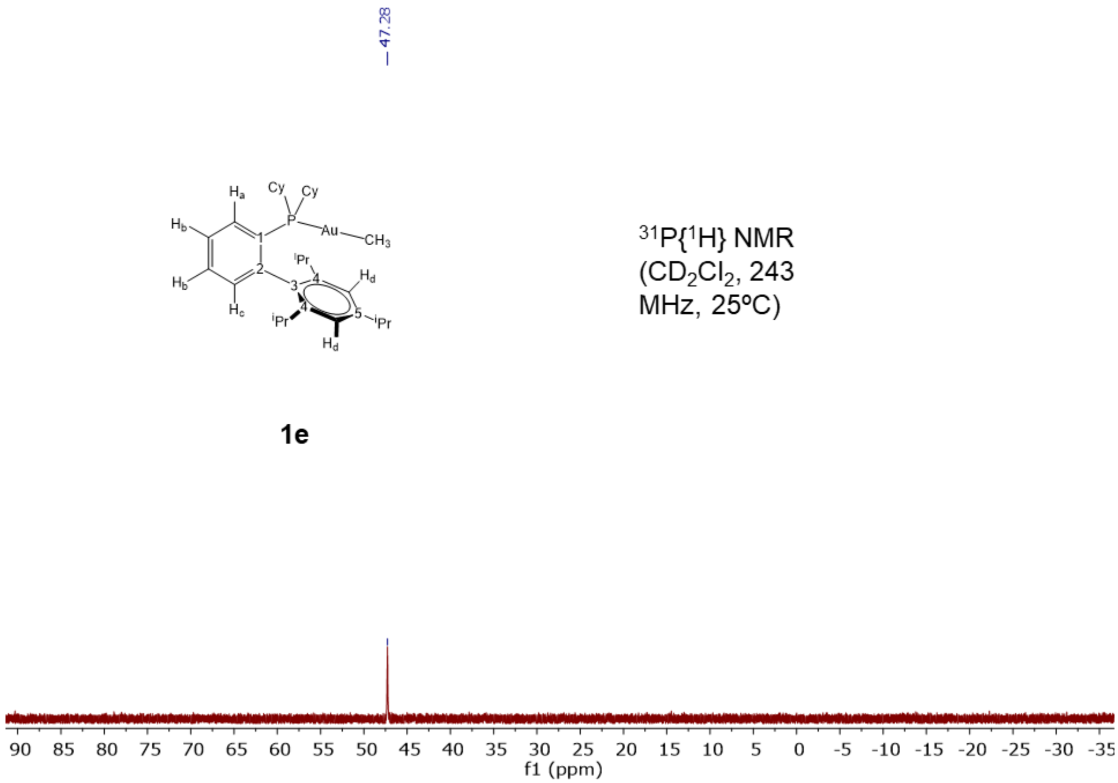
47.28
1e
$^{31}P\{^{1}H\}$ NMR
(CD_2Cl_2, 243
MHz, 25ºC)
90 85 80 75 70 65 60 55 50 45 40 35 30 25 20 15 10 5 0 -5 -10 -15 -20 -25 -30 -35
f1 (ppm)

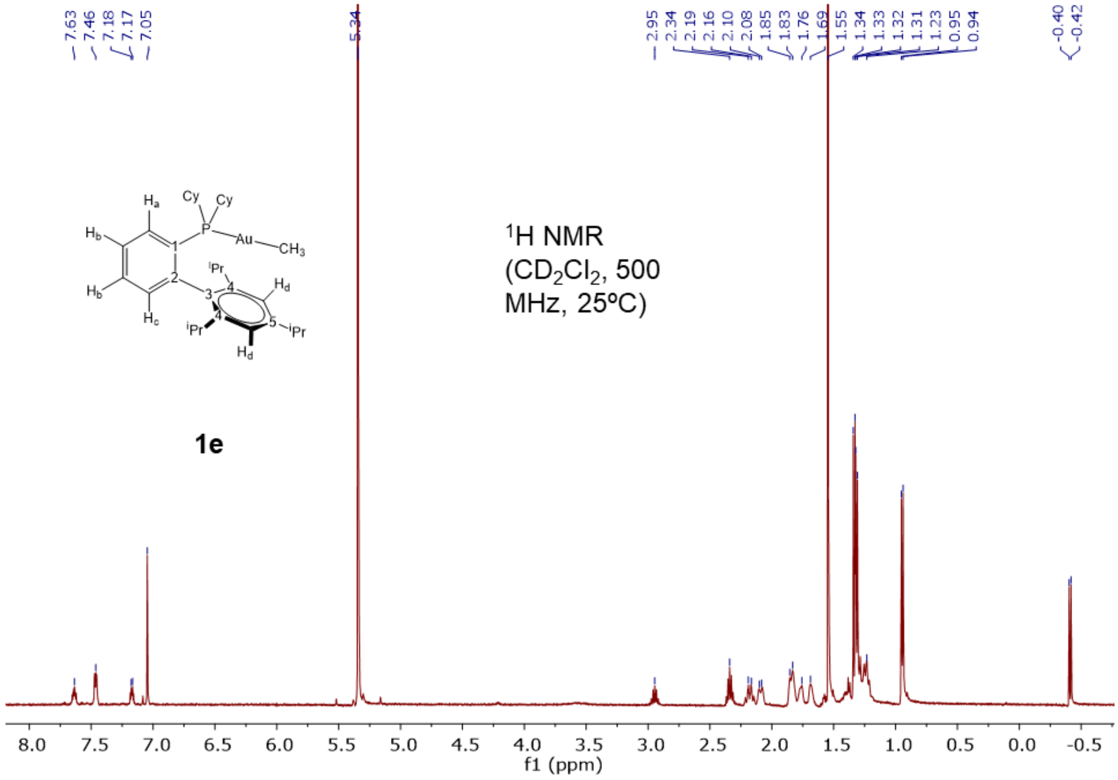
7.63
7.46
7.18
7.17
7.05
5.34
2.95
2.34
2.19
2.16
2.10
2.08
1.85
1.83
1.76
1.69
1.55
1.34
1.33
1.32
1.31
1.23
0.95
0.94
-0.40
-0.42
1e
^{1}H NMR
(CD_2Cl_2, 500
MHz, 25ºC)
8.0 7.5 7.0 6.5 6.0 5.5 5.0 4.5 4.0 3.5 3.0 2.5 2.0 1.5 1.0 0.5 0.0 -0.5
f1 (ppm)

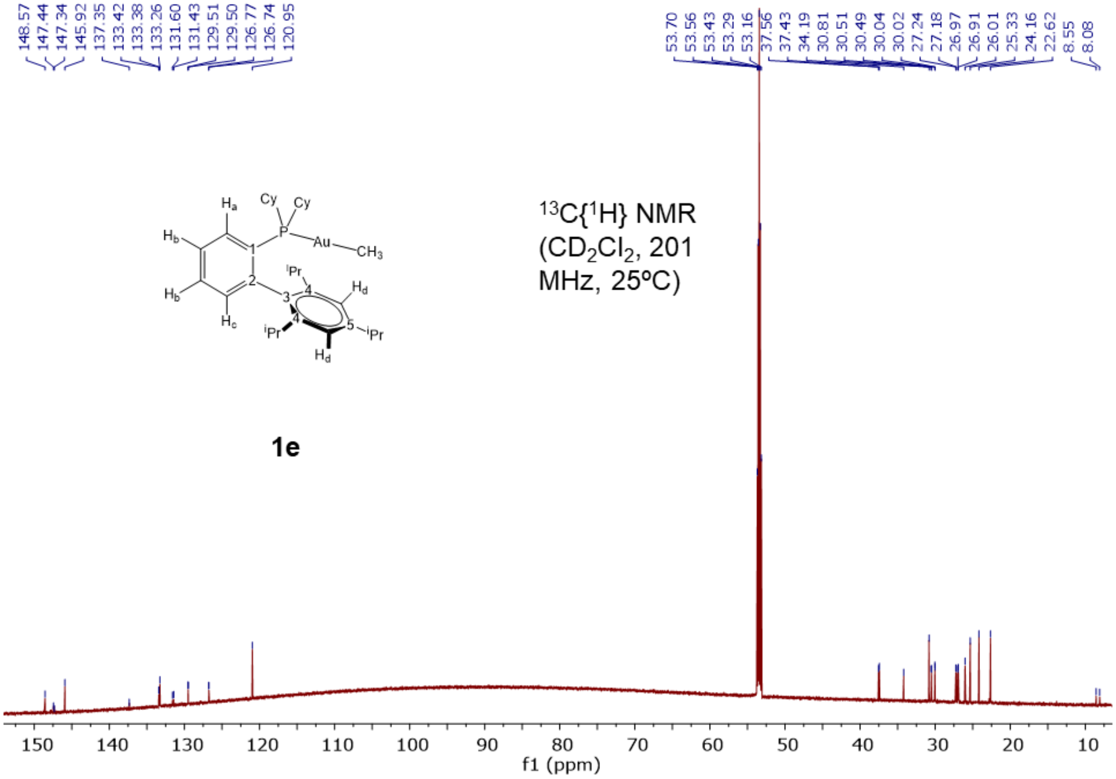
148.57
147.44
147.34
145.92
137.35
133.42
133.38
133.26
131.60
131.43
129.51
129.50
126.77
126.74
120.95
53.70
53.56
53.43
53.29
53.16
37.56
37.43
34.19
30.81
30.51
30.49
30.04
30.02
27.24
27.18
26.97
26.91
26.01
25.33
24.16
22.62
8.55
8.08
Cy
Cy
P
Au
CH_3
H_a
H_b
H_b
H_c
H_d
H_d
^iPr
^iPr
^iPr
1
2
3
4
4
5
$^{13}C\{^1H\}$ NMR
(CD_2Cl_2, 201
MHz, 25ºC)
1e
150 140 130 120 110 100 90 80 70 60 50 40 30 20 10
f1 (ppm)

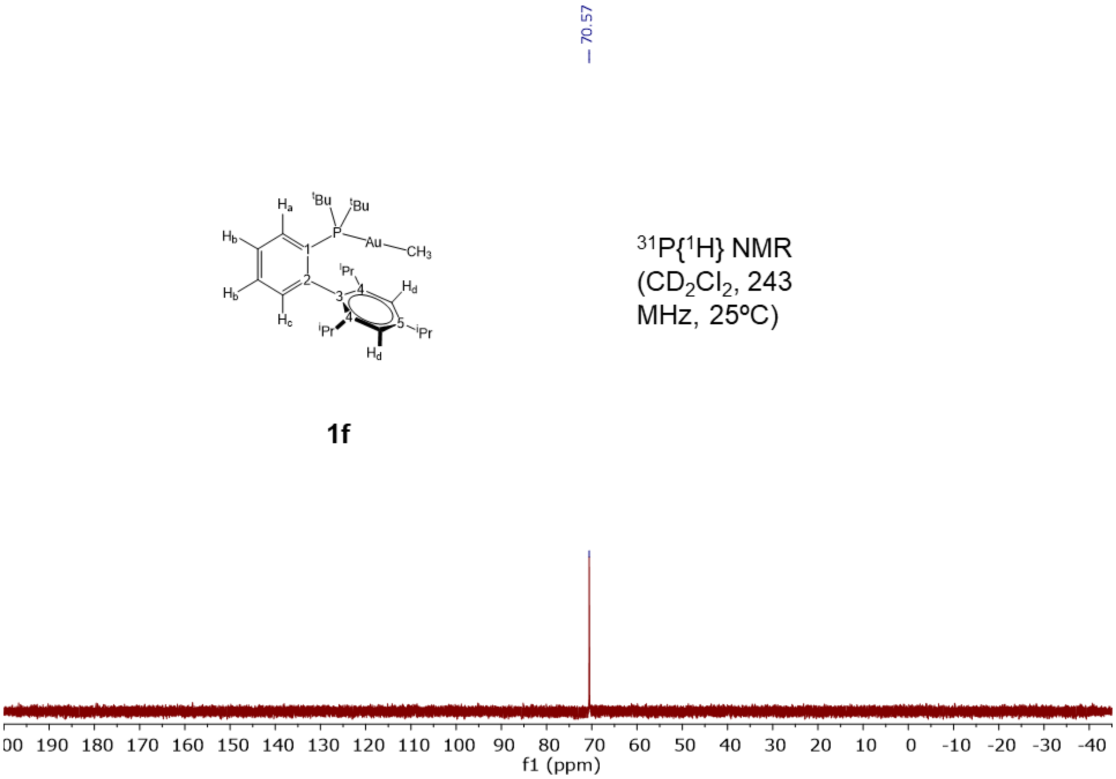
70.57
^tBu
^tBu
P
Au
CH_3
H_a
H_b
H_b
H_c
H_d
H_d
^iPr
^iPr
^iPr
1
2
3
4
4
5
$^{31}P\{^1H\}$ NMR
(CD_2Cl_2, 243
MHz, 25ºC)
1f
00 190 180 170 160 150 140 130 120 110 100 90 80 70 60 50 40 30 20 10 0 -10 -20 -30 -40
f1 (ppm)

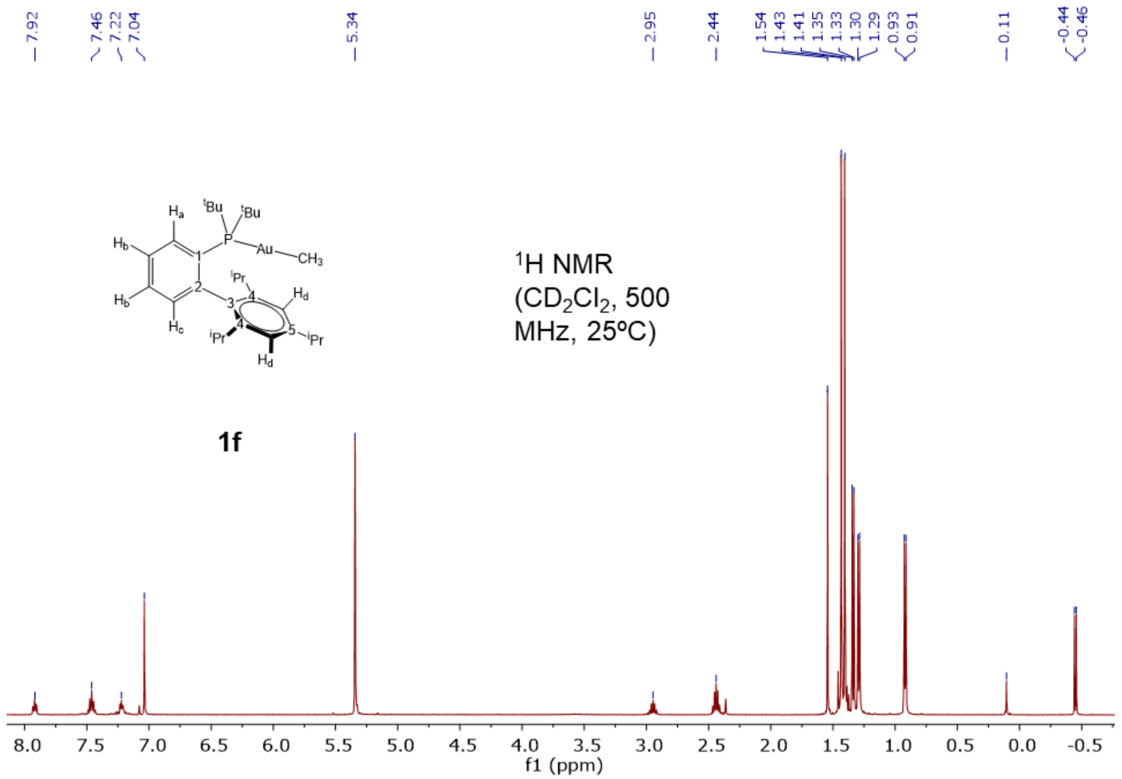

7.92
7.46
7.22
7.04
5.34
2.95
2.44
1.54
1.43
1.41
1.35
1.33
1.30
1.29
0.93
0.91
0.11
-0.44
-0.46
1f
^{1}H NMR (CD$_2$Cl$_2$, 500 MHz, 25ºC)
f1 (ppm)

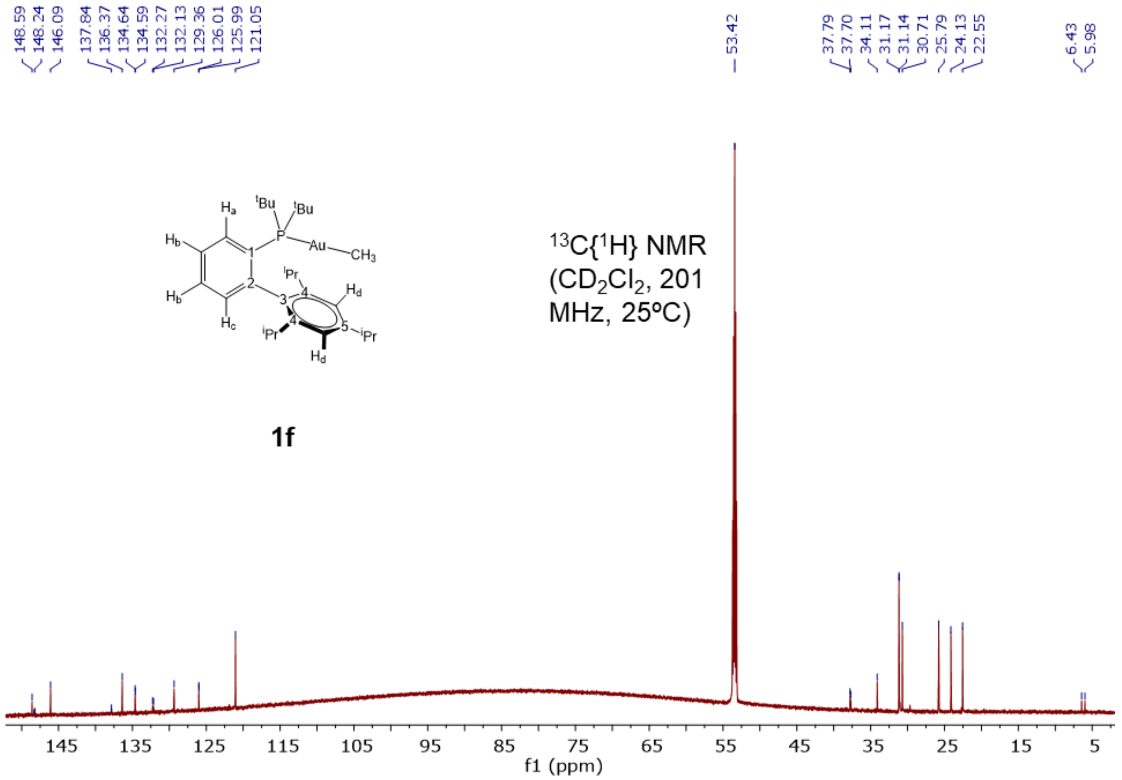

148.59
148.24
146.09
137.84
136.37
134.64
134.59
132.27
132.13
129.36
126.01
125.99
121.05
53.42
37.79
37.70
34.11
31.17
31.14
30.71
25.79
24.13
22.55
6.43
5.98
1f
^{13}C{^{1}H} NMR (CD$_2$Cl$_2$, 201 MHz, 25ºC)
f1 (ppm)

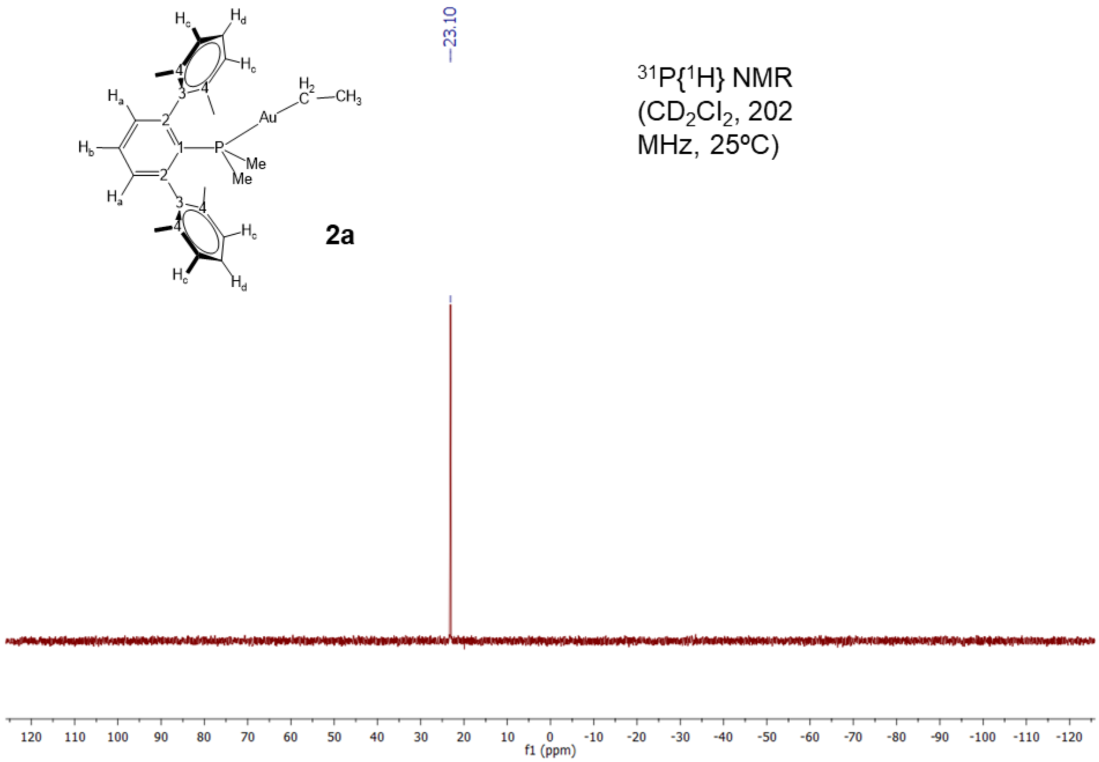

23.10
$^{31}P\{^1H\}$ NMR
(CD_2Cl_2, 202
MHz, 25ºC)
2a
f1 (ppm)

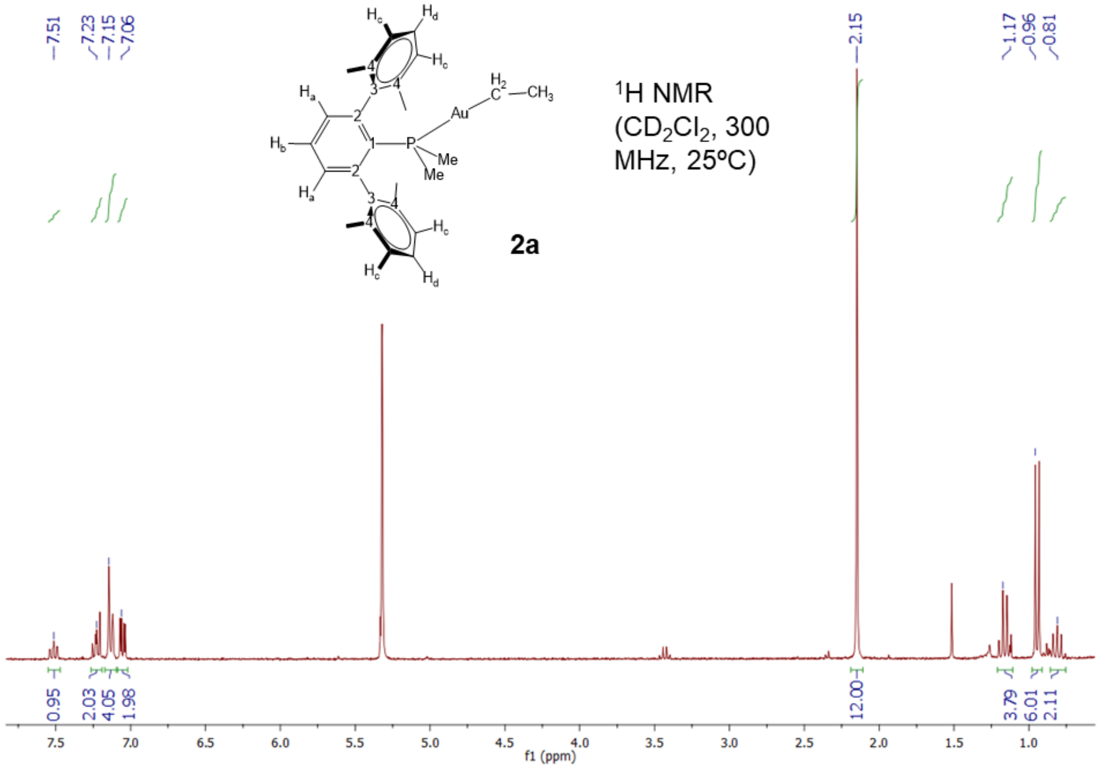

7.51
7.23
7.15
7.06
2.15
1.17
0.96
0.81
^1H NMR
(CD_2Cl_2, 300
MHz, 25ºC)
2a
0.95
2.03
4.05
1.98
12.00
3.79
6.01
2.11
f1 (ppm)

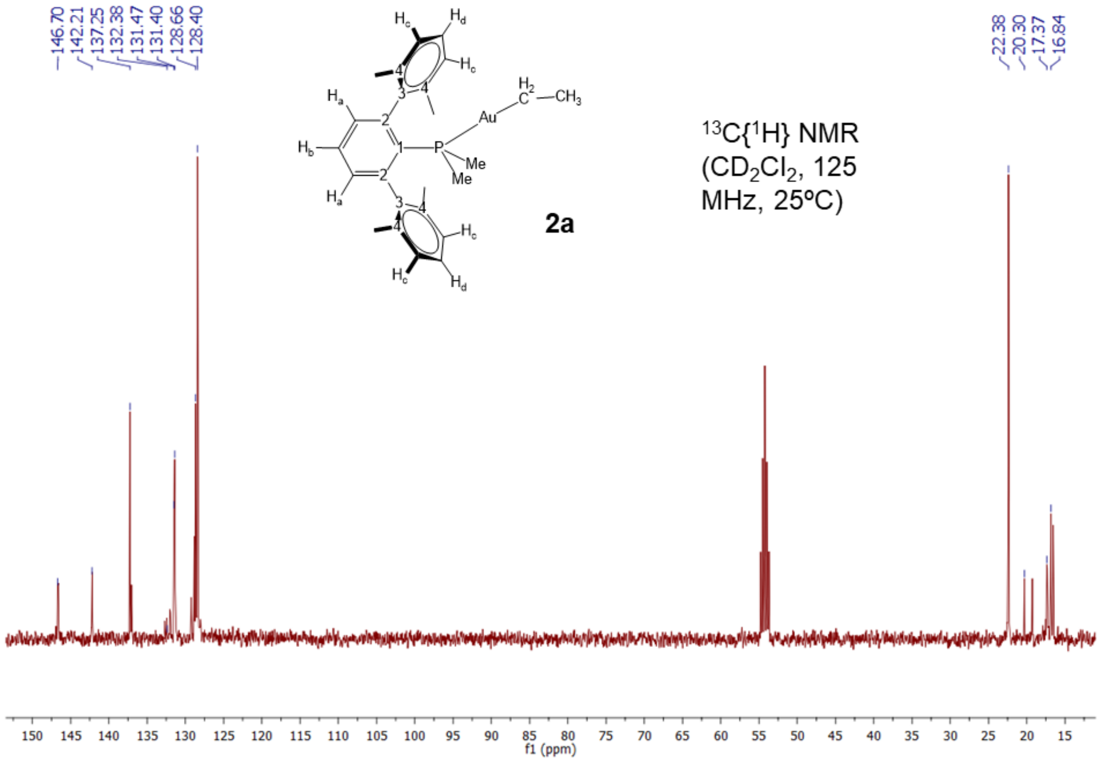
146.70
142.21
137.25
132.38
131.47
131.40
128.66
128.40
22.38
20.30
17.37
16.84
2a
$^{13}C\{^1H\}$ NMR
(CD_2Cl_2, 125
MHz, 25ºC)
f1 (ppm)

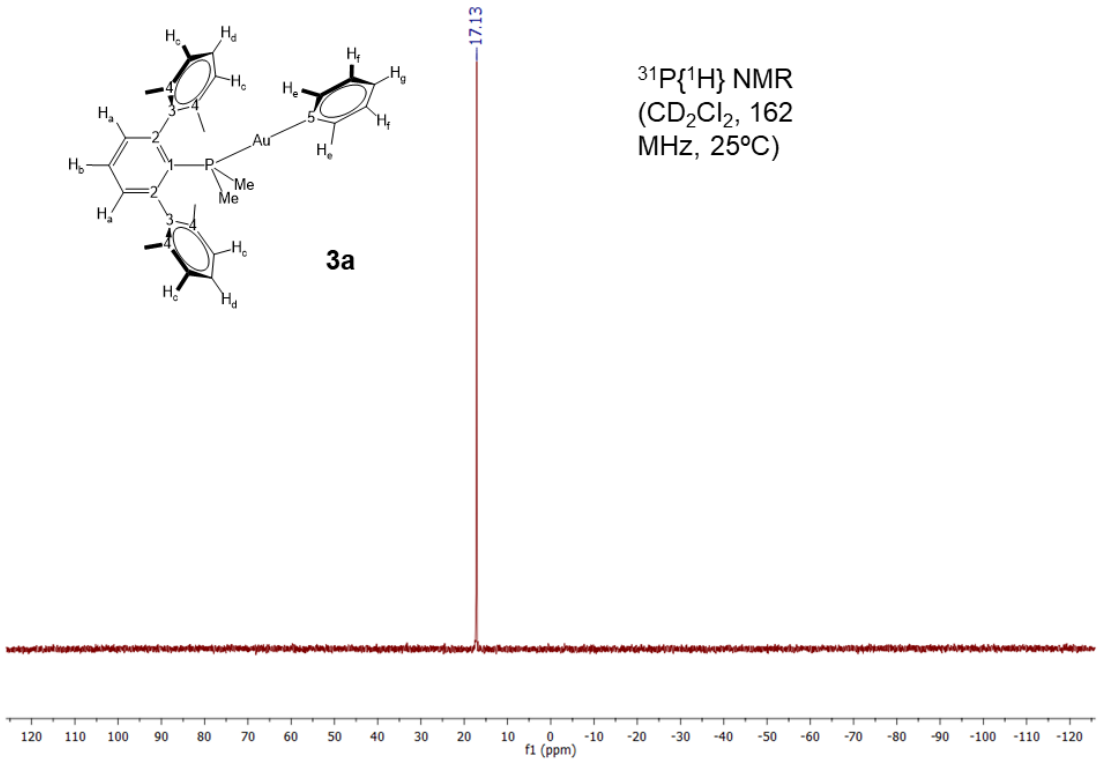
17.13
3a
$^{31}P\{^1H\}$ NMR
(CD_2Cl_2, 162
MHz, 25ºC)
f1 (ppm)

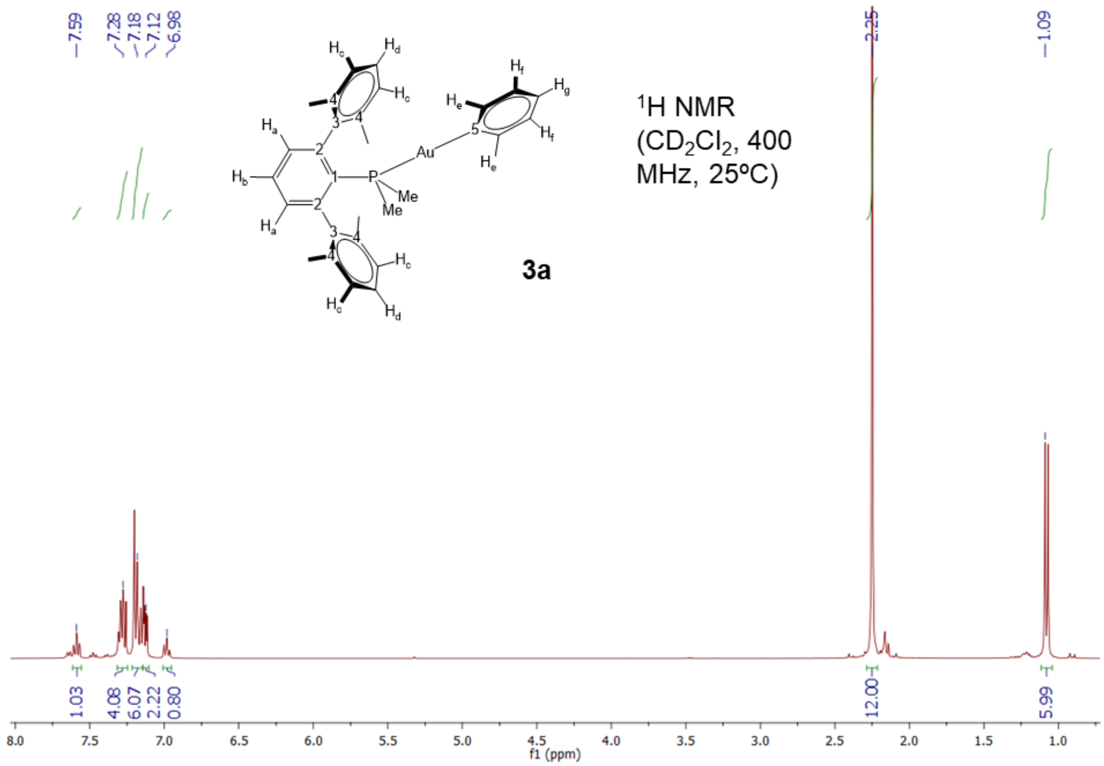

7.59
7.28
7.18
7.12
6.98
2.25
1.09
^{1}H NMR
(CD$_2$Cl$_2$, 400
MHz, 25ºC)
3a
1.03
4.08
6.07
2.22
0.80
12.00
5.99
f1 (ppm)

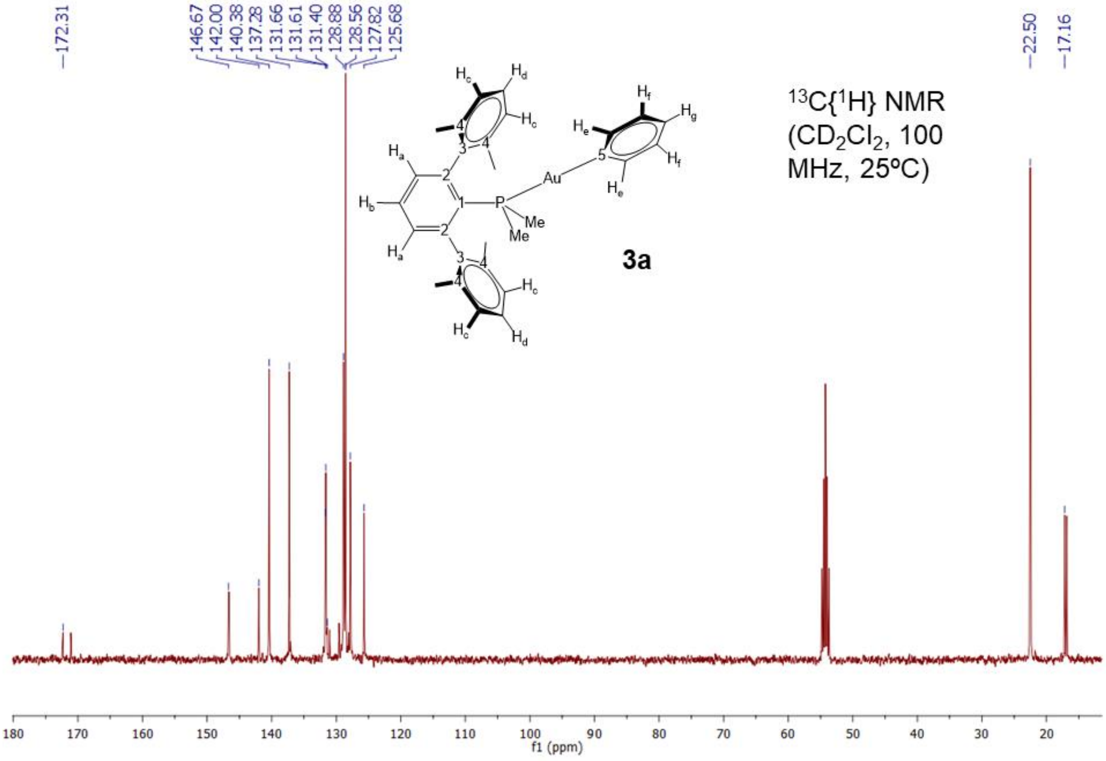

172.31
146.67
142.00
140.38
137.28
131.66
131.61
131.40
128.88
128.56
127.82
125.68
22.50
17.16
^{13}C{^{1}H} NMR
(CD$_2$Cl$_2$, 100
MHz, 25ºC)
3a
f1 (ppm)

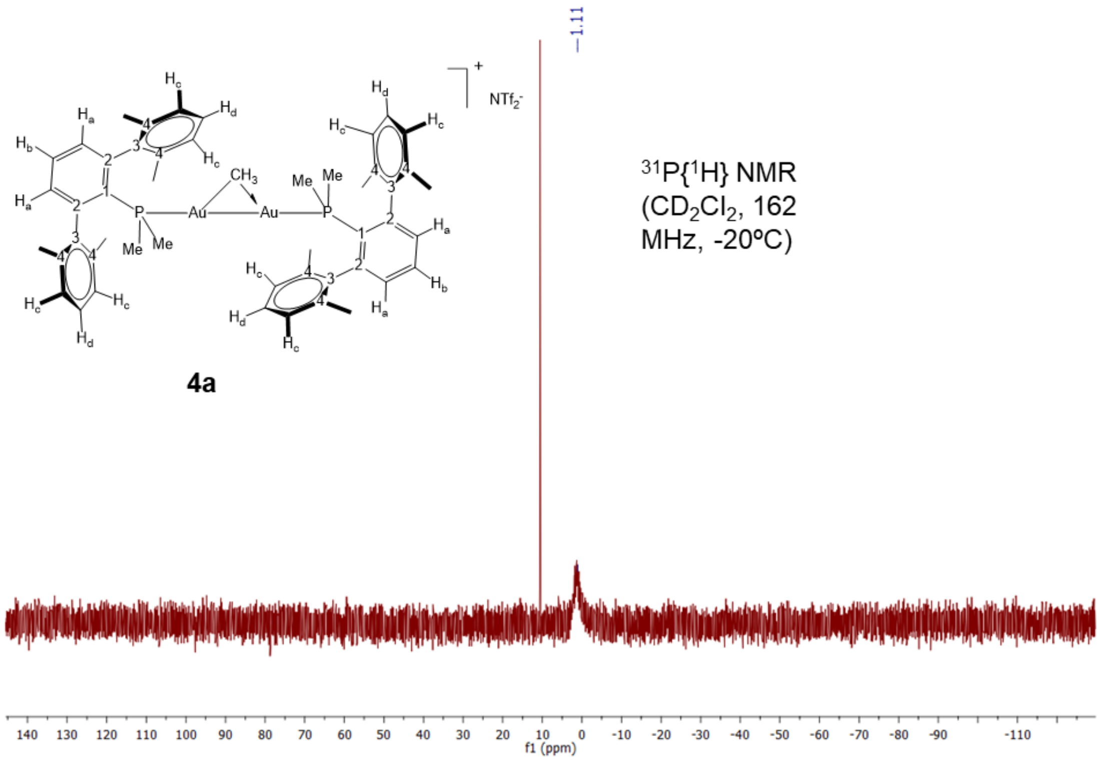

4a
NTf2-
31P{1H} NMR
(CD2Cl2, 162
MHz, -20ºC)
-1.11
f1 (ppm)

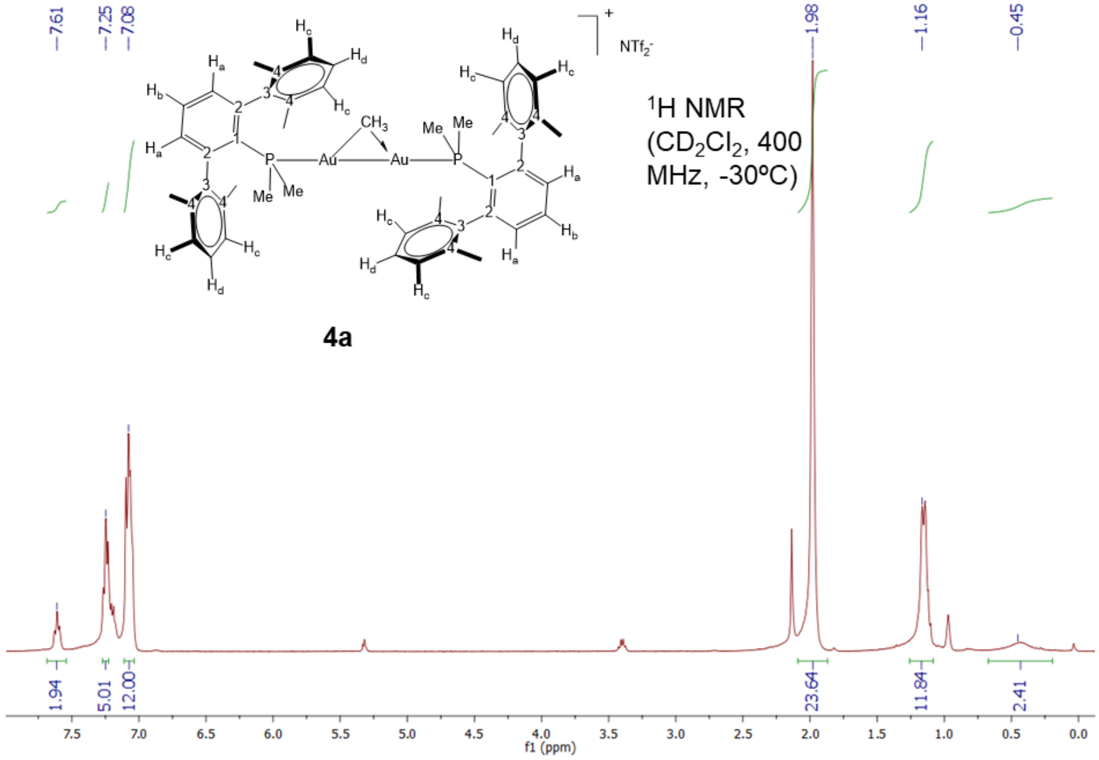

4a
NTf2-
1H NMR
(CD2Cl2, 400
MHz, -30ºC)
7.61
7.25
7.08
1.98
1.16
0.45
1.94
5.01
12.00
23.64
11.84
2.41
f1 (ppm)

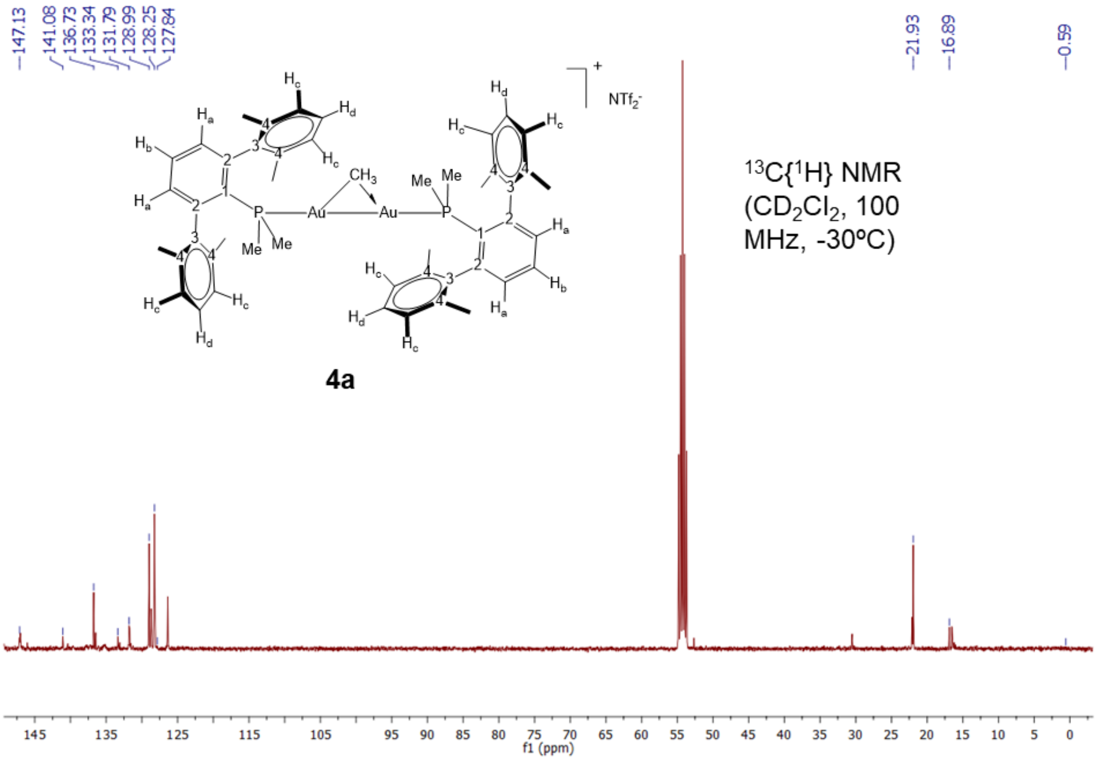
147.13
141.08
136.73
133.34
131.79
128.99
128.25
127.84
21.93
16.89
0.59
NTf2-
4a
13C{1H} NMR (CD2Cl2, 100 MHz, -30ºC)
f1 (ppm)

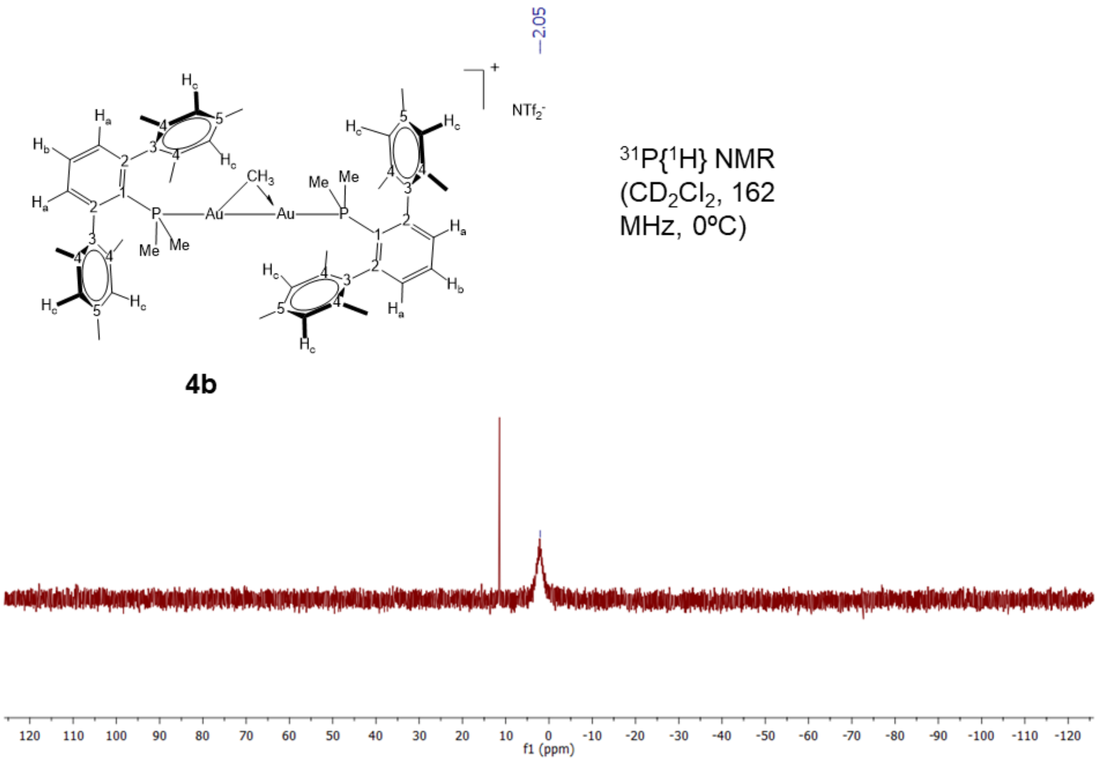
2.05
NTf2-
4b
31P{1H} NMR (CD2Cl2, 162 MHz, 0ºC)
f1 (ppm)

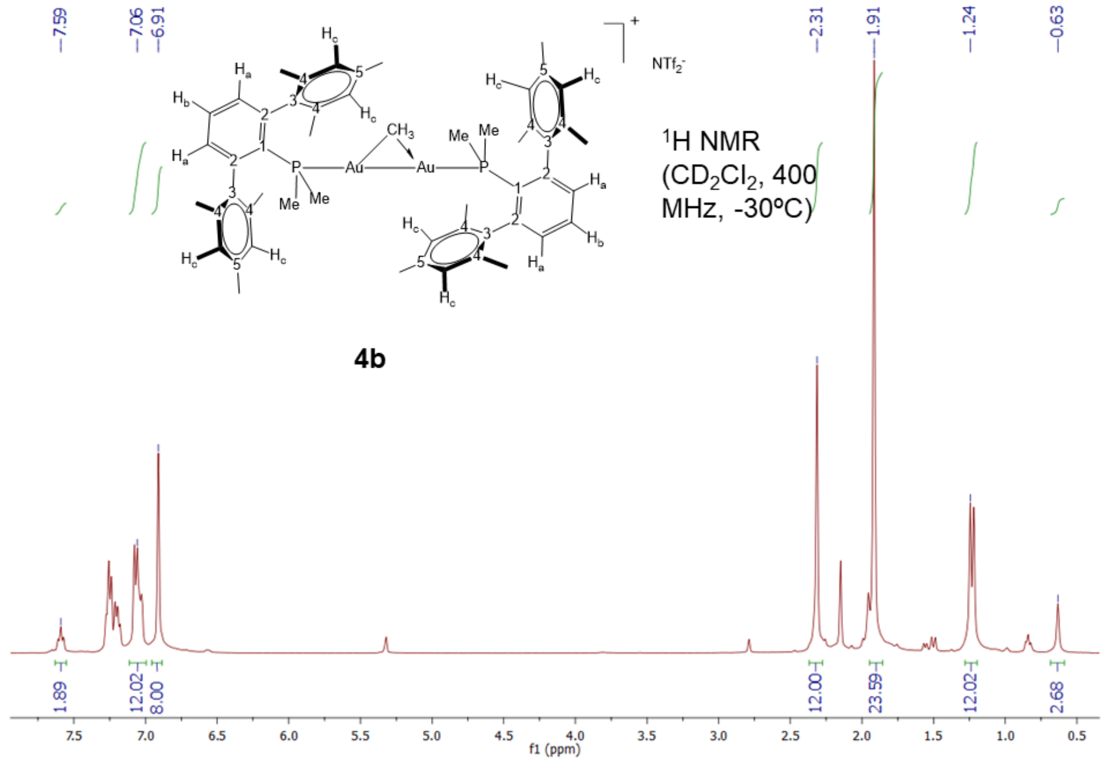

7.59
7.06
6.91
2.31
1.91
1.24
0.63
NTf2-
1H NMR
(CD2Cl2, 400 MHz, -30ºC)
4b
1.89
12.02
8.00
12.00
23.59
12.02
2.68
f1 (ppm)

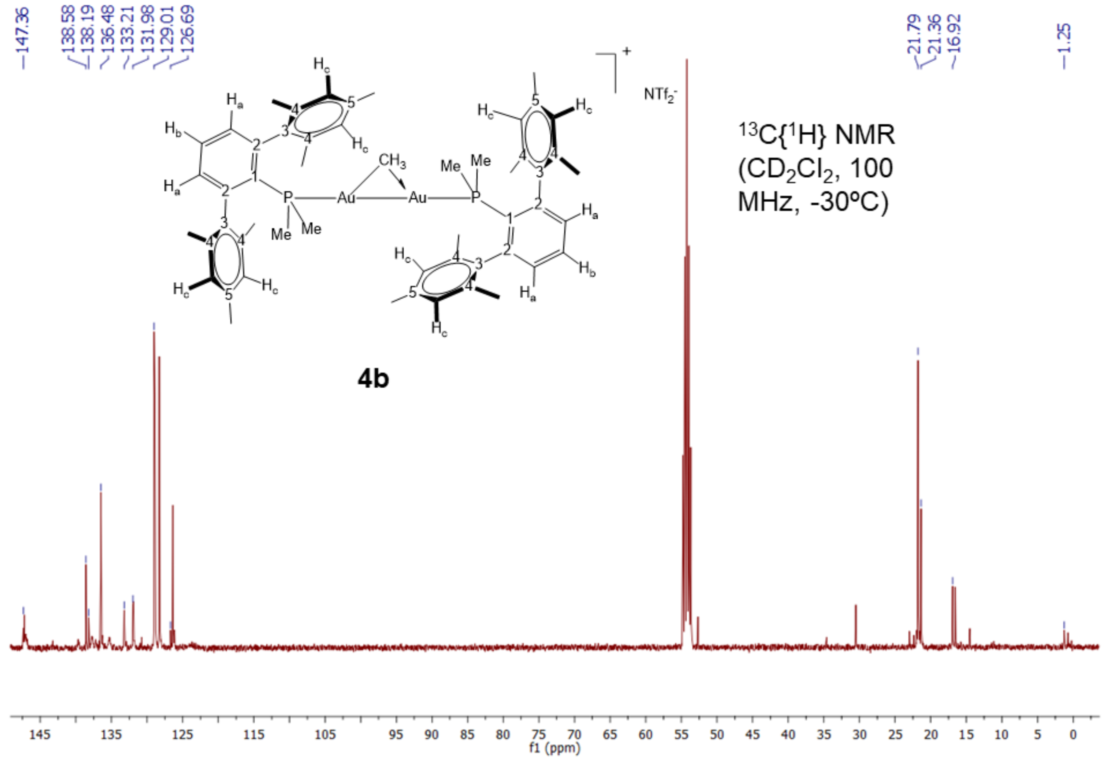

147.36
138.58
138.19
136.48
133.21
131.98
129.01
126.69
21.79
21.36
16.92
1.25
NTf2-
13C{1H} NMR
(CD2Cl2, 100 MHz, -30ºC)
4b
f1 (ppm)

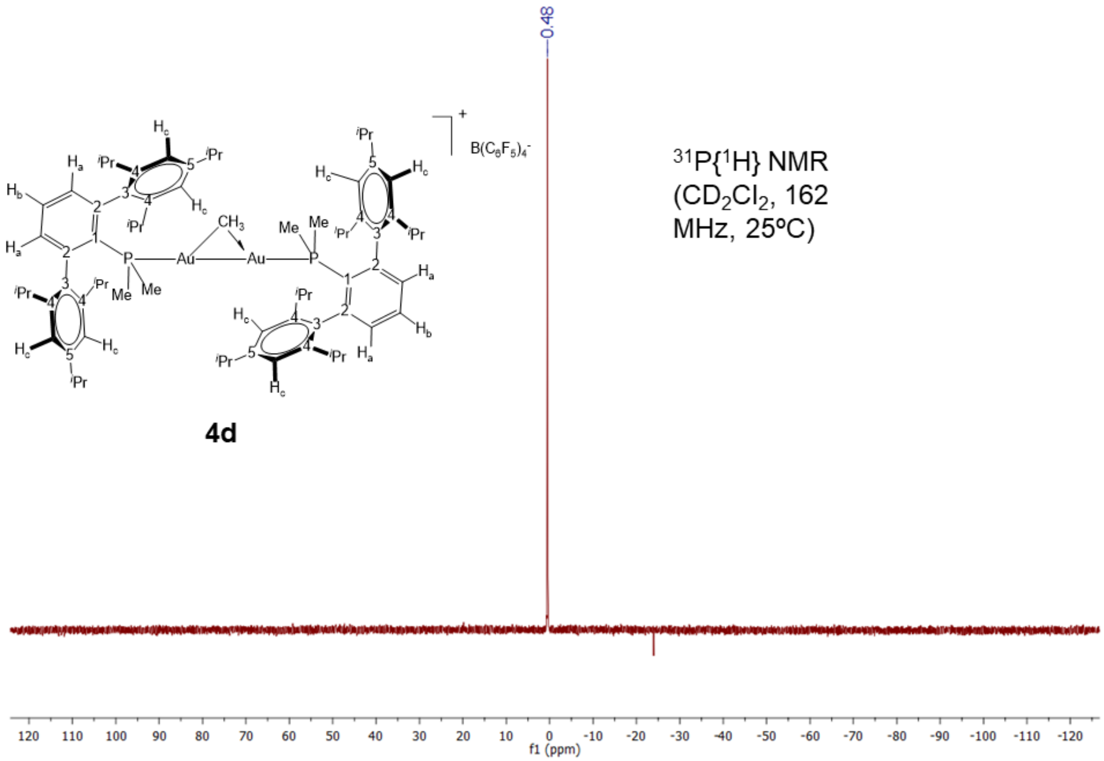

0.48
$^{31}P\{^1H\}$ NMR
(CD_2Cl_2, 162
MHz, 25ºC)
$B(C_6F_5)_4^-$
4d
f1 (ppm)

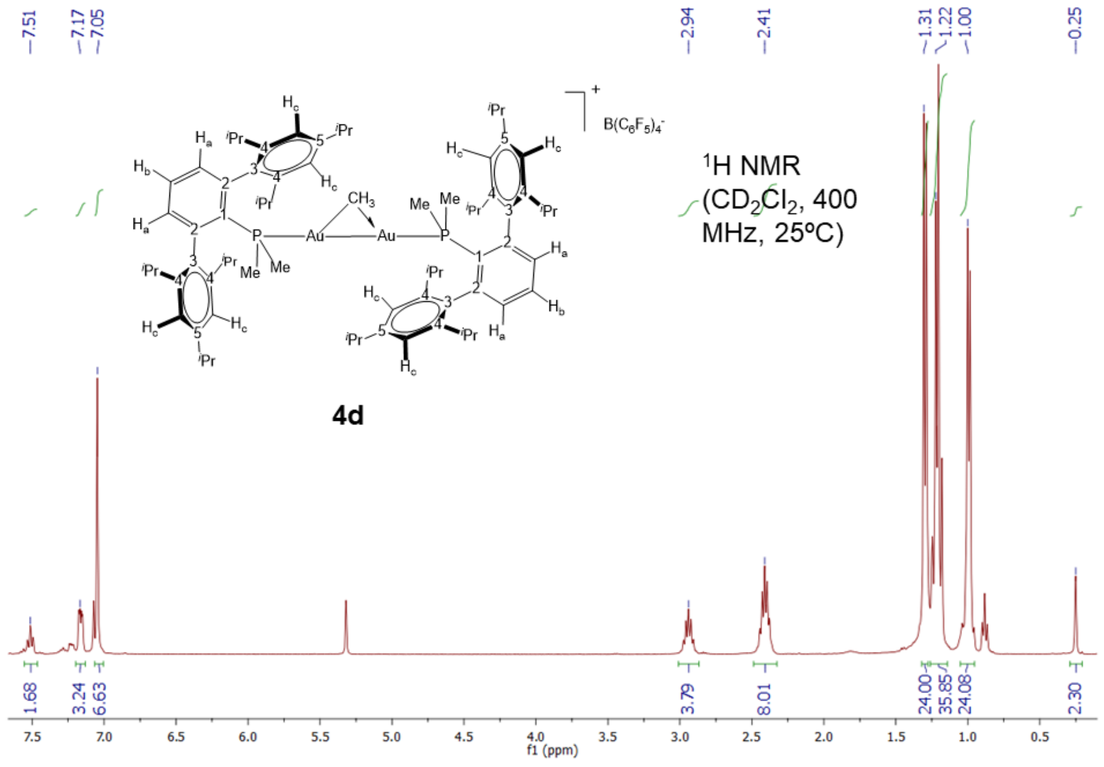

7.51
7.17
7.05
2.94
2.41
1.31
1.22
1.00
0.25
^1H NMR
(CD_2Cl_2, 400
MHz, 25ºC)
$B(C_6F_5)_4^-$
4d
1.68
3.24
6.63
3.79
8.01
24.00
35.85
24.08
2.30
f1 (ppm)

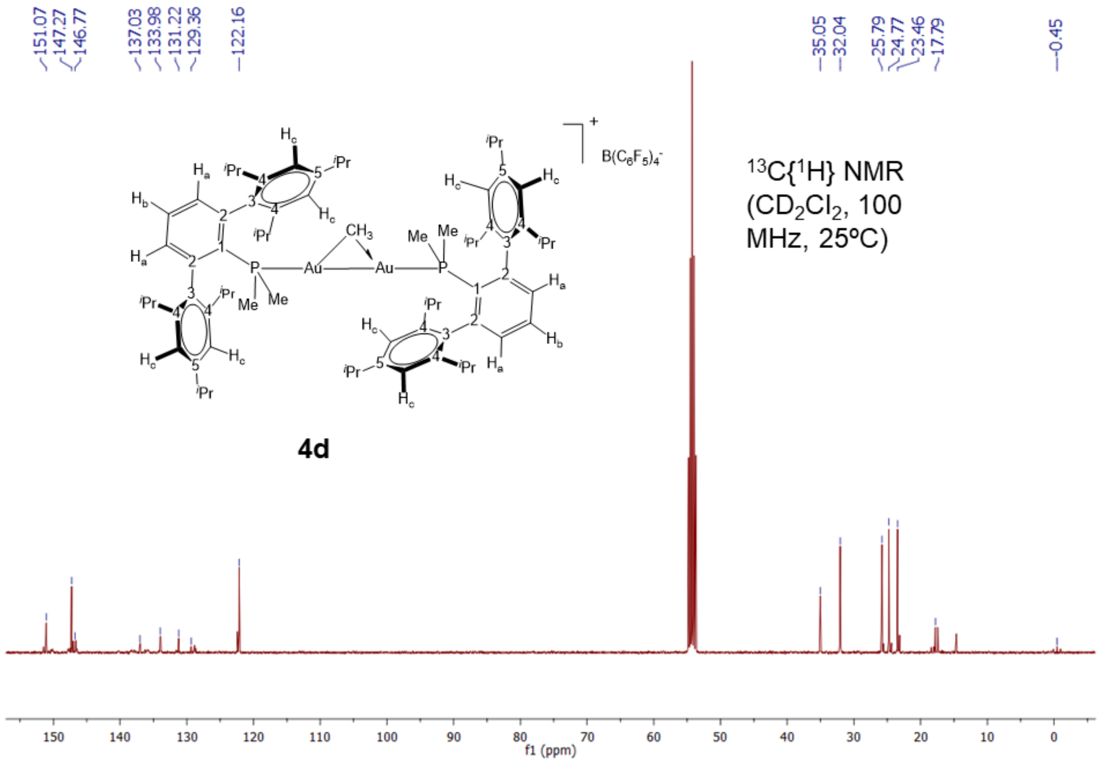
151.07
147.27
146.77
137.03
133.98
131.22
129.36
122.16
35.05
32.04
25.79
24.77
23.46
17.79
0.45
B(C6F5)4-
13C{1H} NMR (CD2Cl2, 100 MHz, 25ºC)
f1 (ppm)

4d

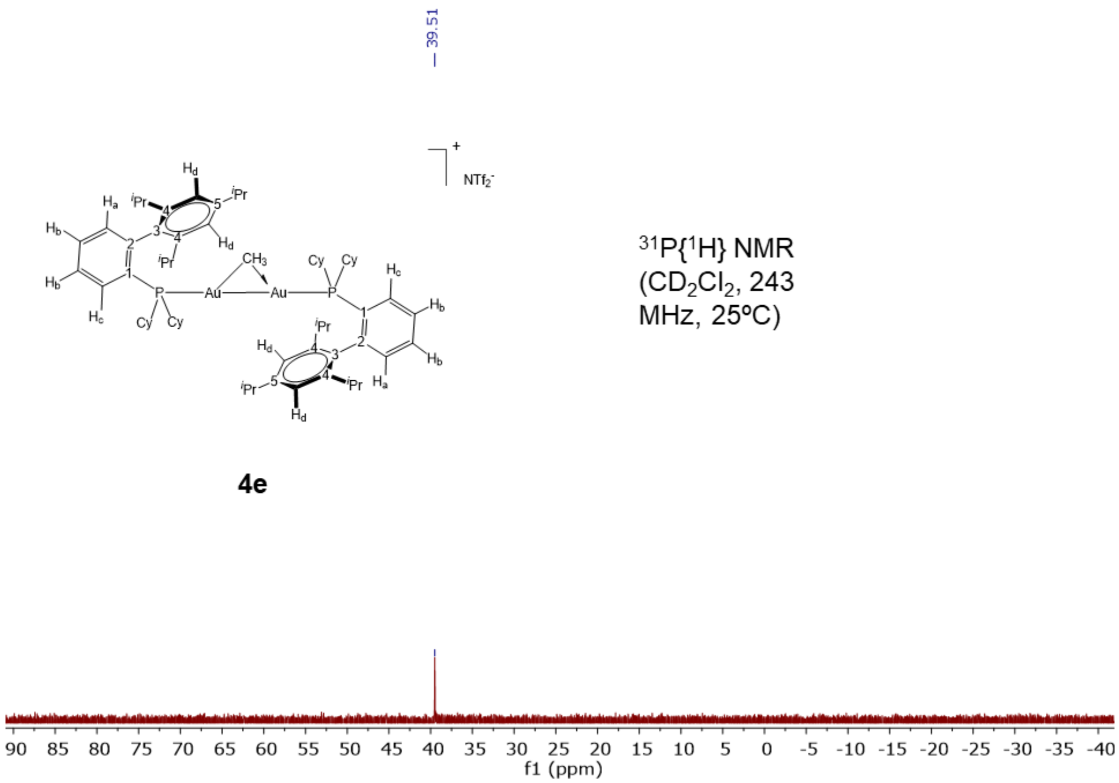
39.51
NTf2-
31P{1H} NMR (CD2Cl2, 243 MHz, 25ºC)
f1 (ppm)

4e

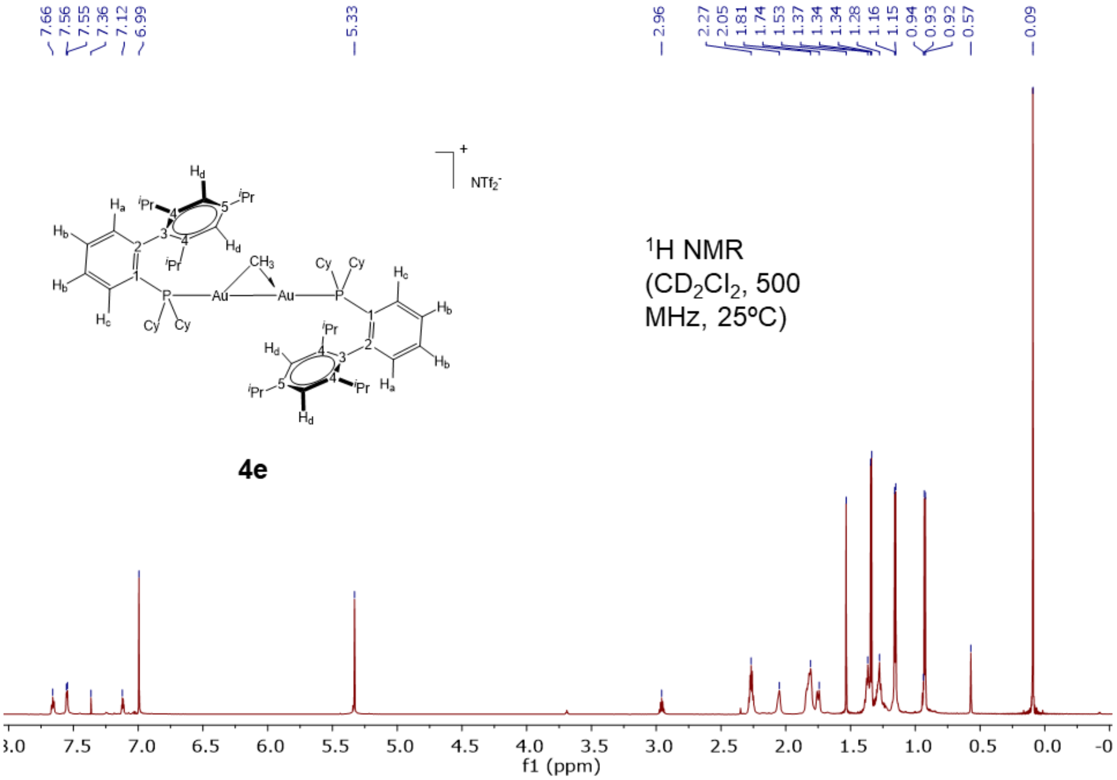

7.66
7.56
7.55
7.36
7.12
6.99
5.33
2.96
2.27
2.05
1.81
1.74
1.53
1.37
1.34
1.34
1.28
1.16
1.15
0.94
0.93
0.92
0.57
0.09
NTf2-
4e
1H NMR (CD2Cl2, 500 MHz, 25ºC)
f1 (ppm)

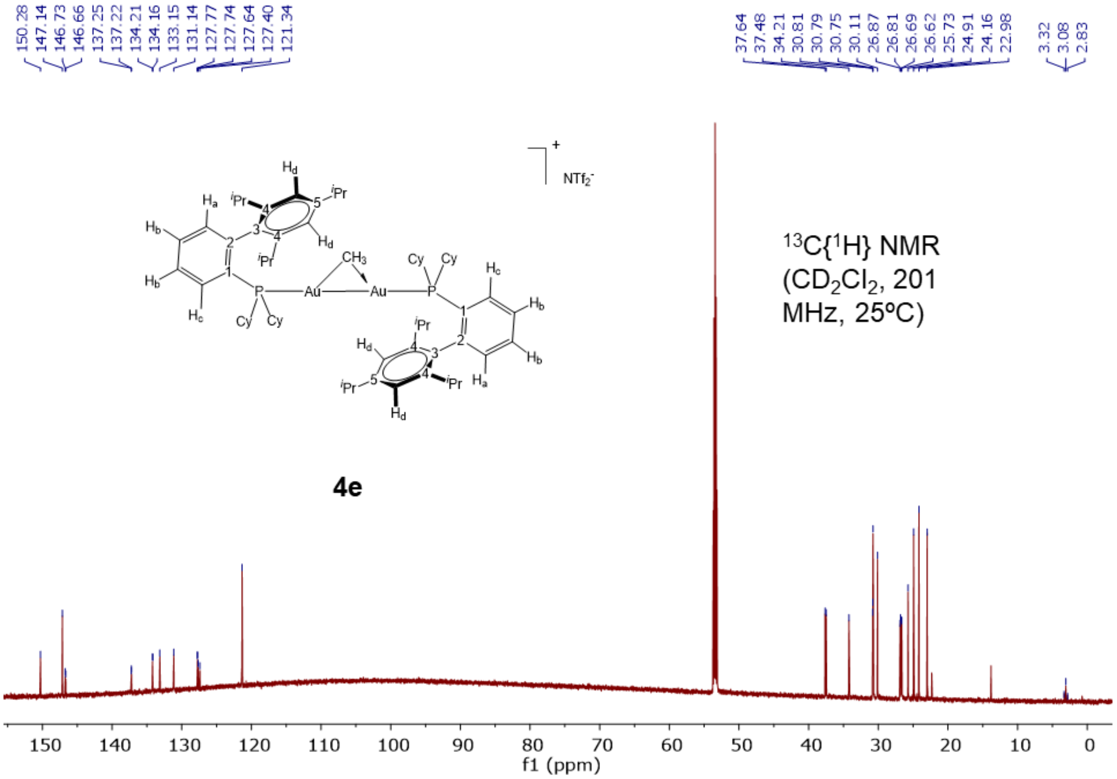

150.28
147.14
146.73
146.66
137.25
137.22
134.21
134.16
133.15
131.14
127.77
127.74
127.64
127.40
121.34
37.64
37.48
34.21
30.81
30.79
30.75
30.11
26.87
26.81
26.69
26.62
25.73
24.91
24.16
22.98
3.32
3.08
2.83
NTf2-
4e
13C{1H} NMR (CD2Cl2, 201 MHz, 25ºC)
f1 (ppm)

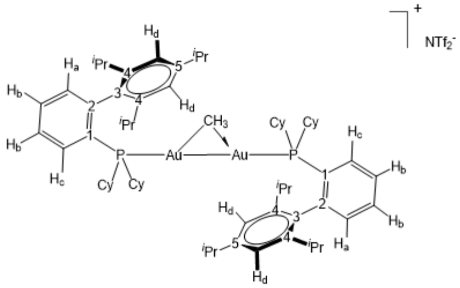

4e

$^{19}F\{^1H\}$ NMR (CD_2Cl_2, 565 MHz, 25 °C)

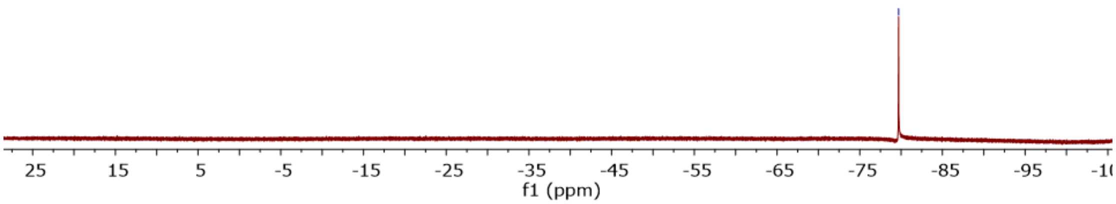

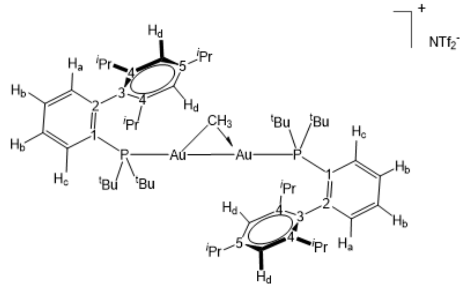

4f

$^{31}P\{^1H\}$ NMR (CD_2Cl_2, 243 MHz, 25ºC)

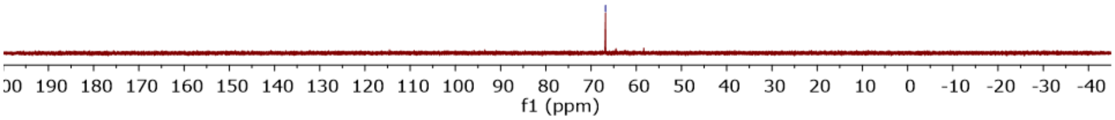

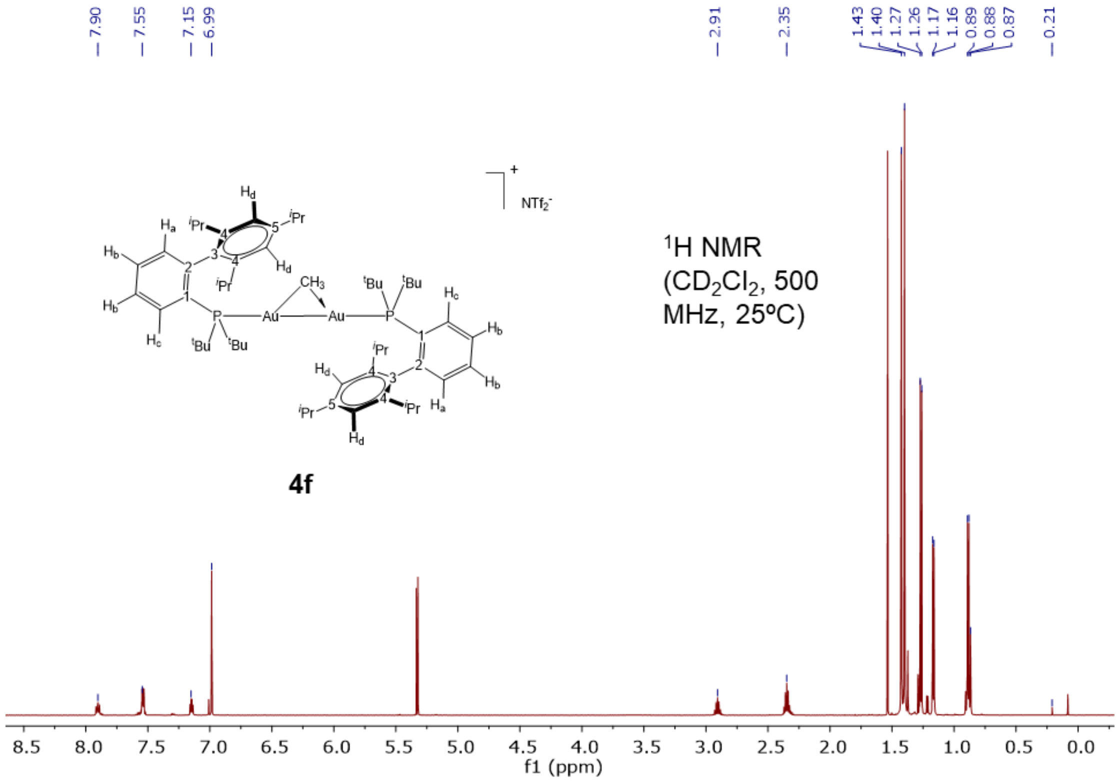

7.90
7.55
7.15
6.99
2.91
2.35
1.43
1.40
1.27
1.26
1.17
1.16
0.89
0.88
0.87
0.21
NTf2-
4f
1H NMR
(CD2Cl2, 500
MHz, 25ºC)
f1 (ppm)

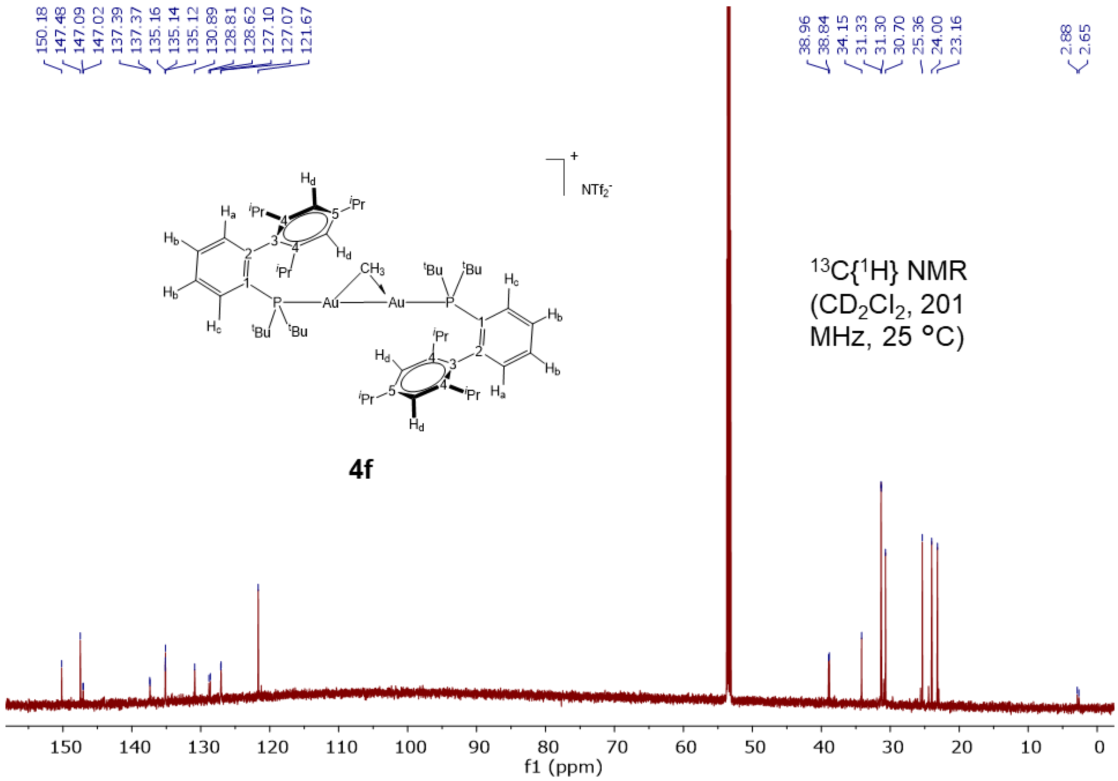

150.18
147.48
147.09
147.02
137.39
137.37
135.16
135.14
135.12
130.89
128.81
128.62
127.10
127.07
121.67
38.96
38.84
34.15
31.33
31.30
30.70
25.36
24.00
23.16
2.88
2.65
NTf2-
4f
13C{1H} NMR
(CD2Cl2, 201
MHz, 25 °C)
f1 (ppm)

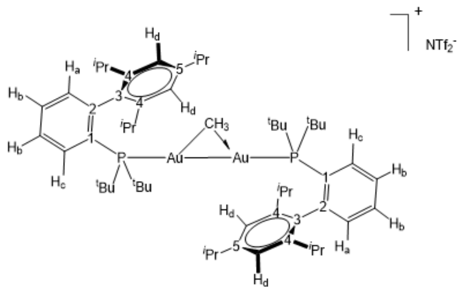

4f

$^{19}F\{^1H\}$ NMR (CD_2Cl_2, 565 MHz, 25 °C)

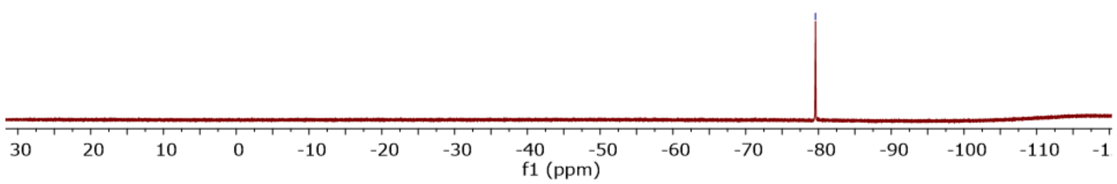

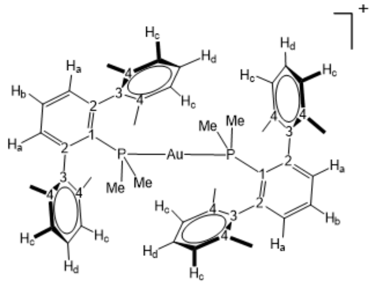

5a

$^{31}P\{^1H\}$ NMR (CD_2Cl_2, 162 MHz, 25ºC)

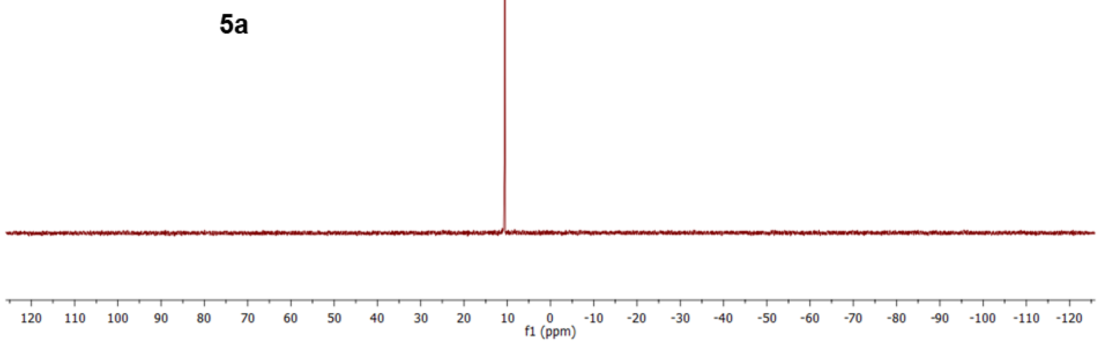

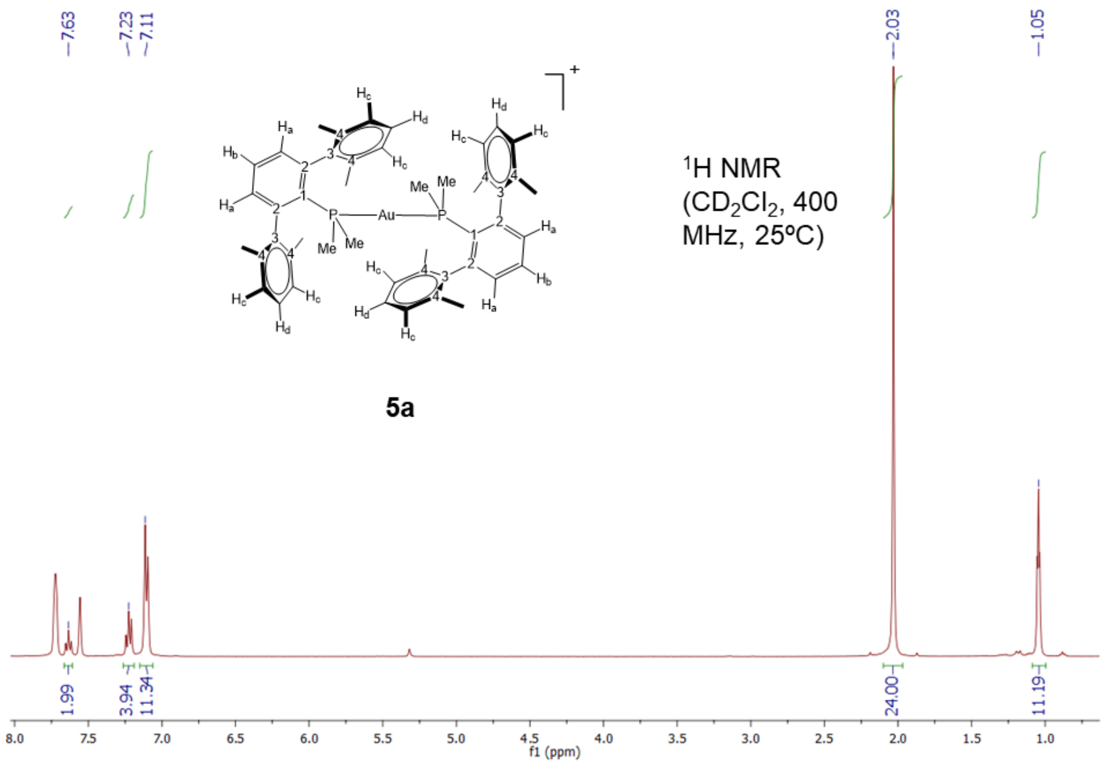
7.63
7.23
7.11
2.03
1.05
^{1}H NMR
(CD$_2$Cl$_2$, 400 MHz, 25ºC)
5a
1.99
3.94
11.34
24.00
11.19
f1 (ppm)

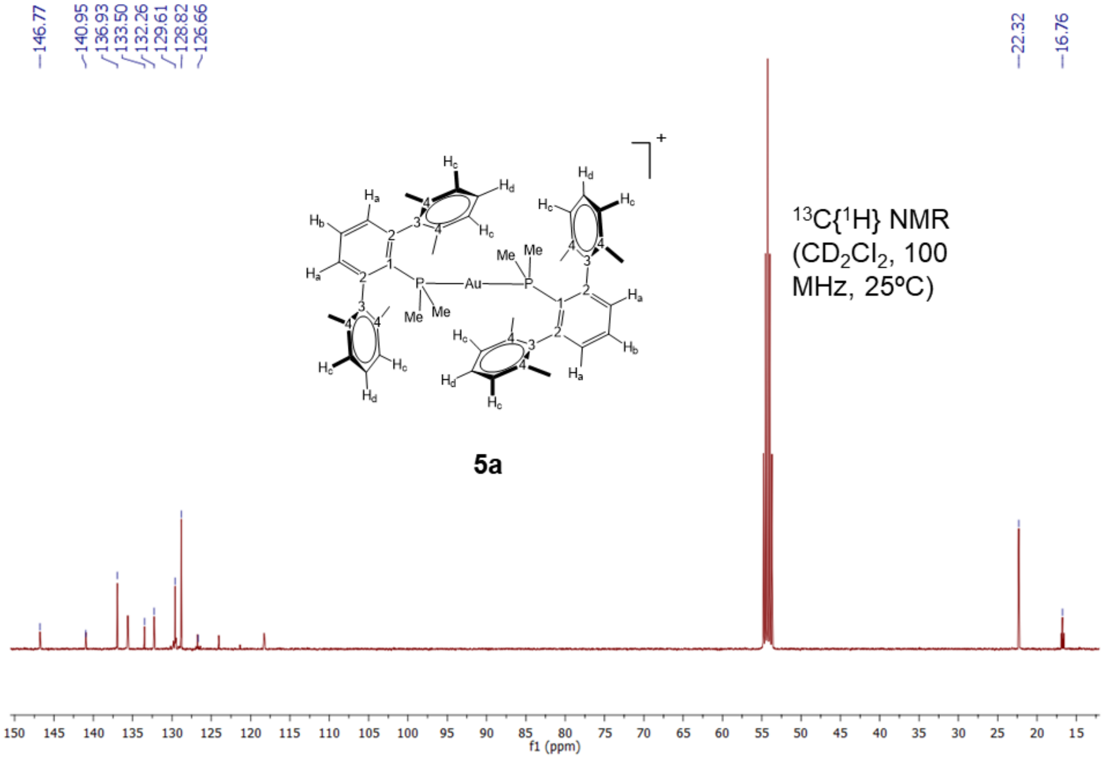
146.77
140.95
136.93
133.50
132.26
129.61
128.82
126.66
22.32
16.76
^{13}C{^{1}H} NMR
(CD$_2$Cl$_2$, 100 MHz, 25ºC)
5a
f1 (ppm)

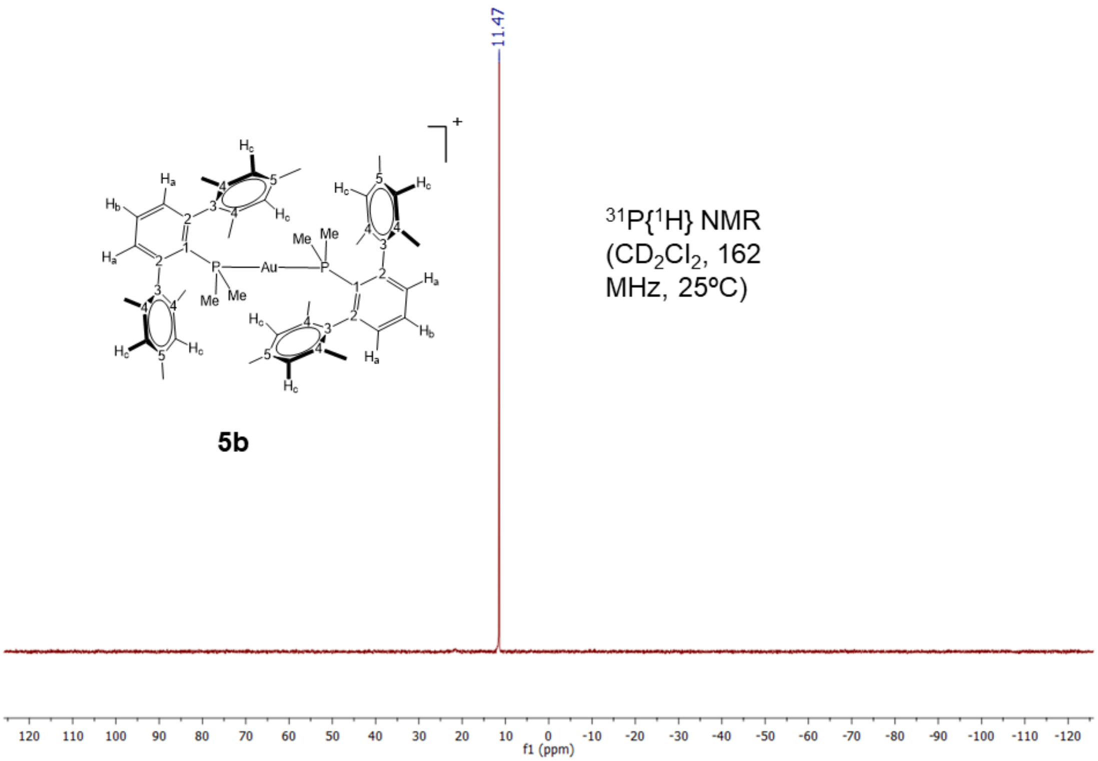
11.47
5b
$^{31}P\{^1H\}$ NMR
(CD_2Cl_2, 162
MHz, 25ºC)
f1 (ppm)

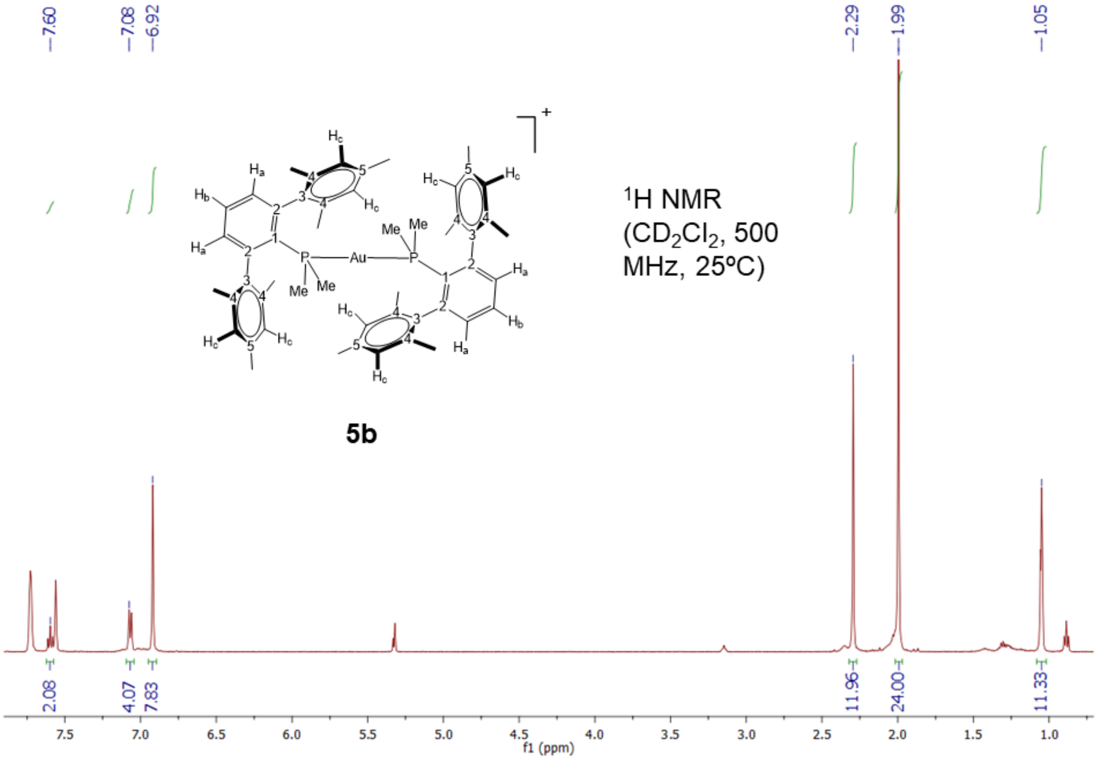
7.60
7.08
6.92
2.29
1.99
1.05
5b
^1H NMR
(CD_2Cl_2, 500
MHz, 25ºC)
2.08
4.07
7.83
11.96
24.00
11.33
f1 (ppm)

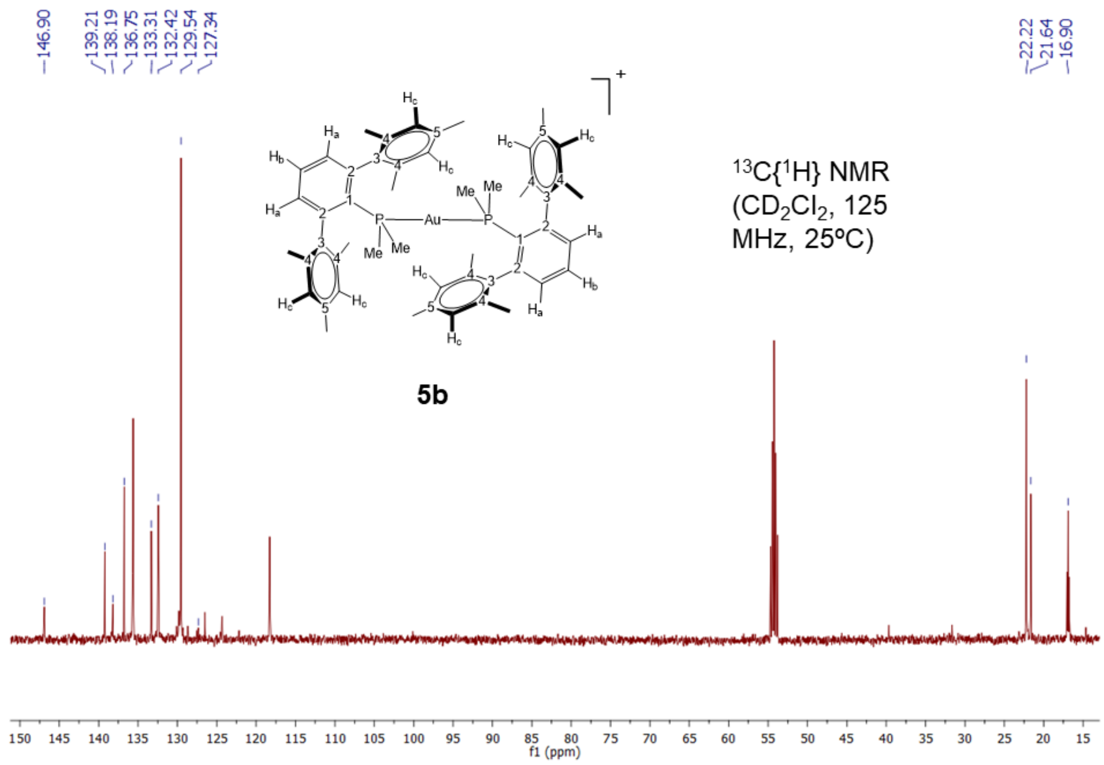
146.90
139.21
138.19
136.75
133.31
132.42
129.54
127.34
22.22
21.64
16.90
5b
$^{13}C\{^1H\}$ NMR
(CD_2Cl_2, 125
MHz, 25ºC)
150 145 140 135 130 125 120 115 110 105 100 95 90 85 80 75 70 65 60 55 50 45 40 35 30 25 20 15
f1 (ppm)

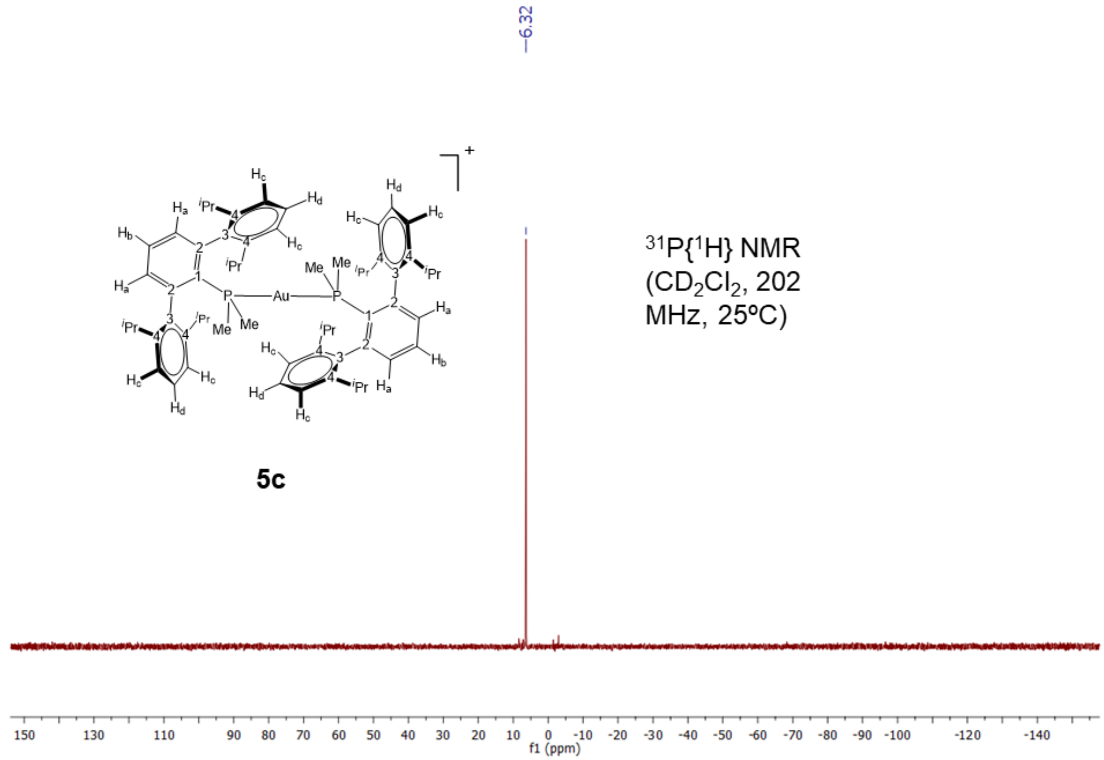
6.32
5c
$^{31}P\{^1H\}$ NMR
(CD_2Cl_2, 202
MHz, 25ºC)
150 130 110 90 80 70 60 50 40 30 20 10 0 -10 -20 -30 -40 -50 -60 -70 -80 -90 -100 -120 -140
f1 (ppm)

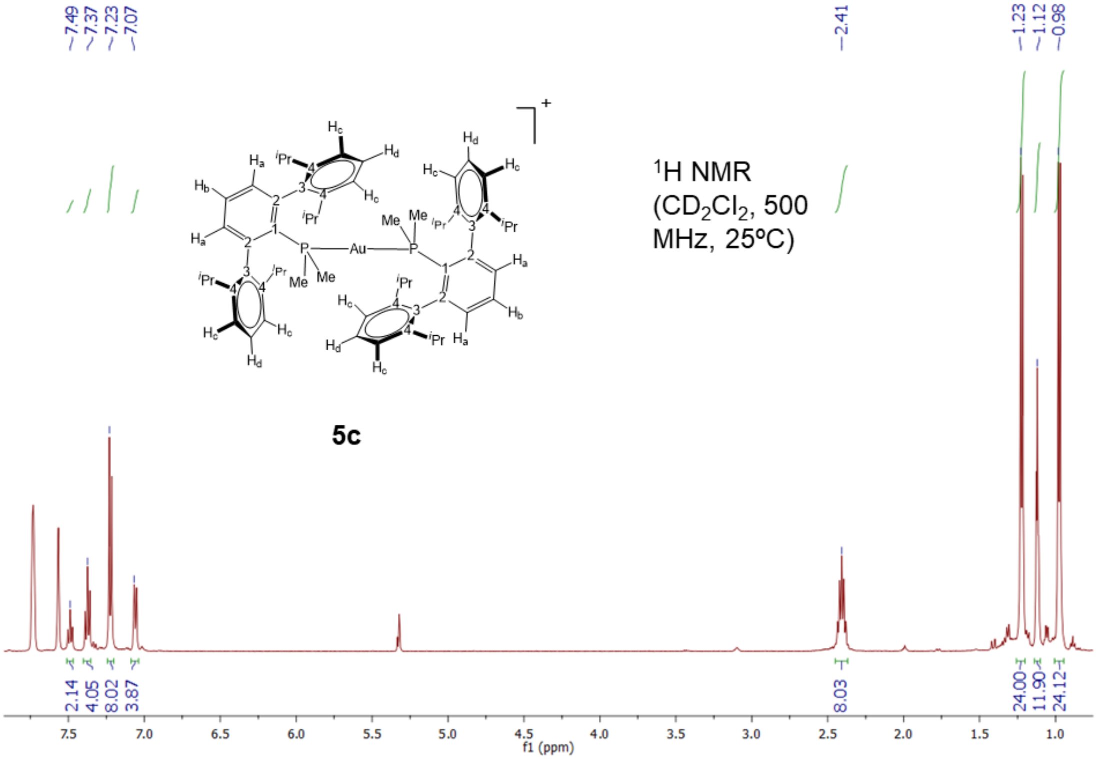

7.49
7.37
7.23
7.07
2.41
1.23
1.12
0.98
1H NMR
(CD2Cl2, 500
MHz, 25ºC)
5c
2.14
4.05
8.02
3.87
8.03
24.00
11.90
24.12
7.5
7.0
6.5
6.0
5.5
5.0
4.5
4.0
3.5
3.0
2.5
2.0
1.5
1.0
f1 (ppm)

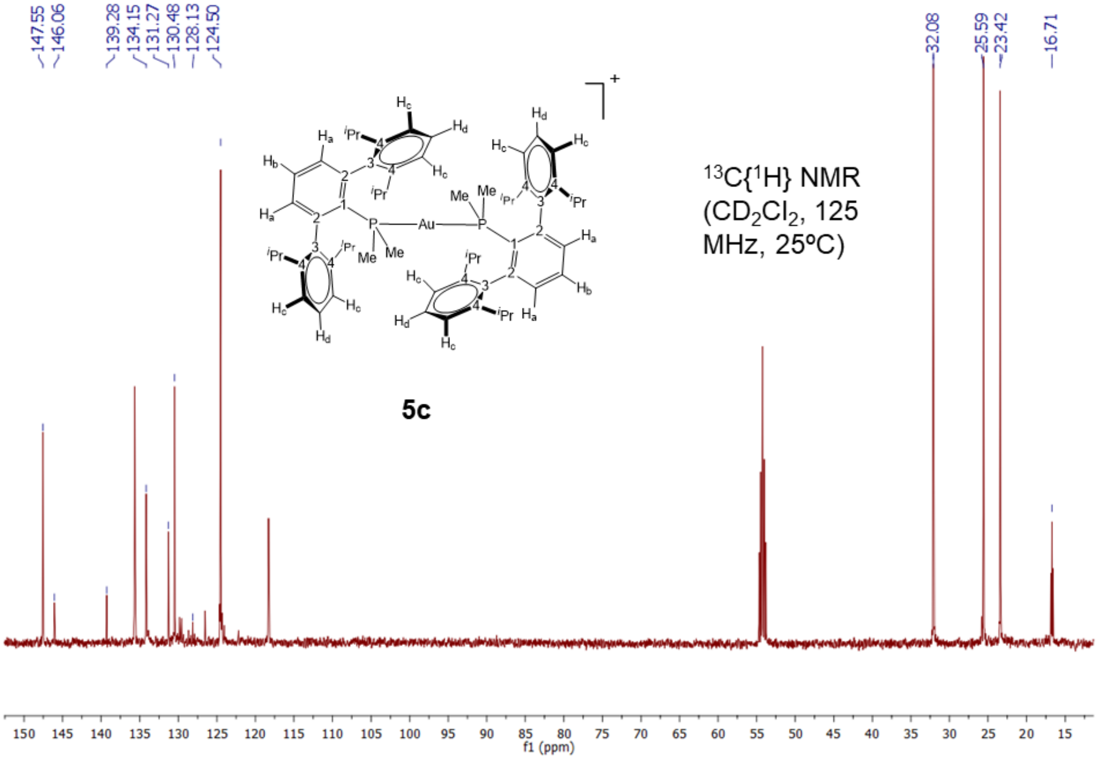

147.55
146.06
139.28
134.15
131.27
130.48
128.13
124.50
32.08
25.59
23.42
16.71
13C{1H} NMR
(CD2Cl2, 125
MHz, 25ºC)
5c
150
145
140
135
130
125
120
115
110
105
100
95
90
85
80
75
70
65
60
55
50
45
40
35
30
25
20
15
f1 (ppm)

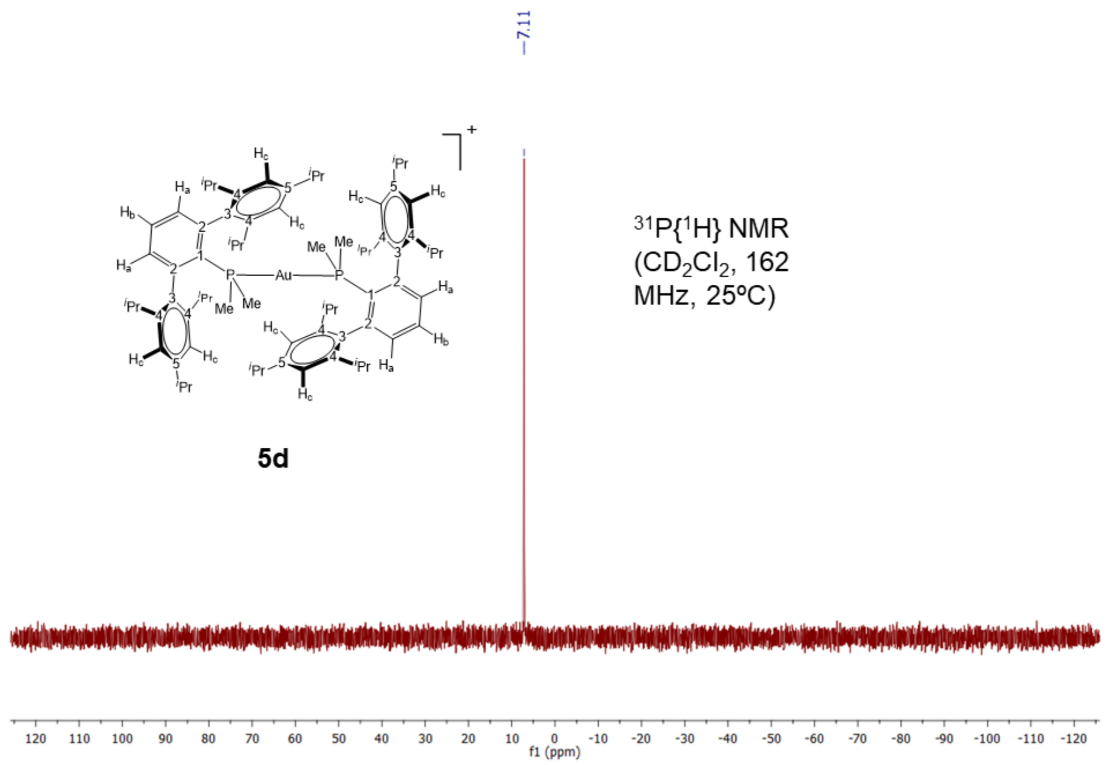

7.11
5d
31P{1H} NMR
(CD2Cl2, 162
MHz, 25ºC)
120 110 100 90 80 70 60 50 40 30 20 10 0 -10 -20 -30 -40 -50 -60 -70 -80 -90 -100 -110 -120
f1 (ppm)

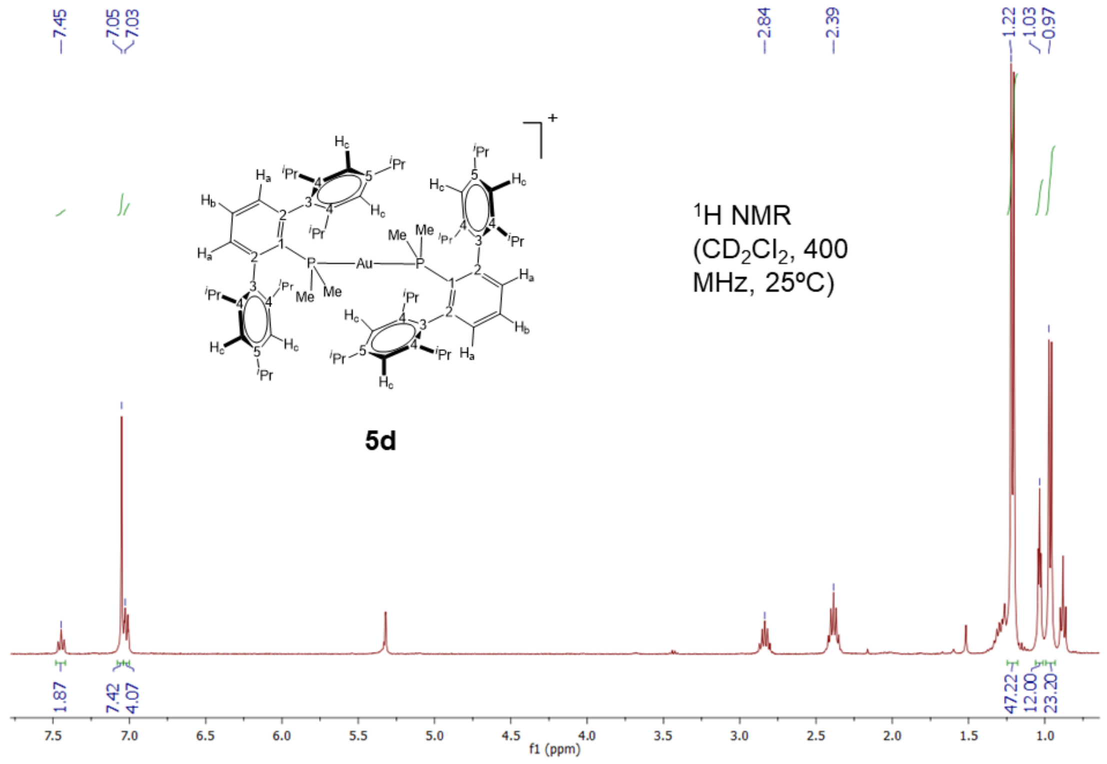

7.45
7.05
7.03
2.84
2.39
1.22
1.03
0.97
5d
1H NMR
(CD2Cl2, 400
MHz, 25ºC)
1.87
7.42
4.07
47.22
12.00
23.20
7.5 7.0 6.5 6.0 5.5 5.0 4.5 4.0 3.5 3.0 2.5 2.0 1.5 1.0
f1 (ppm)

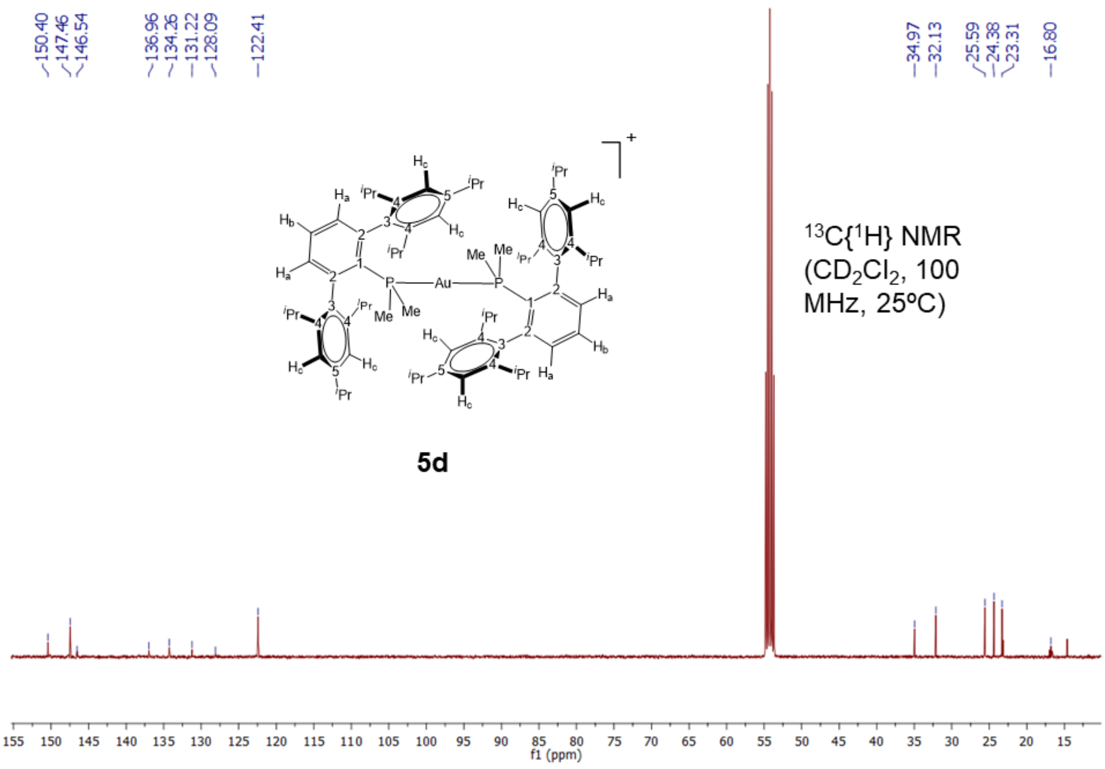
150.40
147.46
146.54
136.96
134.26
131.22
128.09
122.41
34.97
32.13
25.59
24.38
23.31
16.80
13C{1H} NMR
(CD2Cl2, 100
MHz, 25ºC)
5d
155 150 145 140 135 130 125 120 115 110 105 100 95 90 85 80 75 70 65 60 55 50 45 40 35 30 25 20 15
f1 (ppm)

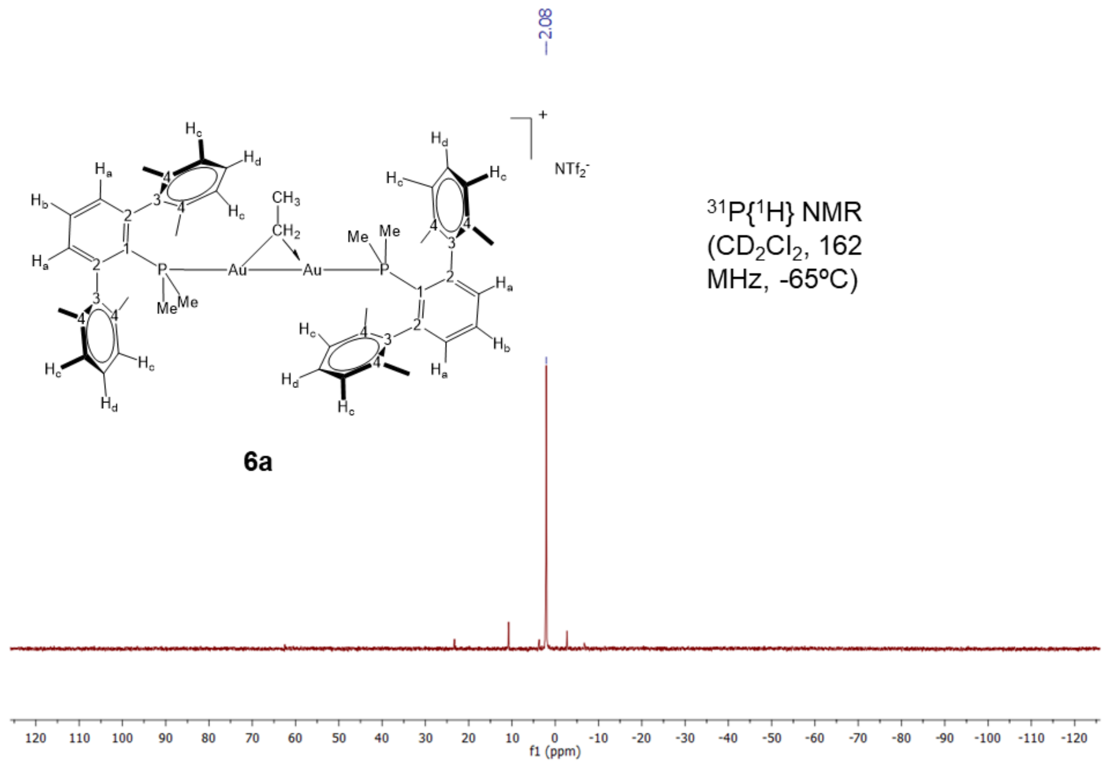
2.08
NTf2-
31P{1H} NMR
(CD2Cl2, 162
MHz, -65ºC)
6a
120 110 100 90 80 70 60 50 40 30 20 10 0 -10 -20 -30 -40 -50 -60 -70 -80 -90 -100 -110 -120
f1 (ppm)

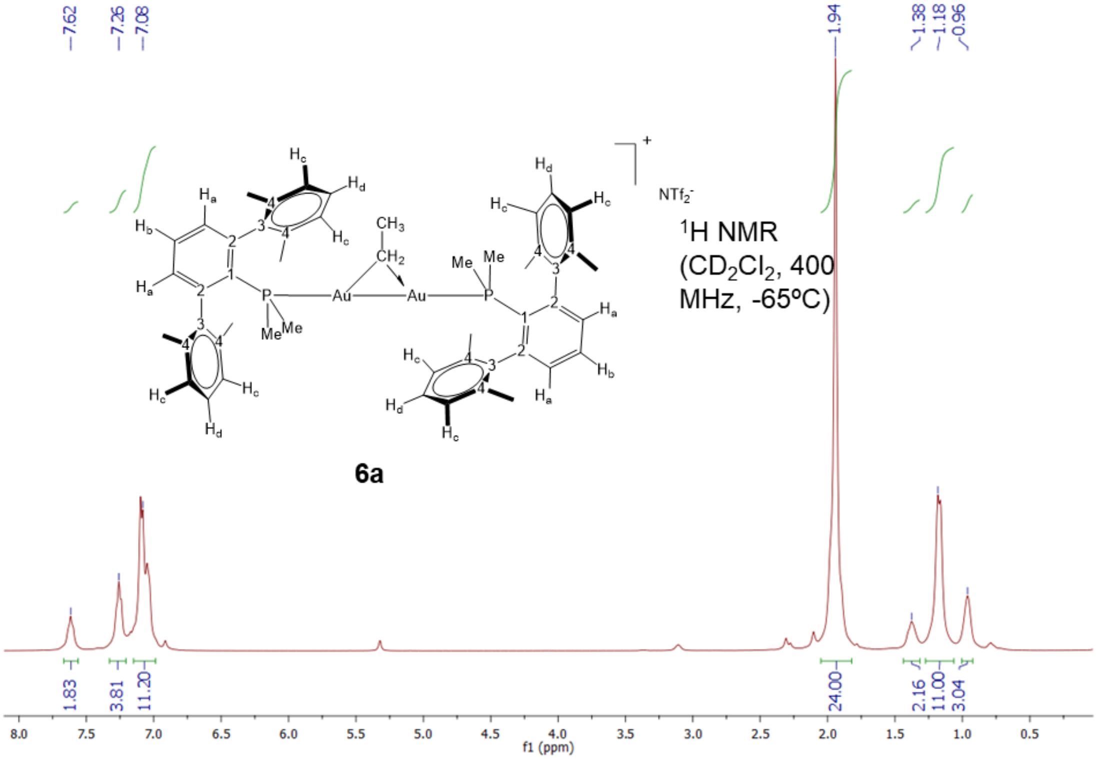

7.62
7.26
7.08
1.94
1.38
1.18
0.96
NTf2-
6a
1H NMR (CD2Cl2, 400 MHz, -65ºC)
1.83
3.81
11.20
24.00
2.16
11.00
3.04
f1 (ppm)

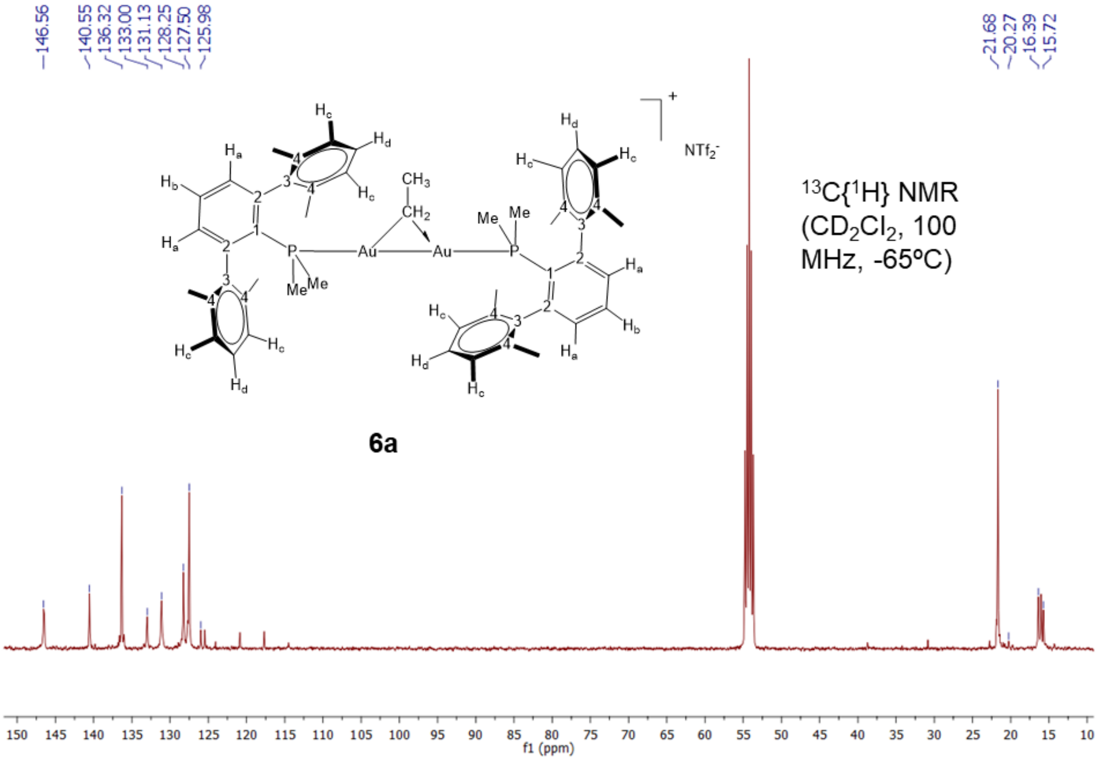

146.56
140.55
136.32
133.00
131.13
128.25
127.50
125.98
21.68
20.27
16.39
15.72
NTf2-
6a
13C{1H} NMR (CD2Cl2, 100 MHz, -65ºC)
f1 (ppm)

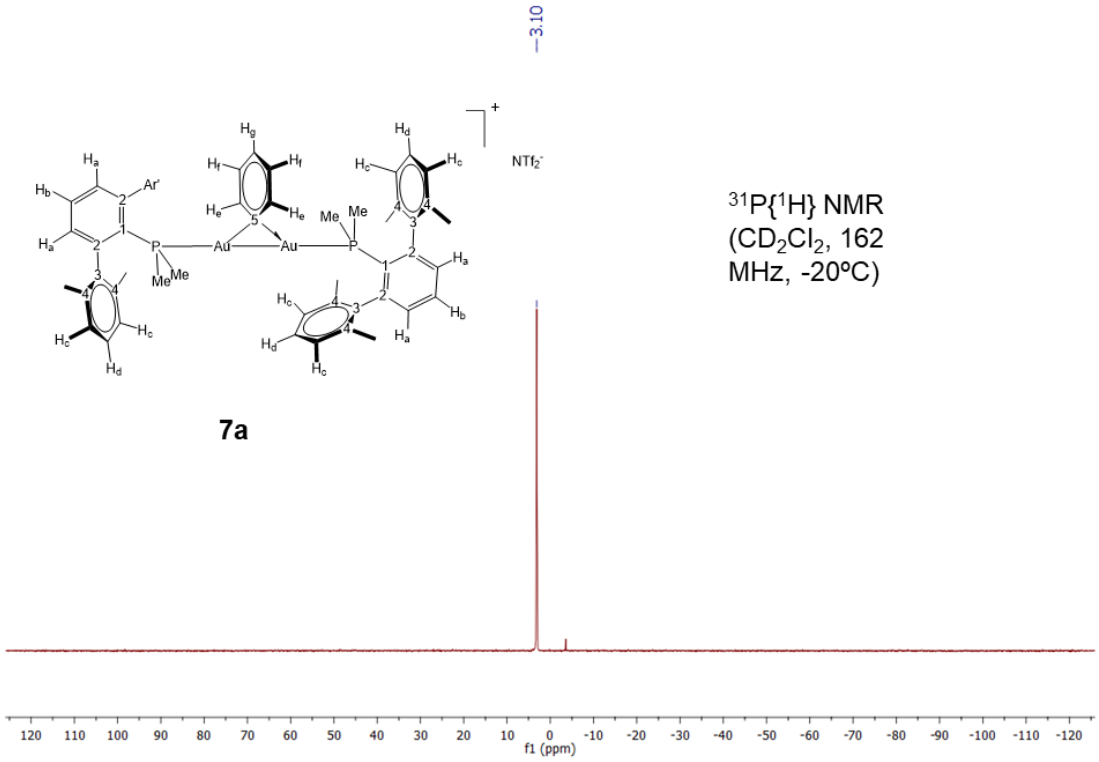

-3.10
NTf2-
7a
31P{1H} NMR
(CD2Cl2, 162
MHz, -20ºC)
f1 (ppm)

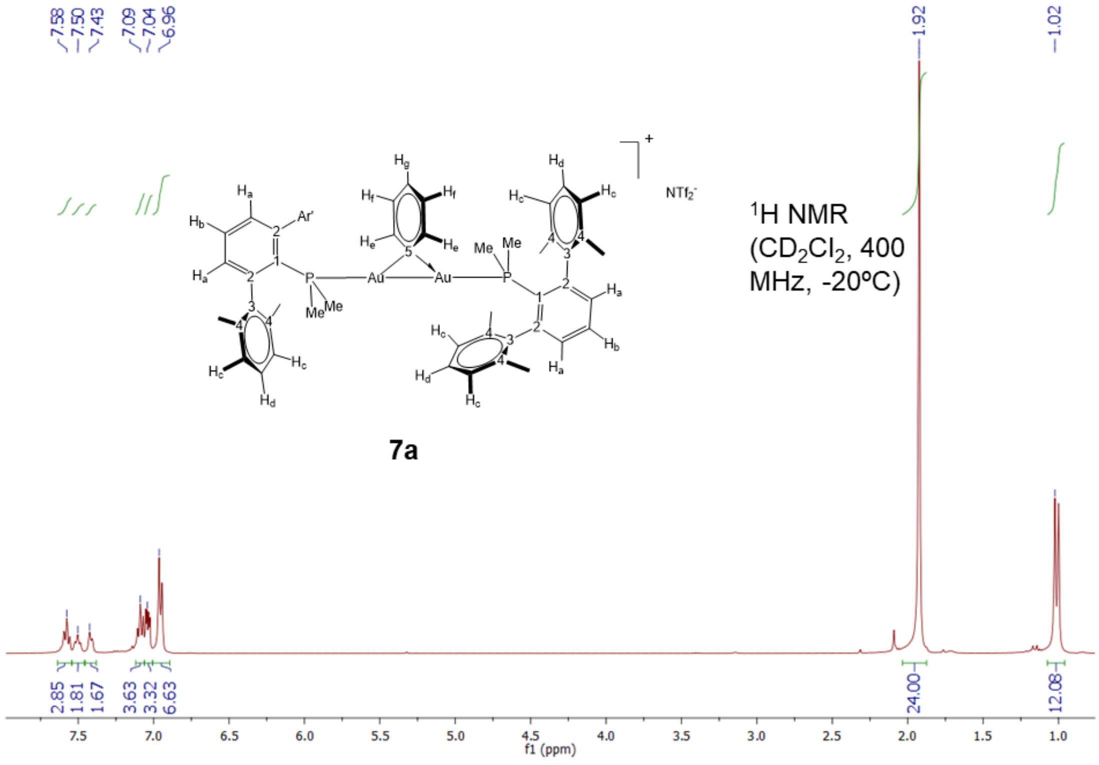

7.58
7.50
7.43
7.09
7.04
6.96
1.92
1.02
NTf2-
7a
1H NMR
(CD2Cl2, 400
MHz, -20ºC)
2.85
1.81
1.67
3.63
3.32
6.63
24.00
12.08
f1 (ppm)

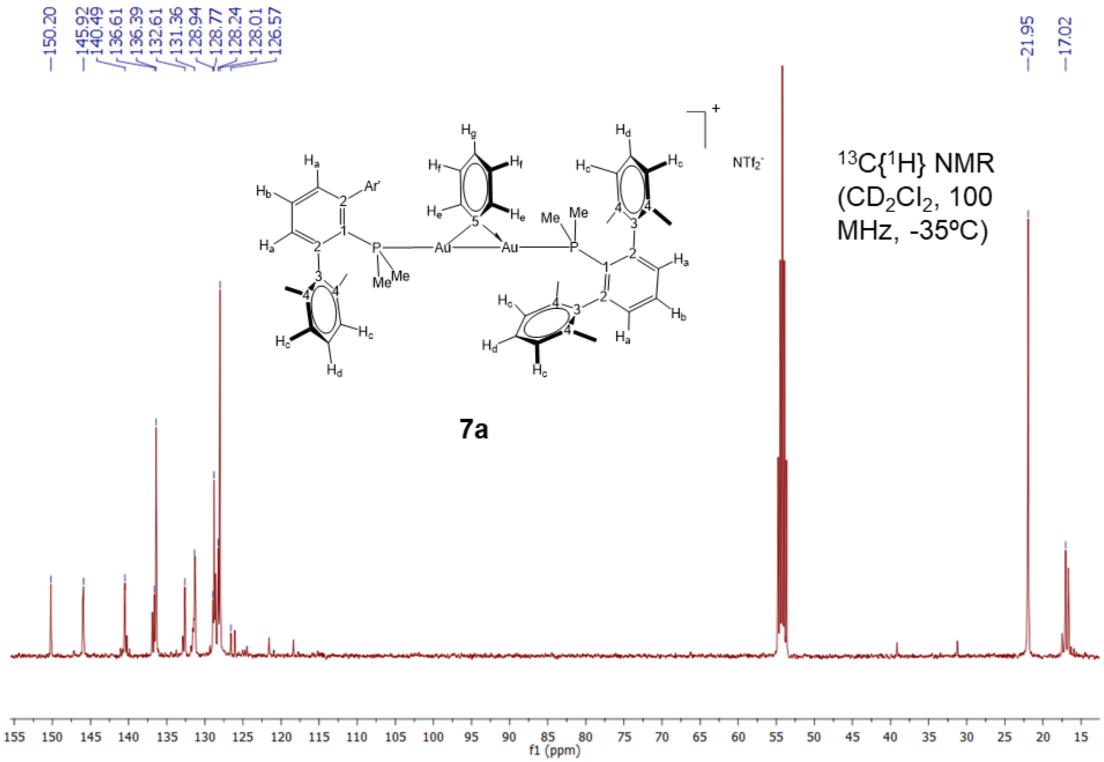
150.20
145.92
140.49
136.61
136.39
132.61
131.36
128.94
128.77
128.24
128.01
126.57
21.95
17.02
$^{13}C\{^1H\}$ NMR
(CD_2Cl_2, 100
MHz, -35ºC)
7a
Ar'
Au
Au
P
Me
NTf_2^-
155 150 145 140 135 130 125 120 115 110 105 100 95 90 85 80 75 70 65 60 55 50 45 40 35 30 25 20 15
f1 (ppm)

References

1. C. Nieto-Oberhuber, S. López and A. M. Echavarren, *J. Am. Chem. Soc.*, 2005, **127**, 6178-6179.

2. G. M. Sheldrick, *Acta Cryst.* A., 2015, **71**, 3-8.

3. Bruker (2012). *Saint; SADABS; APEX3*. Bruker AXS Inc., Madison, Wisconsin, USA.

4. O. V. Dolomanov, L. J. Bourhis, R. J. Gildea, J. A. K. Howard, H. Puschmann, *J. Appl. Cryst.* 2009, **42**, 339-341.

5. Gaussian 09, Revision E.01, M. J. Frisch, G. W. Trucks, H. B. Schlegel, G. E. Scuseria, M. A. Robb, J. R. Cheeseman, G. Scalmani, V. Barone, B. Mennucci, G. A. Petersson, H. Nakatsuji, M. Caricato, X. Li, H. P. Hratchian, A. F. Izmaylov, J. Bloino, G. Zheng, J. L. Sonnenberg, M. Hada, M. Ehara, K. Toyota, R. Fukuda, J. Hasegawa, M. Ishida, T. Nakajima, Y. Honda, O. Kitao, H. Nakai, T. Vreven, J. A. Montgomery, Jr., J. E. Peralta, F. Ogliaro, M. Bearpark, J. J. Heyd, E. Brothers, K. N. Kudin, V. N. Staroverov, T. Keith, R. Kobayashi, J. Normand, K. Raghavachari, A. Rendell, J. C. Burant, S. S. Iyengar, J. Tomasi, M. Cossi, N. Rega, J. M. Millam, M. Klene, J. E. Knox, J. B. Cross, V. Bakken, C. Adamo, J. Jaramillo, R. Gomperts, R. E. Stratmann, O. Yazyev, A. J. Austin, R. Cammi, C. Pomelli, J. W. Ochterski, R. L. Martin, K. Morokuma, V. G. Zakrzewski, G. A. Voth, P. Salvador, J. J. Dannenberg, S. Dapprich, A. D. Daniels, O. Farkas, J. B. Foresman, J. V. Ortiz, J. Cioslowski, and D. J. Fox, Gaussian, Inc., Wallingford CT, 2013.

6. J.-D. Chai, M. Head-Gordon, *Phys. Chem. Chem. Phys*., 2008, **10**, 6615.

7. a) W. J. Hehre, R. Ditchfield, J. A. Pople, *J. Phys. Chem.,* 1972, **56**, 2257; b) P. C. Hariharan, J. A. Pople, *Theor. Chim. Acta.,* 1973, **28**, 213; c) M. M. Francl, W. J. Pietro, W. J. Hehre, J. S. Binkley, M. S. Gordon, D. J. Defrees, J. A. Pople, *J. Chem. Phys.,* 1982, **77**, 3654.

8. D. Andrae, U. Haeussermann, M. Dolg, H. Stoll, H. Preuss, *Theor. Chim. Acta.,* 1990, **77**, 123.

9. A. V. Marenich, C. J. Cramer, D. G. Truhlar, *J. Phys. Chem. B*., 2009, **113**, 6378.

10. R. F. Ribeiro, A. V. Marenich, C. J. Cramer, D. G. Truhlar, *J. Phys. Chem. B.,* 2011, **115**, 14556.

11. I. Funes-Ardoiz, R. S. Paton, 2016 Goodvibes: Goodvibes 2.0.2 https://doi.org/10.5281/zenodo.595246
